# Supplementary material for: Completeness and validity of alcohol recording in general practice within the UK: a cross-sectional study
Source: BMJ Open. 2019 Nov 26;9(11):e031537. doi: 10.1136/bmjopen-2019-031537 (PMC6887039; doi:10.1136/bmjopen-2019-031537)
Supplement: Supplementary data [file bmjopen-2019-031537supp001.pdf]

Appendices

Supplementary Text 1. Detailed alcohol-use definitions

i. Any code suggesting that alcohol was discussed in the consultation (**Supplementary Table 1**, Column 1).
ii. Codes indicating AUDIT or FAST screening (**Supplementary Table 1**, Columns 4 and 5)
iii. Codes quantifying alcohol use, comprised of:
   a. Drinking status (categorised as current drinker, ex drinker, non drinker; recorded in:
      - Read codes (**Supplementary Table 1**, Column 2)
      - Structured data, using data from the CPRD Additional Clinical Details file recorded under Entity Type 5, data field 1 (status) (Entity Type is the identifier that represents the structured data area in Vision where the data was entered)
   b. Level of drinking (non, light, moderate, heavy drinker; as recorded in Read codes (**Supplementary Table 1**, Column 3))
   c. Alcohol units consumed per week, using an existing (CALIBER: https://www.ucl.ac.uk/health-informatics/caliber) variable definition for 'number of alcohol unit consumption per week recorded in primary care (CPRD)',[1] using data from the CPRD Additional Clinical Details file recorded under Entity Type 5, data field 2 (units per week). We categorised units per week as: "None", "1-14, "15-42" or "43+" units per week. 14 units per week is the recommended limit for men and women according to current UK government guidelines.[2]
   d. AUDIT or FAST scores (AUDIT categorised according to WHO guidelines on risk level: 1-7 - low risk drinking; 8-15 - hazardous drinking; 16-19 - harmful drinking; 20-40 – possible dependence.[3]). Scores resulting from AUDIT/FAST screening (i.e. associated with a Read morbidity code indicating use of AUDIT/FAST), were obtained from the Additional Clinical Details file under Entity Type 172.

Mansfield K, *et al. BMJ Open* 2019; 9:e031537. doi: 10.1136/bmjopen-2019-031537

Supplementary Table 1. Read codes for alcohol data in CPRD (code lists also available for download: https://doi.org/10.17037/data.00001071).

CPRD medcode	Read code	Clinical term	1 Any alcohol-related code	2 Current drinking status	3 Codes indicating level of alcohol use	4 AUDIT screening	5 FAST screening
47555	90.95	cerebral degeneration due to alcoholism	1	0	0	0	0
27	136..00	alcohol consumption	1	0	0	0	0
12949	1361.00	Teetotaller	1	non	non	0	0
12970	1361.11	non drinker alcohol	1	non	non	0	0
4447	1361.12	non-drinker alcohol	1	non	non	0	0
12975	1362.00	trivial drinker - <1u/day	1	curr	L	0	0
385	1362.11	drinks rarely	1	curr	L	0	0
749	1362.12	drinks occasionally	1	curr	L	0	0
12972	1363.00	light drinker - 1-2u/day	1	curr	L	0	0
322	1364.00	moderate drinker - 3-6u/day	1	curr	M	0	0
1618	1365.00	heavy drinker - 7-9u/day	1	curr	H	0	0
12977	1366.00	very heavy drinker - >9u/day	1	curr	H	0	0
967	1367.00	stopped drinking alcohol	1	ex	non	0	0
12978	1368.00	alcohol consumption unknown	1	0	0	0	0
12976	1369.00	suspect alcohol abuse - denied	1	0	0	0	0
22933	136A.00	ex-trivial drinker (<1u/day)	1	0	0	0	0
102665	136a.00	increasing risk drinking	1	curr	H	0	0
26471	136B.00	ex-light drinker - (1-2u/day)	1	0	0	0	0
99877	136b.00	feels should cut down drinking	1	curr	H	0	0
19495	136C.00	ex-moderate drinker - (3-6u/d)	1	0	0	0	0
102448	136c.00	higher risk drinking	1	curr	H	0	0
19493	136D.00	ex-heavy drinker - (7-9u/day)	1	0	0	0	0
103230	136d.00	lower risk drinking	1	curr	M	0	0
12983	136E.00	ex-very heavy drinker-(>9u/d)	1	0	0	0	0
106560	136e.00	declines to state current alcohol consumption	1	0	0	0	0
12971	136F.00	spirit drinker	1	curr	0	0	0
2689	136G.00	beer drinker	1	curr	0	0	0
12968	136H.00	drinks beer and spirits	1	curr	0	0	0
12969	136I.00	drinks wine	1	curr	0	0	0
956	136J.00	social drinker	1	curr	M	0	0

Mansfield K, *et al. BMJ Open* 2019; 9:e031537. doi: 10.1136/bmjopen-2019-031537

CPRD medcode	Read code	Clinical term	1 Any alcohol-related code	2 Current drinking status	3 Codes indicating level of alcohol use	4 AUDIT screening	5 FAST screening
12982	136K.00	alcohol intake above recommended sensible limits	1	curr	H	0	0
26472	136L.00	alcohol intake within recommended sensible limits	1	curr	M	0	0
12979	136M.00	current non drinker	1	non	non	0	0
12980	136N.00	light drinker	1	curr	L	0	0
12985	136O.00	moderate drinker	1	curr	M	0	0
8999	136P.00	heavy drinker	1	curr	H	0	0
12984	136Q.00	very heavy drinker	1	curr	H	0	0
19401	136R.00	binge drinker	1	curr	H	0	0
19494	136S.00	hazardous alcohol use	1	curr	H	0	0
30695	136T.00	harmful alcohol use	1	curr	H	0	0
93415	136V.00	alcohol units per week	1	0	0	0	0
94670	136W.00	alcohol misuse	1	curr	H	0	0
97126	136X.00	alcohol units consumed on heaviest drinking day	1	0	0	0	0
101718	136Y.00	drinks in morning to get rid of hangover	1	curr	0	0	0
12981	136Z.00	alcohol consumption nos	1	0	0	0	0
18156	13Y8.00	alcoholics anonymous	1	0	0	0	0
84218	13ZY.00	disqualified from driving due to excess alcohol	1	0	0	0	0
8430	1462.00	h/o: alcoholism	1	0	0	0	0
38061	1B1c.00	alcohol induced hallucinations	1	curr	0	0	0
44783	1D19.00	pain in lymph nodes after alcohol consumption	1	0	0	0	0
24735	2577.00	o/e - breath - alcohol smell	1	curr	0	0	0
10161	2577.11	o/e - alcoholic breath	1	curr	0	0	0
69691	388j.00	cage questionnaire	1	0	0	0	0
70161	388j.00	cage questionnaire	1	0	0	0	0
93624	388u.00	fast alcohol screening test	1	0	0	0	1
96107	38D2.00	single alcohol screening questionnaire	1	0	0	0	0
94838	38D3.00	alcohol use disorders identification test	1	0	0	1	0
95744	38D4.00	alcohol use disorder identificatn test consumptn questionnre	1	0	0	1	0
97501	38D5.00	alcoh use disor id test piccinelli consumption questionnaire	1	0	0	1	0
100493	38Df.00	five-shot questionnaire on heavy drinking	1	0	0	0	0
102577	38Dz.00	severity of alcohol dependence questionnaire	1	0	0	0	0
102770	38Dz.11	sadq - severity of alcohol dependence questionnaire	1	0	0	0	0

Mansfield K, *et al. BMJ Open* 2019; 9:e031537. doi: 10.1136/bmjopen-2019-031537

CPRD medcode	Read code	Clinical term	1 Any alcohol-related code	2 Current drinking status	3 Codes indicating level of alcohol use	4 AUDIT screening	5 FAST screening
100989	4I91.11	breath alcohol level	1	0	0	0	0
17266	63C7.00	maternal alcohol abuse	1	0	0	0	0
12442	66e..00	alcohol disorder monitoring	1	curr	0	0	0
32964	66e0.00	alcohol abuse monitoring	1	0	0	0	0
11491	6792.00	health ed. - alcohol	1	0	0	0	0
35859	67A5.00	pregnancy alcohol advice	1	0	0	0	0
18711	67H0.00	lifestyle advice regarding alcohol	1	0	0	0	0
105144	67K6.00	cycle of change stage, alcohol	1	0	0	0	0
19489	6892.00	alcohol consumption screen	1	0	0	0	0
9264	68S..00	alcohol consumption screen	1	0	0	0	0
56410	7P22100	delivery of rehabilitation for alcohol addiction	1	0	0	0	0
2083	8BA8.00	alcohol detoxification	1	0	0	0	0
7692	8CAM.00	patient advised about alcohol	1	0	0	0	0
102564	8CAM000	advised to abstain from alcohol consumption	1	0	0	0	0
97309	8CAv.00	advised to contact primary care alcohol worker	1	curr	0	0	0
37264	8CE1.00	alcohol leaflet given	1	0	0	0	0
29691	8G32.00	aversion therapy - alcoholism	1	0	0	0	0
21650	8H35.00	admitted to alcohol detoxification centre	1	0	0	0	0
9849	8H7p.00	referral to community alcohol team	1	0	0	0	0
12554	8HHe.00	referral to community drug and alcohol team	1	0	0	0	0
94553	8HkG.00	referral to specialist alcohol treatment service	1	0	0	0	0
96993	8HkJ.00	referral to alcohol brief intervention service	1	0	0	0	0
94669	8IA7.00	alcohol consumption screening test declined	1	0	0	0	0
97261	8IAF.00	brief intervention for excessive alcohol consumptn declined	1	curr	0	0	0
97680	8IAJ.00	declined referral to specialist alcohol treatment service	1	curr	0	0	0
102247	8IAt.00	extended interven for excessive alcohol consumption declined	1	curr	0	0	0
103459	8IEA.00	referral to community alcohol team declined	1	curr	0	0	0
43813	9EQ..11	police:venesect-alcohol	1	0	0	0	0
102121	9EQ..12	police:venesect-alcohol	1	0	0	0	0
11740	9k1..00	alcohol misuse - enhanced services administration	1	curr	0	0	0
35330	9k11.00	alcohol consumption counselling	1	curr	0	0	0
63529	9k12.00	alcohol misuse - enhanced service completed	1	0	0	0	0

Mansfield K, *et al. BMJ Open* 2019; 9:e031537. doi: 10.1136/bmjopen-2019-031537

CPRD medcode	Read code	Clinical term	1 Any alcohol-related code	2 Current drinking status	3 Codes indicating level of alcohol use	4 AUDIT screening	5 FAST screening
48545	9k13.00	alcohol questionnaire completed	1	0	0	0	0
47123	9k14.00	alcohol counselling by other agencies	1	0	0	0	0
90714	9k15.00	alcohol screen - audit completed	1	0	0	1	0
94963	9k16.00	alcohol screen - fast alcohol screening test completed	1	0	0	0	1
95663	9k17.00	alcohol screen - audit c completed	1	0	0	1	0
94485	9k18.00	alcohol screen - audit pc completed	1	0	0	1	0
95944	9k19.00	alcohol assesment declined - enhanced services admin	1	0	0	0	0
96259	9k19.11	alcohol assessment declined	1	0	0	0	0
96053	9k1A.00	brief intervention for excessive alcohol consumptn completed	1	0	0	0	0
96054	9k1B.00	extended intervention for excessive alcohol consumptn complt	1	0	0	0	0
9489	9NN2.00	under care of community alcohol team	1	0	0	0	0
65754	C150500	alcohol-induced pseudo-cushing's syndrome	1	0	0	0	0
16237	E01..00	alcoholic psychoses	1	0	0	0	0
16225	E010.00	alcohol withdrawal delirium	1	curr	0	0	0
22277	E010.11	dts - delirium tremens	1	curr	0	0	0
1476	E010.12	Delirium tremens	1	curr	0	0	0
20762	E011.00	alcohol amnestic syndrome	1	0	0	0	0
4500	E011000	korsakov's alcoholic psychosis	1	0	0	0	0
11106	E011100	korsakov's alcoholic psychosis with peripheral neuritis	1	0	0	0	0
18636	E011200	wernicke-korsakov syndrome	1	0	0	0	0
41920	E011z00	alcohol amnestic syndrome nos	1	0	0	0	0
54505	E012.00	other alcoholic dementia	1	0	0	0	0
27342	E012.11	alcoholic dementia nos	1	0	0	0	0
37946	E012000	chronic alcoholic brain syndrome	1	0	0	0	0
25110	E013.00	alcohol withdrawal hallucinosis	1	curr	0	0	0
57939	E014.00	pathological alcohol intoxication	1	curr	0	0	0
20407	E014.11	drunkenness - pathological	1	curr	0	0	0
30404	E015.00	alcoholic paranoia	1	curr	0	0	0
33670	E01y.00	other alcoholic psychosis	1	0	0	0	0
2082	E01y000	alcohol withdrawal syndrome	1	curr	0	0	0
68111	E01yz00	other alcoholic psychosis nos	1	0	0	0	0
67651	E01z.00	alcoholic psychosis nos	1	0	0	0	0

Mansfield K, *et al. BMJ Open* 2019; 9:e031537. doi: 10.1136/bmjopen-2019-031537

CPRD medcode	Read code	Clinical term	1 Any alcohol-related code	2 Current drinking status	3 Codes indicating level of alcohol use	4 AUDIT screening	5 FAST screening
2084	E23..00	alcohol dependence syndrome	1	curr	0	0	0
2081	E23..11	alcoholism	1	curr	0	0	0
1399	E23..12	alcohol problem drinking	1	curr	H	0	0
5740	E230.00	acute alcoholic intoxication in alcoholism	1	curr	0	0	0
57714	E230.11	alcohol dependence with acute alcoholic intoxication	1	curr	0	0	0
40530	E230000	acute alcoholic intoxication, unspecified, in alcoholism	1	curr	0	0	0
56947	E230100	continuous acute alcoholic intoxication in alcoholism	1	curr	0	0	0
21624	E230200	episodic acute alcoholic intoxication in alcoholism	1	curr	0	0	0
59574	E230300	acute alcoholic intoxication in remission, in alcoholism	1	0	0	0	0
36296	E230z00	acute alcoholic intoxication in alcoholism nos	1	curr	0	0	0
31443	E231.00	chronic alcoholism	1	curr	0	0	0
37605	E231.11	Dipsomania	1	curr	0	0	0
43193	E231000	unspecified chronic alcoholism	1	curr	0	0	0
24064	E231100	continuous chronic alcoholism	1	curr	0	0	0
26106	E231200	episodic chronic alcoholism	1	curr	0	0	0
24485	E231300	chronic alcoholism in remission	1	0	0	0	0
33635	E231z00	chronic alcoholism nos	1	curr	0	0	0
6169	E23z.00	alcohol dependence syndrome nos	1	curr	0	0	0
7746	E250.00	nondependent alcohol abuse	1	curr	H	0	0
12271	E250.11	Drunkenness NOS	1	curr	0	0	0
27518	E250.12	hangover (alcohol)	1	curr	0	0	0
17777	E250.13	Inebriety NOS	1	curr	0	0	0
3782	E250.14	intoxication - alcohol	1	curr	0	0	0
669	E250000	nondependent alcohol abuse, unspecified	1	curr	H	0	0
23610	E250100	nondependent alcohol abuse, continuous	1	curr	H	0	0
12974	E250200	nondependent alcohol abuse, episodic	1	curr	H	0	0
31569	E250300	nondependent alcohol abuse in remission	1	0	0	0	0
28150	E250z00	nondependent alcohol abuse nos	1	curr	H	0	0
5611	Eu10.00	[x]mental and behavioural disorders due to use of alcohol	1	0	0	0	0
44299	Eu10000	[x]mental & behav dis due to use alcohol: acute intoxication	1	curr	0	0	0
9508	Eu10011	[x]acute alcoholic drunkenness	1	curr	0	0	0
21879	Eu10100	[x]mental and behav dis due to use of alcohol: harmful use	1	curr	0	0	0

Mansfield K, *et al. BMJ Open* 2019; 9:e031537. doi: 10.1136/bmjopen-2019-031537

CPRD medcode	Read code	Clinical term	1 Any alcohol-related code	2 Current drinking status	3 Codes indicating level of alcohol use	4 AUDIT screening	5 FAST screening
39327	Eu10200	[x]mental and behav dis due to use alcohol: dependence syndr	1	curr	0	0	0
28780	Eu10211	[x]alcohol addiction	1	curr	0	0	0
5758	Eu10212	[x]chronic alcoholism	1	curr	0	0	0
20514	Eu10300	[x]mental and behav dis due to use alcohol: withdrawal state	1	curr	0	0	0
64101	Eu10400	[X]Men & behav dis due alcohl: withdrawl state with delirium	1	curr	0	0	0
17259	Eu10411	[x]delirium tremens, alcohol induced	1	curr	0	0	0
12353	Eu10500	[x]mental & behav dis due to use alcohol: psychotic disorder	1	0	0	0	0
6467	Eu10511	[x]alcoholic hallucinosis	1	curr	0	0	0
65932	Eu10512	[x]alcoholic jealousy	1	curr	0	0	0
30162	Eu10513	[x]alcoholic paranoia	1	curr	0	0	0
17607	Eu10514	[x]alcoholic psychosis nos	1	0	0	0	0
39799	Eu10600	[x]mental and behav dis due to use alcohol: amnesic syndrome	1	0	0	0	0
11670	Eu10611	[x]korsakov's psychosis, alcohol induced	1	0	0	0	0
62000	Eu10700	[X]Men & behav dis due alcoh: resid & late-onset psychot dis	1	0	0	0	0
26323	Eu10711	[x]alcoholic dementia nos	1	0	0	0	0
37691	Eu10712	[x]chronic alcoholic brain syndrome	1	0	0	0	0
32927	Eu10800	[x]alcohol withdrawal-induced seizure	1	curr	0	0	0
45169	Eu10y00	[x]men & behav dis due to use alcohol: oth men & behav dis	1	0	0	0	0
64389	Eu10z00	[x]ment & behav dis due use alcohol: unsp ment & behav dis	1	0	0	0	0
36748	F11x011	alcoholic encephalopathy	1	0	0	0	0
33839	F144000	cerebellar ataxia due to alcoholism	1	0	0	0	0
30604	F25B.00	alcohol-induced epilepsy	1	0	0	0	0
2925	F375.00	alcoholic polyneuropathy	1	0	0	0	0
31742	F394100	alcoholic myopathy	1	0	0	0	0
4915	G555.00	alcoholic cardiomyopathy	1	0	0	0	0
8363	G852300	oesophageal varices in alcoholic cirrhosis of the liver	1	0	0	0	0
4506	J153.00	alcoholic gastritis	1	0	0	0	0
10691	J610.00	alcoholic fatty liver	1	0	0	0	0
3216	J611.00	acute alcoholic hepatitis	1	0	0	0	0
4743	J612.00	alcoholic cirrhosis of liver	1	0	0	0	0
21713	J612000	alcoholic fibrosis and sclerosis of liver	1	0	0	0	0
7885	J613.00	alcoholic liver damage unspecified	1	0	0	0	0

Mansfield K, *et al. BMJ Open* 2019; 9:e031537. doi: 10.1136/bmjopen-2019-031537

CPRD medcode	Read code	Clinical term	1 Any alcohol-related code	2 Current drinking status	3 Codes indicating level of alcohol use	4 AUDIT screening	5 FAST screening
17330	J613000	alcoholic hepatic failure	1	0	0	0	0
7943	J617.00	alcoholic hepatitis	1	0	0	0	0
7602	J617000	chronic alcoholic hepatitis	1	0	0	0	0
104611	J670800	alcohol-induced acute pancreatitis	1	0	0	0	0
24984	J671000	alcohol-induced chronic pancreatitis	1	0	0	0	0
66019	L254.11	suspect fetal damage from maternal alcohol	1	0	0	0	0
27670	L255300	maternal care for (suspected) damage to fetus from alcohol	1	0	0	0	0
72757	Q007100	fetus/neonate affected by placental/breast transfer alcohol	1	0	0	0	0
97916	Q007111	fetal alcohol syndrome	1	0	0	0	0
9169	R103.00	[d]alcohol blood level excessive	1	0	0	0	0
36687	SLH3.00	alcohol deterrent poisoning	1	0	0	0	0
19217	SM0..00	alcohol causing toxic effect	1	0	0	0	0
8984	SM00.00	ethyl alcohol causing toxic effect	1	0	0	0	0
48514	SM00100	denatured alcohol causing toxic effect	1	0	0	0	0
55536	SM00z00	ethyl alcohol causing toxic effect nos	1	0	0	0	0
88997	SM01.00	methyl alcohol causing toxic effect	1	0	0	0	0
11263	SM01100	wood alcohol causing toxic effect	1	0	0	0	0
106018	SM02.00	isopropyl alcohol causing toxic effect	1	0	0	0	0
102086	SM02200	rubbing alcohol causing toxic effect	1	0	0	0	0
64396	SM03000	amyl alcohol causing toxic effect	1	0	0	0	0
102321	SM03100	butyl alcohol causing toxic effect	1	0	0	0	0
101543	SM0y.00	other alcohol causing toxic effect	1	0	0	0	0
36499	SM0z.00	alcohol causing toxic effect nos	1	0	0	0	0
95410	SyuG000	[x]toxic effect of other alcohols	1	0	0	0	0
18252	T90..00	accidental poisoning by alcohol, nec	1	0	0	0	0
40541	T900.00	accidental poisoning by alcoholic beverages	1	0	0	0	0
15503	T901.00	accidental poisoning by other ethyl alcohol and its products	1	0	0	0	0
101426	T901z00	accidental poisoning by ethyl alcohol nos	1	0	0	0	0
37174	T902.00	accidental poisoning by methyl alcohol	1	0	0	0	0
60752	T903300	accidental poisoning by secondary propyl alcohol	1	0	0	0	0
53139	T90y.00	accidental poisoning by other alcohols	1	0	0	0	0
55415	T90z.00	accidental poisoning by alcohol nos	1	0	0	0	0

Mansfield K, *et al. BMJ Open* 2019; 9:e031537. doi: 10.1136/bmjopen-2019-031537

CPRD medcode	Read code	Clinical term	1 Any alcohol-related code	2 Current drinking status	3 Codes indicating level of alcohol use	4 AUDIT screening	5 FAST screening
21412	TJH3.00	adverse reaction to alcohol deterrents	1	0	0	0	0
31605	U1A9.00	[x]accident poisoning/exposure to alcohol	1	0	0	0	0
63306	U1A9000	[x]accident poison/exposure to alcohol at home	1	0	0	0	0
103069	U1A9200	[x]acc poison/expos alcohol school/pub admin area	1	0	0	0	0
104702	U1A9300	[x]accid pois/expos alcohol in sport/athletic area	1	0	0	0	0
63876	U1A9400	[x]accid poison/expos alcohol in street/highway	1	0	0	0	0
92908	U1A9500	[x]accid poison/expos alcohol trade/service area	1	0	0	0	0
104734	U1A9y00	[x]accid pois/expos to alcohol other spec place	1	0	0	0	0
61187	U1A9z00	[x]accid poison/expos to alcohol unspecif place	1	0	0	0	0
10463	U209.00	[x]intent self poison/exposure to alcohol	1	0	0	0	0
41638	U209000	[x]int self poison/exposure to alcohol at home	1	0	0	0	0
97085	U209y00	[x]int self poison alcohol other spec place	1	0	0	0	0
59414	U209z00	[x]intent self poison alcohol unspecif place	1	0	0	0	0
30036	U409.00	[x]poisoning/exposure, ? intent, to alcohol	1	0	0	0	0
68159	U409000	[x]poison/exposure ?intent, to alcohol at home	1	0	0	0	0
69407	U409200	[x]pois/exp ?intent alcohol school/pub admin area	1	0	0	0	0
96219	U409400	[x]pois/expos ?intent alcohol in street/highway	1	0	0	0	0
61190	U409z00	[x]pois/expos ?intent to alcohol unspecif place	1	0	0	0	0
73876	U60H300	[x]alcohol deterrents caus adverse effects in therapeut use	1	0	0	0	0
48241	U60H311	[x] adverse reaction to alcohol deterrents	1	0	0	0	0
44019	U80..00	[x]evidence of alcohl involv determin by blood alcohl level	1	0	0	0	0
12950	U800.00	[x]eviden of alcohl involv blood alcohl level <20 mg/100 ml	1	0	0	0	0
57202	U801.00	[x]eviden of alcohl involv blood alcohl level 20-39mg/100ml	1	0	0	0	0
59079	U802.00	[x]eviden of alcohl involv blood alcohl level 40-59mg/100ml	1	0	0	0	0
57242	U803.00	[x]eviden of alcohl involv blood alcohl level 60-79mg/100ml	1	0	0	0	0
53428	U804.00	[x]eviden of alcohl involv blood alcohl level 80-99mg/100ml	1	0	0	0	0
54504	U805.00	[x]eviden of alcoh involv blood alcoh level 100-119mg/100ml	1	0	0	0	0
32454	U806.00	[x]eviden of alcoh involv blood alcoh level 120-199mg/100ml	1	0	0	0	0
47907	U807.00	[x]eviden of alcoh involv blood alcoh level 200-239mg/100ml	1	0	0	0	0
39726	U808.00	[x]eviden alcoh involv blood alcoh level 240mg/100ml or more	1	0	0	0	0
103698	U80z.00	[x]evid alcoh invol detrm by pres alcoh in bld levl not spec	1	0	0	0	0
23978	U81..00	[x]evid of alcohol involv determind by level of intoxication	1	0	0	0	0

Mansfield K, *et al. BMJ Open* 2019; 9:e031537. doi: 10.1136/bmjopen-2019-031537

CPRD medcode	Read code	Clinical term	1 Any alcohol-related code	2 Current drinking status	3 Codes indicating level of alcohol use	4 AUDIT screening	5 FAST screening
105273	U810.00	[x]evid alcoh invol determ by levl of intox mild alcoh intox	1	0	0	0	0
73480	U811.00	[x]evid alcoh invol determ by level of intox mod alcoh intox	1	0	0	0	0
44686	U812.00	[x]evid alcoh invol determ by level of intox sev alcoh intox	1	0	0	0	0
39738	U813.00	[x]evid alcoh invl determ by levl intox very sev alcoh intox	1	0	0	0	0
41983	Z191.00	alcohol detoxification	1	0	0	0	0
46677	Z191100	alcohol withdrawal regime	1	0	0	0	0
61383	Z191200	planned reduction of alcohol consumption	1	curr	0	0	0
95181	Z191211	alcohol reduction programme	1	curr	0	0	0
64409	Z191400	self-monitoring of alcohol intake	1	0	0	0	0
30460	Z4B1.00	alcoholism counselling	1	curr	0	0	0
21829	Z786200	drinking practice	1	0	0	0	0
95650	ZC22100	advice to change drink intake	1	curr	0	0	0
97163	ZC22200	advice to change alcoholic drink intake	1	curr	0	0	0
54209	ZC2H.00	advice to change alcohol intake	1	curr	0	0	0
11140	ZG23100	advice on alcohol consumption	1	0	0	0	0
50507	ZR1E.00	alcohol dependence scale	1	0	0	0	0
40602	ZR1E.11	ads - alcohol dependence scale	1	0	0	0	0
32850	ZR1F.00	alcohol use disorders identification test	1	0	0	1	0
59873	ZR1F.11	audit - alcohol use disorders identification test	1	0	0	1	0
65980	ZR1G.00	alcohol use inventory	1	0	0	0	0
45557	ZR31.00	cage questionnaire	1	0	0	0	0
61750	ZR3f.00	comprehensive drinker profile	1	0	0	0	0
63457	ZR3f.11	cdp - comprehensive drinker profile	1	0	0	0	0
56441	ZRa1.00	michigan alcoholism screening test	1	0	0	0	0
69331	ZRa1.11	mast - michigan alcoholism screening test	1	0	0	0	0
99985	ZRa1100	brief michigan alcoholism screening test	1	0	0	0	0
41251	ZRa1111	bmast - brief michigan alcoholism screening test	1	0	0	0	0
70939	ZRaU.00	munich alcoholism test	1	0	0	0	0
22707	ZRBJ.00	drinking problem scale	1	0	0	0	0
46848	ZRBJ.11	dps - drinking problem scale	1	0	0	0	0
42305	ZRk6.00	severity of alcohol dependence questionnaire	1	0	0	0	0
66831	ZRk6.11	sadq - severity of alcohol dependence questionnaire	1	0	0	0	0

Mansfield K, *et al. BMJ Open* 2019; 9:e031537. doi: 10.1136/bmjopen-2019-031537

CPRD medcode	Read code	Clinical term	1 Any alcohol-related code	2 Current drinking status	3 Codes indicating level of alcohol use	4 AUDIT screening	5 FAST screening
62300	ZRk9.00	short alcohol dependence data	1	0	0	0	0
62299	ZRk9.11	sadd - short alcohol dependence data	1	0	0	0	0
10658	ZRLfD12	honos item 3 - alcohol/drug problem	1	0	0	0	0
66552	ZRR..00	inventory of drinking situations	1	0	0	0	0
59776	ZRVK.00	last six months of drinking questionnaire	1	0	0	0	0
7123	ZV11300	[v]personal history of alcoholism	1	0	0	0	0
16587	ZV11311	[v]problems related to lifestyle alcohol use	1	curr	H	0	0
7545	ZV4KC00	[v] alcohol use	1	0	0	0	0
8388	ZV57A00	[v]alcohol rehabilitation	1	0	0	0	0
8030	ZV6D600	[v]alcohol abuse counselling and surveillance	1	curr	0	0	0
61583	ZV70411	[v]medicolegal blood alcohol test	1	0	0	0	0
17149	ZV70L00	[v]blood-alcohol and blood-drug test	1	0	0	0	0
10458	ZV79100	[v]screening for alcoholism	1	0	0	0	0

Mansfield K, *et al. BMJ Open* 2019; 9:e031537. doi: 10.1136/bmjopen-2019-031537

Supplementary Table 2. Internal validity comparing alcohol consumption recorded up to 30 days before or after an AUDIT score, AUDIT-C score or measure of units per week.

	N	(%)	N patients with AUDIT score on the same date as record n	(%)	AUDIT score median	(IQR)	N patients with AUDIT C score on the same date as record n	(%)	AUDIT C score median	(IQR)	N patients with units per week recorded on same date as record n	(%)	Units per week median	(IQR)
**Current drinking status***														
Non	174,970	(20.3)	3053	(1.7)	0	(0-0)	1096	(0.6)	0	(0-0)	882	(0.5)	0	(0-1)
Ex	31,526	(3.7)	326	(1)	0	(0-0)	43	(0.1)	0	(0-0)	999	(3.2)	0	(0-1)
Current	655,834	(76.1)	24402	(3.7)	3	(1-5)	4707	(0.7)	3	(2-5)	333,402	(50.8)	6	(2-14)
**Current drinking level (Read codes)**														
Non-drinker	222835	(39.9)	3437	(1.5)	0	(0-0)	1145	(0.5)	0	(0-0)	2,715	(1.2)	0	(0-2)
Light drinker	255,919	(45.8)	3,401	(1.3)	2	(1-3)	1,131	(0.4)	2	(1-3)	19,777	(7.7)	3	(1-8)
Moderate drinker	52,070	(9.3)	1,205	(2.3)	1	(0-4)	236	(0.5)	3	(2-4)	13,024	(25)	6	(2-14)
Heavy drinker	27,923	(5)	2,906	(10.4)	6	(5-9)	127	(0.5)	8	(6-9)	10,572	(37.9)	18	(10-30)
**Units per week**														
0	38,929	(9)	1,919	(4.9)	0	(0-0)	159	(0.4)	0	(0-1)				
1-14	298,441	(69.2)	11,920	(4)	3	(2-4)	2,273	(0.8)	3	(2-4)	n/a		n/a	
15-42	80,449	(18.6)	2,983	(3.7)	6	(4-8)	412	(0.5)	5	(4-7)				
43+	13,575	(3.1)	248	(1.8)	10	(8-12)	19	(0.1)	8	(5-10)				
**AUDIT C score category**														
0-4; Low risk	16,206	(76.8)	4,006	(24.7)	2	(0-3)	n/a		n/a		2,120	(13.1)	4	(2-8)
5-12; High risk	4,893	(23.2)	1,160	(23.7)	6	(5-7)					901	(18.4)	12	(7-20)
**AUDIT score category**														
0-7; Alcohol Education	71,401	(92.5)					4,899	(6.9)	2	(1-4)	15,852	(22.2)	4	(2-10)
8-15; Simple Advice	5,405	(7)					242	(4.5)	9	(8-10)	1,605	(29.7)	20	(14-30)
16-19; Simple Advice plus Brief Counseling and Continued Monitoring	206	(0.3)	n/a		n/a		0	(0)		(-)	49	(23.8)	24	(16-48)
20-40; Referral to Specialist for Diagnostic Evaluation and Treatment	200	(0.3)					0	(0)		(-)	30	(15)	47	(20-80)

*based on recorded status in Read codes and Additional details (not including derived status based on units consumed or AUDIT score)

Mansfield K, *et al. BMJ Open* 2019; 9:e031537. doi: 10.1136/bmjopen-2019-031537

References

1. Denaxas, S.C., et al., *Data resource profile: cardiovascular disease research using linked bespoke studies and electronic health records (CALIBER).* Int J Epidemiol, 2012. **41**(6): p. 1625-38.

2. Department of Health, *UK Chief Medical Officers' alcohol guidelines review: Summary of the proposed new guidelines.* 2016.

3. Babor, T.F., et al. *The Alcohol Use Disorders Identification Test: Guidelines for Use in Primary Care*. 2001; Available from: http://www.talkingalcohol.com/files/pdfs/WHO_audit.pdf.

Mansfield K, *et al. BMJ Open* 2019; 9:e031537. doi: 10.1136/bmjopen-2019-031537
