# Supplementary material for: Completeness and validity of alcohol recording in general practice within the UK: a cross-sectional study
Source: BMJ Open. 2019 Nov 26;9(11):e031537. doi: 10.1136/bmjopen-2019-031537 (PMC6887039; doi:10.1136/bmjopen-2019-031537)
Supplement: Author's manuscript [file bmjopen-2019-031537.draft_revisions.pdf]

BMJ Open is committed to open peer review. As part of this commitment we make the peer review history of every article we publish publicly available.

When an article is published we post the peer reviewers' comments and the authors' responses online. We also post the versions of the paper that were used during peer review. These are the versions that the peer review comments apply to.

The versions of the paper that follow are the versions that were submitted during the peer review process. They are not the versions of record or the final published versions. They should not be cited or distributed as the published version of this manuscript.

BMJ Open is an open access journal and the full, final, typeset and author-corrected version of record of the manuscript is available on our site with no access controls, subscription charges or pay-per-view fees (http://bmjopen.bmj.com).

If you have any questions on BMJ Open's open peer review process please email
info.bmjopen@bmj.com

BMJ Open

Completeness and validity of alcohol recording in general practice within the UK: a cross-sectional study

Journal:	*BMJ Open*
Manuscript ID	bmjopen-2019-031537
Article Type:	Research
Date Submitted by the Author:	08-May-2019

Complete List of Authors:	Mansfield, Kathryn; London School of Hygiene and Tropical Medicine, Non-communicable Disease Epidemiology<br>Crellin, Elizabeth; Clinical Practice Research Datalink<br>Denholm, Rachel; University of Bristol, Centre for Academic Primary Care<br>Quint, Jennifer; Imperial College London, Respiratory Epidemiology, Occupational Medicine and Public Health<br>Smeeth, Liam; London School of Hygiene and Tropical Medicine, Epidemiology and Population Health<br>Cook, Sarah; London School of Hygiene & Tropical Medicine,<br>Herrett, Emily; London School of Hygiene and Tropical Medicine, Non communicable disease epidemiology

Keywords:	alcohol, general practice, recording, validity, screening

SCHOLARONE™
Manuscripts

Completeness and validity of alcohol recording in general practice within the UK: a cross-sectional study

Kathryn Mansfield[1*], Elizabeth Crellin[2*], Rachel Denholm[3], Jennifer Quint[4], Liam Smeeth[1], Sarah Cook[1], Emily Herrett[1]

* Joint first authors.

1 - Department of Non-Communicable Diseases Epidemiology
London School of Hygiene and Tropical Medicine
London, UK

2 – Clinical Practice Research Datalink
3 – Centre for Academic Primary Care, Bristol Medical School,
University of Bristol,
Bristol, UK
4 – Imperial College London

Correspondence:
Dr Emily Herrett
Department of Non-Communicable Diseases Epidemiology
London School of Hygiene and Tropical Medicine
London UK
emily.herrett@lshtm.ac.uk

Word count 4182

For peer review only - http://bmjopen.bmj.com/site/about/guidelines.xhtml

Abstract

Background: Since 2010 the National Institute for Health and Care Excellence has recommended screening adults for excessive alcohol consumption to try and help prevent alcohol-use disorders. Little is known about the extent to which these recommendations are followed, and the resulting completeness and validity of alcohol-related data recording in primary care.

Objective: To investigate the completeness and accuracy of recording of alcohol use within primary care records in the UK.

Design and setting: Cross-sectional study in the Clinical Practice Research Datalink

Participants: We included all adult patients registered on 1st January 2018 with ≥1 year of follow-up.

Primary and secondary outcome measures: We calculated prevalence of alcohol consumption recording overall and within patient groups. We then validated alcohol consumption data against recommended screening tools (AUDIT) as the gold standard. We also calculated how prevalence of alcohol recording changed over the preceding decade.

Results: In 2018, among 1.8 million registered adult patients, just over half (51.9%) had a record for a code related to alcohol in the previous five years. Recording of alcohol consumption was more common among women, older people, ex-smokers, and those from more deprived areas, who were overweight/obese, or with comorbidities. A quarter of patients had units per week recorded in the last five years, but <10% had an AUDIT or FAST alcohol screening test score. The recorded alcohol measures corresponded to results from gold standard AUDIT scores. The distribution of consumption among current drinkers was similar to the Health Survey for England.

Conclusions: Half of adults in UK primary care have no recorded alcohol consumption data. When consumption is recorded, we have demonstrated internal and external validity of the data, suggesting greater recording may help identify opportunities for interventions to reduce harms.

For peer review only - http://bmjopen.bmj.com/site/about/guidelines.xhtml

Strengths and limitations of the study

- This study investigated alcohol recording across nearly 1.8 million UK adults in primary care, providing the power to understand in detail how alcohol use is recorded in general practice.
- We demonstrated the validity of alcohol recording internally within general practice records, and externally compared to the Health Survey for England.
- Though we demonstrated levels of alcohol recording and screening, it does not mean that GPs have acted upon the screening result, or that they have screened at an appropriate time.

For peer review only

For peer review only - http://bmjopen.bmj.com/site/about/guidelines.xhtml

Introduction

Hazardous alcohol use is a key behavioural risk factor affecting health. It is estimated that over ten million people in the UK are drinking at levels that risk their health, with a resulting cost to the NHS of over £3 billion per year.[1] In the age group 15-49 years, alcohol is the leading risk factor for ill health, early death and disability.[2]

Identification of high-risk patients can usefully be undertaken in primary care, where appropriate interventions can be promoted. The Alcohol Use Disorders Identification Test (AUDIT) [3] is the main tool for alcohol screening recommended by National Institute for Health and Care Excellence (NICE), though shortened versions (such as AUDIT-C and FAST) are also considered acceptable.[4] National Institute for Health and Care Excellence (NICE) guidelines published in 2010 recommend that opportunistic screening of the general population should take place during new patient registrations, and when screening or managing other conditions, promoting sexual health, during antenatal appointments and treating minor injuries. In addition, guidelines recommend screening 'high risk' groups, including those with relevant physical conditions (such as hypertension) and relevant mental health problems (such as depression).[4] Additionally, the NHS health check, which is offered to all people in the UK aged 40-74 years every five years, includes the AUDIT questions.[5]

Khadjesari et al (2013) showed relatively high levels of alcohol recording (76%) among new registrants in primary care between 2007 and 2009, although the use of validated screening tools was low (9%). They did not report levels for the whole registered patient population, which includes those who are less mobile and not subject to new patient registrations, nor specifically among patients with conditions that may be associated with, or exacerbated by, high-risk drinking. Poor recording of alcohol use has two major impacts: first and most importantly, patients may not be receiving appropriate interventions; and secondly, for

For peer review only - http://bmjopen.bmj.com/site/about/guidelines.xhtml

researchers, poor recording may prohibit reliable studies of the impact of alcohol on health using these data. Indeed, some recent studies using alcohol data in a large UK-based primary care electronic health record database (Clinical Practice Research Datalink [CPRD]) have indicated that bias may have arisen due to poor recording of alcohol data.[6, 7] For researchers using the data it is important to know not just whether screening occurs but if there are particular sub-groups more likely to be screened and to what extent alcohol codes used are meaningful reflections of actual alcohol use.

Using primary care data from the CPRD, this study assessed the utility of recorded alcohol data in primary care by assessing completeness of recording of alcohol status at different levels and by various patient level factors,and comparing measures of alcohol recorded in the CPRD internally and to external sources. Our study also offers insight into the completeness of alcohol data in primary care since the introduction of 2010 NICE guidance, including specifically among those identified as being at higher risk according to NICE recommendations, and provides guidance for future studies related to alcohol use using primary care data.

For peer review only - http://bmjopen.bmj.com/site/about/guidelines.xhtml

Methods

Study design and setting

We undertook a cross-sectional study with data from the Clinical Practice Research Datalink GOLD database, an ongoing primary care database containing the anonymised medical records from general practitioners in the UK using the Vision software to capture health record data with coverage of 15.4 million patients. Data from CPRD includes diagnoses, tests, clinical measurements, prescriptions and specialist referrals. CPRD uses Read morbidity coding to summarise each patient encounter with a code (or codes) that correspond to a standard set of clinical terminology. Roughly half of all practices in the CPRD have consented to data linkage and therefore deprivation data (based on quintiles of the Indices of Multiple Deprivation (IMD)) is available for these practices. The study included all patients in CPRD GOLD who were 18 years or over, alive and registered, for at least one year, with a CPRD GOLD practice on 1st January 2018.

Factors associated with alcohol recording

We investigated a range of potential factors that may be associated with alcohol-use recording in primary care: age, sex, deprivation, geographic region, comorbidities related to alcohol use (liver disease, hypertension, depression and anxiety) or those where health function monitoring is likely to be more frequent/complete (diabetes mellitus), time since registration at the current practice (1-5 years vs ≥5 years), body mass index (BMI), smoking status, and ethnicity.

We identified liver disease, hypertension and diabetes mellitus based on presence of a recorded diagnosis at any time prior to the study date. We identified depression and anxiety based on the presence of a diagnosis at any time within the five years prior to the study date (1st January 2018). We used existing morbidity code lists and algorithms to define smoking status, BMI and ethnicity.[8, 9]

For peer review only - http://bmjopen.bmj.com/site/about/guidelines.xhtml

Outcomes

Our main outcomes were the prevalence of alcohol-use recording in the five years prior to the study date, 1st January 2018. Prevalence of alcohol recording included any record of alcohol use or its effects, and we also broke it down into the following categories:

i. Any code suggesting that alcohol was discussed in the consultation.
ii. Codes indicating AUDIT or FAST screening
iii. Codes quantifying alcohol use, comprised of:
    a. drinking status (current drinker, ex, non; either as recorded in Read codes, or in structured data),
    b. level of drinking (non, light, moderate, heavy drinker; as recorded in Read codes),
    c. units per week,
    d. AUDIT or FAST scores (AUDIT categorised according to WHO guidelines on risk level: 1-7 - low risk drinking; 8-15 - hazardous drinking; 16-19 - harmful drinking; 20-40 – possible dependence.[10]).

We have made all morbidity code lists used in this study available to download (https://doi.org/10.17037/data.00001071). We have also provided further detail regarding variable definitions in the appendices.

We considered the record of a score from an alcohol-screening test using a validated tool (AUDIT or FAST) as the gold standard for alcohol recording. The AUDIT score is a 10-item instrument with a maximum score of 40 designed specifically for screening for hazardous or harmful alcohol use in primary care.[11, 12] A score of eight or more on the AUDIT tool is associated with harmful or hazardous drinking, although a slightly lower threshold can be used in women. The AUDIT tool can be abbreviated in time-limited settings to the first three questions (dealing with alcohol consumption only) (AUDIT-C) to give a score out of 12 or a four-item screening tool (FAST), which give a score out 16. These abbreviated tools have

For peer review only - http://bmjopen.bmj.com/site/about/guidelines.xhtml

lower thresholds to indicate harmful or hazardous drinking than the standard AUDIT tool: AUDIT-C – three for women and four for men; and FAST – three.[13-15] Due to differences in scoring systems and different threshold scores to identify problem dinking between the AUDIT, AUDIT-C and FAST tools, we restricted our internal validation to those with the full AUDIT score.

Statistical analysis

Completeness of alcohol recording

To investigate the completeness of alcohol recording, we estimated the proportion of patients with a record for one or more morbidity codes relating to alcohol in the categories outlined above (i.e. i. Any code suggesting that alcohol was discussed in the consultation; ii. Codes indicating AUDIT or FAST screening; or iii. Codes quantifying alcohol use) in the five years prior to 1st January 2018 (each patient could have codes in more than one category). We then described alcohol recording prevalence in strata of individual characteristics, including age, sex, deprivation, geographic region, comorbidities, smoking status, BMI and ethnicity. We also described the frequency of consultations with a record of an alcohol code in the five years before the study date, and the number of days from a patient's registration to their first record of an alcohol use code.

Internal validity

We validated codes quantifying the level of alcohol use (i.e. current/ex/non; light/moderate/heavy; units/week [Category iii above]) against an AUDIT score from the *same date* and the *same patient*. We calculated median AUDIT scores for each category of level of alcohol use recording: (a) drinking status, (b) drinking level and (c) units per week. Similarly, we also validated codes indicating drinking status, drinking level and AUDIT scores against the number of units per week. For each variable, we only included a patient's most recent record (to the study date).

For peer review only - http://bmjopen.bmj.com/site/about/guidelines.xhtml

For peer review only

External validity

We took the most recent alcohol record indicating alcohol use status (classified as current or non-/ex-drinkers based on morbidity coding) or units per week for each patient. We described current alcohol-use status separately for men and women from CPRD, and compared the results to the Health Survey for England 2016.[16] Then, for CPRD patients with a recorded 'current' drinking status, we grouped the recorded number of units consumed per week into the following categories: men ≤14 (lower risk), 15-50 (increased risk), >50 (higher risk); women ≤14 (lower risk), 15-35 (increased risk), >35 (higher risk), and again compared these data to the Health Survey for England data 2016.[16]

Time trends in alcohol recording

To understand how alcohol recording has been changing over time, we carried out a cross sectional study on the 1st January each year between 2009 and 2018. We estimated the number of patients who would be eligible for the study, using the same criteria as above (registered for one year at a CPRD practice, and aged 18 or over on the study date), and calculated the proportion of patients with a record of alcohol consumption, and level of drinking over the past five years.

Sensitivity analyses

1. In the main analysis, we restricted the validation of alcohol codes to cases where patients had an AUDIT score recorded on the same date as another alcohol code of interest. In sensitivity analyses, we included cases where any AUDIT score was recorded up to 30 days before or after the code of interest.
2. In the main analysis, we recorded liver disease, hypertension and diabetes mellitus based on presence of a diagnosis at any time prior to the study date. We tested this

For peer review only - http://bmjopen.bmj.com/site/about/guidelines.xhtml

definition by repeating the analysis limiting to diagnoses of these comorbidities within five years prior to the study date.

3. In the main analysis, we recorded depression and anxiety based on presence of a diagnosis within five years prior to the study date. We tested this definition by repeating the analysis limiting to diagnoses of these comorbidities within 12 months prior to the study date.

Data were analysed using Stata v14 (StataCorp, Texas, USA).

For peer review only

For peer review only - http://bmjopen.bmj.com/site/about/guidelines.xhtml

Results

There were 1,768,651 adults aged 18 years or over, alive and registered, for at least one year, with a CPRD practice on 1st January 2018, and therefore included in the study; 49.4% were male, and their mean age was 49.7 years. The mean duration of registration prior to the study date was 18.7 years (standard deviation 14.5 years). One fifth had hypertension, 2.0% had liver disease, 6.8% had diabetes, 6.7% had depression and 4.6% had anxiety.

Prevalence

Overall, 918,254 (51.9%) patients had a record indicating that alcohol was discussed within the last five years. This included diagnoses of alcohol use disorders, their management, harm due to alcohol consumption, in addition to the consumption of alcohol.

Drinking status

Drinking status (current, ex-, non-drinker) was recorded in 862,330 (48.8%) of patients, though this was not consistent across patient groups. Recording of drinking status was higher in women compared to men (52.7% in women vs 44.7% in men), increased with age (30.6% in 18-24 years, 62.4% in 75+ years) and was more common among recently registered patients (in the last one to five years) than in those registered for five or more years (73.6% vs 43.0%, respectively). There were also differences in recording between geographic region (higher recording in the North of England, lower recording in Northern Ireland and Wales), by deprivation (recording was more common in deprived areas) and by ethnicity (highest for white patients). Patients who had missing smoking or BMI data had particularly low levels of alcohol status recorded, but even in those with smoking status recorded, drinking status was only recorded in approximately 50%-60% of patients. If smoking and BMI were recorded there were differences in the recording of alcohol status at different levels of smoking/BMI: for example, ex and current smokers were more likely to have alcohol status recorded than non-smokers, as were patients who were overweight.

For peer review only - http://bmjopen.bmj.com/site/about/guidelines.xhtml

Patients were also more likely to have alcohol status recorded with each of the morbidities investigated (liver disease, hypertension, diabetes, depression and anxiety). Patients with diabetes were the most likely to have alcohol levels recorded (83% had an alcohol record) (**Table 1**).

Level of consumption

Overall, 862,191 (48.7%) of adult patients had some level of alcohol consumption (including non-drinkers) recorded in the previous five years, identified through recording of Read codes (heavy, moderate, light or non-drinker), units per week or formal alcohol screening score using AUDIT or FAST.

Level of alcohol consumption data were recorded using Read coding for 31.6% of patients, in units per week for 24.4% of patients, and via a record of formal alcohol screening for 11.0% of patients with a Read code indicating that they had completed an AUDIT or FAST questionnaire, but only 8.2% of patients with a score available.

Recording of level of alcohol consumption by patient and practice characteristics are shown in **Table 1**. Patients with diabetes had the highest levels of alcohol consumption recording, but only 39.5% of them had a recording of units per week, and only 9.8% had a recorded AUDIT or FAST score.

Among patients with a recorded drinking status, 20.3% were non-drinkers, 3.7% ex-drinkers, and 76.0% current drinkers. **Table 2** describes levels of drinking amongst patients for whom it was recorded.

If we use all data (from all levels of alcohol consumption categories, i.e. coded heavy/moderate/light/non-drinker, recorded units per week or formal alcohol screening

For peer review only - http://bmjopen.bmj.com/site/about/guidelines.xhtml

score) to derive drinking status based on recorded status, units per week, and AUDIT scores, an additional 35,099 patients (2.0% of eligible patients) have a drinking status. This derived status shows that 20.8% are non-drinkers, 3.7% ex-drinkers and 76.1% are current drinkers.

Internal validity

In our study population, there were 144,739 (8.2%) patients with at least one AUDIT score record available, and 431,394 with units-per-week records. **Table 2** describes the cross validation between alcohol measures and AUDIT scores and units per week.

Drinking status: Current drinking (based on Read coding) was associated with a median AUDIT score of three (IQR 1-5), which is under the standard threshold defining risky drinking, and none/ex drinkers had a median AUDIT score of zero. On average, current drinkers drank six units per week (IQR 2-14).

Drinking level: light and moderate drinkers had very low scores, and heavy drinkers had a median score of six (IQR 4-9), which indicates alcohol education. Light drinkers drank three units per week (IQR 1-8), moderate drinkers six per week (IQR 2-14), and heavy drinkers 18 per week (IQR 9-30).

Units per week: increased with increasing AUDIT scores, but even the highest category of units per week (43+) only had a median AUDIT score of 11 (IQR 8-12), which indicates simple alcohol advice.

AUDIT scores: the majority (93%) of patients had scores in the lowest category (alcohol education). Patients with higher AUDIT scores consumed more units per week; for example, those in the lowest AUDIT score category (0-7 Alcohol education) had a median units per week of four (IQR 2-10) compared to a median of 60 units per week (IQR 25-79) in the highest AUDIT score category (score 20-40, indicating referral to specialist for diagnostic evaluation and treatment).

For peer review only - http://bmjopen.bmj.com/site/about/guidelines.xhtml

External validity

For men and women, the proportions of current and non-drinkers identified using Read-coding in CPRD were close to those estimated in HSE (men: 80% current drinkers in CPRD, 83% in HSE; women: 73% current drinkers in CPRD, 78% in HSE). Units per week were also broadly comparable between CPRD and HSE although prevalence of higher risk drinking in CPRD looked slightly lower (**Table 3**). However, for estimates for both the proportion of current drinkers and units consumed per week were slightly lower in CPRD than HSE.

Time trends

Figure 1 illustrates time trends in recording of alcohol status and level of consumption. Between 2009 and 2018, there was an increase in recording in CPRD of both alcohol status and level of alcohol consumption from 19% to over 50%.

Frequency of recording

Among patients with at least one alcohol record in the last five years, 51.0% had just one record, 21.1% had two records, 10.3% had three records, and the remaining 17.7% had four or more records. 51.4% of patients with a record had their first alcohol record on the date of registration, 84.4% within the first month, and 95.5% within the first year of registration.

For peer review only - http://bmjopen.bmj.com/site/about/guidelines.xhtml

Sensitivity analyses

1. Internal validity where we required that AUDIT and units were recorded within 30 days of another code (rather than on the same date) resulted in more records being validated, but the results were identical to the main analysis (**Appendix Table 2**).

2. Individuals with diabetes mellitus, hypertension and liver disease diagnosed in the five years prior to the study date had slightly higher alcohol recording than shown in the main analysis (where these variables included all patients ever diagnosed) (Diabetes 84.7% with recorded alcohol use in sensitivity analysis compared to 82.9% in main analysis with diabetes ever recorded; hypertension 75.8% with recorded alcohol use in sensitivity analysis, compared to 72.6% in main analysis; and liver disease 77.0% with recorded alcohol use in sensitivity analysis, compared to 69.9% in main analysis).

3. Similarly, depression and anxiety diagnosed in the past year had higher levels of alcohol recording than when diagnosed in the previous five years (depression: 62.4% vs 60.9% in main analysis; anxiety: 62.0% vs 60.9% in main analysis).

For peer review only

For peer review only - http://bmjopen.bmj.com/site/about/guidelines.xhtml

Discussion

Summary

In 2018, information about alcohol consumption was available for roughly half of all adult patients registered with practices contributing to the CPRD. Large differences in recording were seen across patient characteristics, in particular increasing with age, deprivation, and higher in patients with liver disease, hypertension, diabetes mellitus, depression and anxiety. Patients who were missing data on other risk factors such as smoking, BMI and ethnicity were also less likely to have any recording of alcohol use. Those with missing alcohol, smoking, BMI and ethnicity data may represent a group who have minimal contact with primary care. However, even among those with smoking recorded, GPs only recorded drinking status in approximately 50%-60% of patients. This suggests that GPs record alcohol use less frequently than smoking status; so even when there are opportunities to ask about health behaviours, alcohol use in not necessarily included. Alcohol was more likely to be recorded in those with higher levels of other health risks (smokers, obese) also suggesting that GPs may be more likely to ask about alcohol in patients who they perceive as at higher risk. These patients may also visit the GP more frequently, increasing the opportunities to ask about alcohol use.

The patterns of alcohol recording seen here show that UK GPs maybe failing to record alcohol consumption at the most basic level of drinking status for half of their adult population. However, patients who are more at risk are being appropriately targeted (those with mental health conditions and diabetes, liver disease, hypertension) have better recording. Importantly, 20-40% of these at-risk patients still lack data on alcohol consumption and the proportion of patients screened for alcohol consumption appropriately using a valid screening tool is even lower (although it is possible that individuals who score at low risk are being screened but these data are not being recorded). Alcohol use should be discussed as part of the cardiovascular disease health check for all patients over 40,[17]

For peer review only - http://bmjopen.bmj.com/site/about/guidelines.xhtml

and suboptimal levels of alcohol recording are consistent with the low uptake of the health check recently described.[18] Importantly, just one third of 18-24 year olds had alcohol consumption recorded, and they are particularly at risk from risk drinking behaviours.

Compared to a 2013 study using CPRD data (Khadjesari et al, 2013[19]), our study showed that while recording among new registrants has plateaued since 2009, remaining at around 75%, screening with AUDIT appears to have increased from 9% among new registrants in 2009 to 21.8% in our study. This corresponds to the introduction of new NICE guidance in 2010.[4] However, screening with a recommended screening tool for all registered adults remains at only 8%.

Our internal validation showed that, when there was a record of level of alcohol consumption, there was more likely to be a corresponding record of other measures of intake recorded in CPRD. Recording of units per week corresponded to AUDIT scores, and both measures corresponded to informal coding of alcohol consumption using Read codes (e.g. codes for light/moderate/heavy drinker). AUDIT scores were used as the ‘gold standard’ because they are considered valid in comparison with other self-reported alcohol measures,[20, 21] but all self-reported measures of alcohol use are limited as they are likely to be under-reported.[22]

The Health Survey for England found that, in 2016, 17% of men and 22% of women were teetotal. Using CPRD drinking status, 20.5% of patients were recorded as non-drinkers, so this is comparable and demonstrates external validity, albeit also self-reported. The internal and external validity demonstrated in this study give weight to any study using alcohol data from CPRD. While care must be taken to avoid introducing bias by restricting to only patients with alcohol recorded, the measures of consumption are likely to be useful in ranking participants in terms of their alcohol of consumption, with the caveats required by all self-reported alcohol research.

For peer review only - http://bmjopen.bmj.com/site/about/guidelines.xhtml

Strengths and Limitations

This study investigated alcohol recording across nearly 1.8 million UK adults, providing the power to explore recording in different subgroups of patients, and to understand in detail how alcohol use is recorded in primary care.

To validate, we used AUDIT scores recorded on the same date as the codes of interest. If an AUDIT questionnaire is completed, subsequent recording of alcohol consumption (in Read codes or units per week) might be more accurate than at a time when no scoring is done, and validity may be lower outside of these times. Additionally, GP practices that record both measures during the same consultation may be different to practices that only record one measure, and perhaps more likely to record alcohol use measures accurately. However, our sensitivity analyses using AUDIT scores recorded within 30 days before or after the date of the alcohol-related code of interest showed that records within a month of an AUDIT score or measure of units were internally consistent. For the external validation we used data from the health survey for England 2016, whereas the time frame for the prevalence estimates of alcohol use from CPRD were from 2013-2018 however although the time frames do not match perfectly 2016 is within the middle of the time frame and this seems an appropriate comparator.

Though we have demonstrated that alcohol recording and screening are higher for patients with certain high-risk conditions, it does not mean that GPs have acted upon the screening result, or that they have screened at an appropriate time. For example, patients with depression or anxiety may not have been screened at the time when depression was diagnosed. We showed that the majority of screening took place within one month of registration, so whether this is repeated during consultations for high-risk conditions is not clear from these data.

For peer review only

Between 2009 and 2018, the proportion of CPRD patients with alcohol recorded has more than tripled from around 13% to nearly 49%. This is likely to be driven by the 2010 NICE guidelines recommending screening at the time of registration. Screening is most likely to happen at the time of registration, shown here and in Khadjesari et al[19], and therefore the increase in recording shown here may be linked to patient turnover. The result is that patients who have been at a practice for a long time are much less likely to have any record of their alcohol consumption; while recording has improved, there are clear and important differences between patients with and without a record for alcohol use.

For peer review only

Implications for research

This study helps establish best practise in using primary care data to investigate alcohol use in research. Researchers using CPRD data should be aware of the limitations of alcohol data. Though the validity of the existing data has been described here, using only patients with complete alcohol use data may introduce selection bias into studies by preferentially including older, sicker, and more deprived patients.

Implications for practice

This study provides insight into how well aligned routine primary care practice is with NICE guidelines for alcohol screening. Recording of alcohol in primary care is not complete, meaning that opportunities for intervention may not be being identified. While we found evidence that alcohol use recording is higher in the high-risk groups highlighted by NICE (those with hypertension, depression), and at key times of patient registration recommended by NICE (among newly registered patients), there are still large numbers of patients in whom we might expect risky drinking who are not having their alcohol use recorded, and who therefore may not be targeted for intervention. In particular, younger individuals, in whom alcohol is a major cause of morbidity, maybe being overlooked.

For peer review only - http://bmjopen.bmj.com/site/about/guidelines.xhtml

References

1. Home Office, *A minimum unit price for alcohol. Impact Assessment.* 2012.
2. GBD 2016 Alcohol Collaborators, *Alcohol use and burden for 195 countries and territories, 1990-2016: a systematic analysis for the Global Burden of Disease Study 2016.* Lancet, 2018. **392**(10152): p. 1015-1035.
3. Babor, T.F. and J.C. Higgins-Biddle, *BRIEF INTERVENTION For Hazardous and Harmful Drinking: A Manual for Use in Primary Care.* 2001.
4. NICE, *Alcohol-use disorders: prevention. NICE guidelines PH24.* 2010.
5. Public Health England. *NHS Health Check: Best practice guidance*. 2017; Available from: http://www.healthcheck.nhs.uk/document.php?o=1308.
6. Stewart, D., et al., *Alcohol consumption and all-cause mortality: an analysis of general practice database records for patients with long-term conditions.* J Epidemiol Community Health, 2017. **71**(8): p. 729-735.
7. Otete, H.E., et al., *Sex and age differences in the early identification and treatment of alcohol use: a population-based study of patients with alcoholic cirrhosis.* Addiction, 2015. **110**(12): p. 1932-40.
8. Mathur, R., et al., *Completeness and usability of ethnicity data in UK-based primary care and hospital databases.* J Public Health, 2014. **36**(4): p. 684-92.
9. Bhaskaran, K., et al., *Representativeness and optimal use of body mass index (BMI) in the UK Clinical Practice Research Datalink (CPRD).* BMJ Open, 2013. **3**(9): p. e003389.
10. Babor, T.F., et al. *The Alcohol Use Disorders Identification Test: Guidelines for Use in Primary Care*. 2001; Available from: http://www.talkingalcohol.com/files/pdfs/WHO_audit.pdf.
11. Saunders, J.B., et al., *Development of the Alcohol Use Disorders Identification Test (AUDIT): WHO Collaborative Project on Early Detection of Persons with Harmful Alcohol Consumption--II.* Addiction, 1993. **88**(6): p. 791-804.
12. Babor, T., et al., *The Alcohol Use Disorders Identification Test: Guidelines for Use in Primary Care. Available from http://apps.who.int/iris/bitstream/handle/10665/67205/WHO_MSD_MSB_01.6a.pdf?sequence=1*. 2001.
13. Reinert, D.F. and J.P. Allen, *The alcohol use disorders identification test: an update of research findings.* Alcohol Clin Exp Res, 2007. **31**(2): p. 185-99.
14. Hodgson, R., et al., *The FAST Alcohol Screening Test.* Alcohol Alcohol, 2002. **37**(1): p. 61-6.
15. Hodgson, R.J., et al., *Fast screening for alcohol misuse.* Addict Behav, 2003. **28**(8): p. 1453-63.
16. NHS Digital, *Health Survey for England, 2016.* 2016.
17. Public Health England, *NHS Health Check: Best practice guidance. 2017; Available from: http://www.healthcheck.nhs.uk/document.php?o=1308*. 2017.
18. Coghill, N., et al., *NHS health checks: a cross- sectional observational study on equity of uptake and outcomes.* BMC Health Serv Res, 2018. **18**(1): p. 238.
19. Khadjesari, Z., et al., *Alcohol consumption screening of newly-registered patients in primary care: a cross-sectional analysis.* Br J Gen Pract, 2013. **63**(615): p. e706-12.
20. Allen, J.P., et al., *A review of research on the Alcohol Use Disorders Identification Test (AUDIT).* Alcohol Clin Exp Res, 1997. **21**(4): p. 613-9.
21. Reinert, D.F. and J.P. Allen, *The Alcohol Use Disorders Identification Test (AUDIT): A Review of Recent Research.* Alcoholism: Clinical and Experimental Research, 2002. **26**(2): p. 272-279.
22. Feunekes, G.I., et al., *Alcohol intake assessment: the sober facts.* Am J Epidemiol, 1999. **150**(1): p. 105-12.

For peer review only

Author statement

All authors contributed to the design of the study. EH and EC extracted the data, EC, KM and EH wrote the statistical programmes, and wrote the first draft. All authors contributed to further drafts and approved the final manuscript.

Conflicts of interest

None.

Funding

This research received no specific grant from any funding agency in the public, commercial or not-for-profit sectors. EH holds a NIHR postdoctoral fellowship (grant number PDF-2016-09-029).

Role of the funding source

The NIHR had no role in the design, analysis or writing up of this study.

Patient and public involvement

We did not involve patients or the public in our work.

Data access

EC and EH had full access to the CPRD database in order to create the study population.

Data sharing statement

We obtained data from the Clinical Practice Research Datalink (CPRD). CPRD is a research service that provides primary care and linked data for public health research. CPRD data governance, and our own license to use CPRD data, do not allow us to distribute or make available patient data directly to other parties. Researchers may apply for data access at www.cprd.com, and must have their study protocol approved by the Independent Scientific Advisory Committee for MHRA database research (details at www.cprd.com/isac).

Ethical approval

This study was approved by the London School of Hygiene and Tropical Medicine Ethics Committee (Approval Number 14454).

For peer review only - http://bmjopen.bmj.com/site/about/guidelines.xhtml

Tables

For peer review only

For peer review only - http://bmjopen.bmj.com/site/about/guidelines.xhtml

Table 1 Characteristics of adult patients registered in the CPRD, on 1st January 2018, and those with records indicating drinking status, level of consumption, units per week and AUDIT/FAST scores.

		Individuals in the study population with recording of alcohol use in different categories in the 5 years before 1st January 2018**							
	**Study population: All individuals 18+yrs registered with a CPRD practice on 1st January 2018* N (col %)***	Has a record for any Read code relating to alcohol use n (row %)**	Has a record for a Read code indicating alcohol use, from which alcohol status could be derived[a] n (row %)**	Has a record of any drinking status recorded as Read code, or captured through structured data areas in GP software[b] n (row %)**	Has level of consumption recorded in Read codes[c] n (row %)**	Has units per week recorded n (row %)**	Has had AUDIT or FAST screen n (row %)**	Has AUDIT or FAST score recorded n (row %)**	Has any level of drinking recorded in Read, units or score[d] n (row %)**
**N**	1,768,651 (100)	918,254 (51.9)	897,429 (50.7)	862,330 (48.8)	558,747 (31.6)	431,394 (24.4)	194,155 (11)	144,739 (8.2)	862,191 (48.7)
**Sex**									
Male	872,865 (49.4)	416,611 (47.7)	406,435 (46.6)	389,946 (44.7)	231,204 (26.5)	222,678 (25.5)	89,070 (10.2)	66,782 (7.7)	389,565 (44.6)
Female	895,786 (50.6)	501,643 (56)	490,994 (54.8)	472,384 (52.7)	327,543 (36.6)	208,716 (23.3)	105,085 (11.7)	77,957 (8.7)	472,626 (52.8)
**Age group**									
18-24	168,796 (9.5)	58,379 (34.6)	54,806 (32.5)	51,584 (30.6)	36,755 (21.8)	16,537 (9.8)	15,106 (8.9)	8,239 (4.9)	51,593 (30.6)
25-34	285,015 (16.1)	131,905 (46.3)	128,030 (44.9)	119,772 (42)	76,097 (26.7)	50,686 (17.8)	32,416 (11.4)	24,153 (8.5)	120,720 (42.4)
35-44	300,819 (17)	136,265 (45.3)	133,263 (44.3)	125,865 (41.8)	77,277 (25.7)	60,492 (20.1)	32,199 (10.7)	25,210 (8.4)	126,630 (42.1)
45-54	333,132 (18.8)	171,021 (51.3)	167,281 (50.2)	160,742 (48.3)	96,024 (28.8)	88,593 (26.6)	35,756 (10.7)	27,678 (8.3)	160,524 (48.2)
55-64	277,217 (15.7)	160,137 (57.8)	157,172 (56.7)	152,786 (55.1)	94,656 (34.1)	86,476 (31.2)	30,844 (11.1)	23,691 (8.5)	151,968 (54.8)
65-74	223,120 (12.6)	145,105 (65)	142,780 (64)	139,320 (62.4)	92,173 (41.3)	77,896 (34.9)	28,810 (12.9)	21,768 (9.8)	139,038 (62.3)
75+	180,552 (10.2)	115,442 (63.9)	114,097 (63.2)	112,261 (62.2)	85,765 (47.5)	50,714 (28.1)	19,024 (10.5)	14,000 (7.8)	111,718 (61.9)
**Time since registration**									
1-5 years	334,629 (18.9)	274,706 (82.1)	266,739 (79.7)	246,126 (73.6)	150,578 (45)	114,320 (34.2)	96,728 (28.9)	72,714 (21.7)	252,733 (75.5)
>=5 years	1,434,022 (81.1)	643,548 (44.9)	630,690 (44)	616,204 (43)	408,169 (28.5)	317,074 (22.1)	97,427 (6.8)	72,025 (5)	609,458 (42.5)
**Region**									
North England	231,297 (13.1)	138,506 (59.9)	136,235 (58.9)	128,522 (55.6)	76,323 (33)	76,396 (33)	45,013 (19.5)	39,228 (17)	132,862 (57.4)
South England	607,016 (34.3)	320,297 (52.8)	307,980 (50.7)	285,002 (47)	174,695 (28.8)	143,027 (23.6)	100,624 (16.6)	74,311 (12.2)	295,299 (48.6)
Northern Ireland	114,311 (6.5)	55,838 (48.8)	53,206 (46.5)	52,981 (46.3)	34,830 (30.5)	22,297 (19.5)	11,158 (9.8)	1,029 (0.9)	50,614 (44.3)
Scotland	397,133 (22.5)	207,247 (52.2)	205,158 (51.7)	201,171 (50.7)	138,776 (34.9)	103,816 (26.1)	35,719 (9)	29,606 (7.5)	197,830 (49.8)
Wales	418,894 (23.7)	196,366 (46.9)	194,850 (46.5)	194,654 (46.5)	134,123 (32)	85,858 (20.5)	1,641 (0.4)	565 (0.1)	185,586 (44.3)
**Deprivation**									
1 (least deprived)	190,262 (10.8)	102,692 (54)	99,615 (52.4)	90,969 (47.8)	44,305 (23.3)	57,838 (30.4)	32,524 (17.1)	25,803 (13.6)	95,697 (50.3)
2	134,191 (7.6)	74,536 (55.5)	72,401 (54)	66,664 (49.7)	35,727 (26.6)	39,943 (29.8)	24,338 (18.1)	19,773 (14.7)	69,775 (52)
3	118,777 (6.7)	67,455 (56.8)	65,695 (55.3)	61,953 (52.2)	36,121 (30.4)	33,892 (28.5)	18,936 (15.9)	14,683 (12.4)	63,131 (53.2)
4	108,991 (6.2)	62,745 (57.6)	61,655 (56.6)	58,234 (53.4)	39,674 (36.4)	26,879 (24.7)	18,285 (16.8)	15,214 (14)	59,323 (54.4)
5 (most deprived)	96,301 (5.4)	57,840 (60.1)	56,793 (59)	51,770 (53.8)	38,342 (39.8)	21,983 (22.8)	20,646 (21.4)	17,690 (18.4)	54,925 (57)
Missing	1,120,129 (63.3)	552,986 (49.4)	541,270 (48.3)	532,740 (47.6)	364,578 (32.5)	250,859 (22.4)	79,426 (7.1)	51,576 (4.6)	519,340 (46.4)

Continued…

For peer review only

	Study population: All individuals 18+yrs registered with a CPRD practice on 1st January 2018* N (col %)*	Individuals in the study population with recording of alcohol use in different categories in the 5 years before 1st January 2018**							
		Has a record for any Read code relating to alcohol use n (row %)**	Has a record for a Read code indicating alcohol use, from which alcohol status could be derived[a] n (row %)**	Has a record of any drinking status recorded as Read code, or captured through structured data areas in GP software[b] n (row %)**	Has level of consumption recorded in Read codes[c] n (row %)**	Has units per week recorded n (row %)**	Has had AUDIT or FAST screen n (row %)**	Has AUDIT or FAST score recorded n (row %)**	Has any level of drinking recorded in Read, units or score[d] n (row %)**
**Ethnicity**									
White	807,709 (45.7)	502,125 (62.2)	489,716 (60.6)	465,333 (57.6)	287,175 (35.6)	254,409 (31.5)	129,035 (16)	100,277 (12.4)	470,166 (58.2)
South Asian	46,593 (2.6)	28,089 (60.3)	27,115 (58.2)	25,603 (55)	21,297 (45.7)	5,874 (12.6)	8,991 (19.3)	6,601 (14.2)	26,298 (56.4)
Other	60,520 (3.4)	38,086 (62.9)	36,478 (60.3)	33,898 (56)	26,559 (43.9)	9,974 (16.5)	13,413 (22.2)	9,711 (16)	35,182 (58.1)
Missing	853,829 (48.3)	349,954 (41)	344,120 (40.3)	337,496 (39.5)	223,716 (26.2)	161,137 (18.9)	42,716 (5)	28,150 (3.3)	330,545 (38.7)
**Smoking status**									
Non smoker	746,074 (42.2)	383,302 (51.4)	373,539 (50.1)	356,424 (47.8)	233,122 (31.2)	163,733 (21.9)	86,740 (11.6)	63,611 (8.5)	358,765 (48.1)
Current smoker	254,850 (14.4)	147,616 (57.9)	144,248 (56.6)	139,028 (54.6)	90,505 (35.5)	68,758 (27)	30,019 (11.8)	22,509 (8.8)	137,027 (53.8)
Ex-smoker	565,583 (32)	347,945 (61.5)	342,137 (60.5)	332,036 (58.7)	213,908 (37.8)	181,109 (32)	68,405 (12.1)	51,754 (9.2)	331,063 (58.5)
Missing	202,144 (11.4)	39,391 (19.5)	37,505 (18.6)	34,842 (17.2)	21,212 (10.5)	17,794 (8.8)	8,991 (4.4)	6,865 (3.4)	35,336 (17.5)
**BMI**									
Normal (18·5–25kg/m²)	533,130 (30.1)	298,415 (56)	290,338 (54.5)	277,070 (52)	172,796 (32.4)	139,540 (26.2)	70,358 (13.2)	52,154 (9.8)	278,432 (52.2)
Underweight (BMI <18·5kg/m²)	32,421 (1.8)	16,915 (52.2)	16,261 (50.2)	15,481 (47.7)	10,904 (33.6)	6,055 (18.7)	4,165 (12.8)	2,823 (8.7)	15,559 (48)
Overweight/obese (BMI ≥25kg/m²)	865,940 (49)	543,118 (62.7)	534,200 (61.7)	519,336 (60)	346,490 (40)	265,441 (30.7)	105,454 (12.2)	79,924 (9.2)	516,428 (59.6)
Missing	337,160 (19.1)	59,806 (17.7)	56,630 (16.8)	50,443 (15)	28,557 (8.5)	20,358 (6)	14,178 (4.2)	9,838 (2.9)	51,772 (15.4)
**Liver disease**	35,542 (2)	25,404 (71.5)	24,858 (69.9)	24,340 (68.5)	16,978 (47.8)	12,847 (36.1)	4,851 (13.6)	3,607 (10.1)	24,059 (67.7)
**Hypertension**	358,285 (20.3)	262,785 (73.3)	260,058 (72.6)	256,762 (71.7)	183,616 (51.2)	134,479 (37.5)	42,598 (11.9)	31,994 (8.9)	254,115 (70.9)
**Diabetes**	120,744 (6.8)	100,787 (83.5)	100,145 (82.9)	99,378 (82.3)	79,211 (65.6)	47,668 (39.5)	15,600 (12.9)	11,874 (9.8)	98,436 (81.5)
**Depression**	118,644 (6.7)	73,868 (62.3)	72,298 (60.9)	69,961 (59)	47,499 (40)	33,663 (28.4)	14,841 (12.5)	10,805 (9.1)	69,043 (58.2)
**Anxiety**	80,658 (4.6)	50,048 (62)	49,090 (60.9)	47,593 (59)	31,840 (39.5)	23,330 (28.9)	9,624 (11.9)	7,075 (8.8)	46,976 (58.2)

a. Alcohol status: Current drinker, ex-drinker, non-drinker from Read codes.
b. Alcohol status: Current drinker, ex-drinker, non-drinker from Read codes and captured through structured data areas in GP software.
c. Level of drinking: none, light, moderate, heavy drinker
d. Level of drinking derived from: Read codes, units per week or AUDIT/FAST screening score

*For the column (shaded) summarising characteristics for the whole study population (i.e. all adults registered in CPRD on 1st January 2018) – percentages are out of the whole study population (i.e. column %s).
**For subsequent columns (unshaded) – which cover individuals identified through different types of alcohol-use recording – percentages represent the number of individuals recorded with the specific category of coding as a percentage of the study population in that particular stratum; That is, row percentage with denominator from study population in that stratum (e.g. for all women in the study population [n=895,786], 56% [n=501,643] have a record of any Read code relating to alcohol use).

For peer review only

For peer review only - http://bmjopen.bmj.com/site/about/guidelines.xhtml

Table 2 Internal validation comparing measures of alcohol use in CPRD to AUDIT scores, and to units per week. N=1,768,651

	N	(%)	N patients with AUDIT score on the same date as record n	(%)	AUDIT score median	(IQR)	N patients with units per week recorded on same date as record n	(%)	Units per week median	(IQR)
**Current drinking status***										
Non	175,022	(20.3)	2927	(1.7)	0	(0-0)	913	(0.5)	0	(0-1)
Ex	31,525	(3.7)	307	(1)	0	(0-0)	999	(3.2)	0	(0-1)
Current	655,783	(76)	22739	(3.5)	3	(1-5)	333,371	(50.8)	6	(2-14)
**Current drinking level (Read codes)**										
Non-drinker	222830	(39.9)	3246	(1.5)	0	(0-0)	2,271	(1)	0	(0-2)
Light drinker	255,922	(45.8)	3,298	(1.3)	2	(1-3)	19,065	(7.4)	3	(1-8)
Moderate drinker	52,066	(9.3)	1,216	(2.3)	0.6	(0-4)	12,762	(24.5)	6	(2-14)
Heavy drinker	27,929	(5)	2,803	(10)	6	(5-9)	10,315	(36.9)	18	(9-30)
**Units per week**										
0	38,917	(9)	1,834	(4.7)	0	(0-0)				
1-14	298,463	(69.2)	11,601	(3.9)	3	(2-4)	n/a		n/a	
15-42	80,434	(18.6)	2,730	(3.4)	6	(4-8)				
43+	13,580	(3.1)	215	(1.6)	11	(8-12)				
**AUDIT score category**										
0-7; Alcohol Education	79,764	(92.8)					14,901	(18.7)	4	(2-10)
8-15; Simple Advice	5,752	(6.7)					1,415	(24.6)	20	(12-30)
16-19; Simple Advice plus Brief Counselling and Continued Monitoring	194	(0.2)	n/a		n/a		36	(18.6)	41	(23.5-60)
20-40; Referral to Specialist for Diagnostic Evaluation and Treatment	213	(0.2)					28	(13.1)	60	(25-79)

*Current drinking status based on recorded status in Read codes and captured through structured data areas in GP software (not including derived status based on units consumed or AUDIT score)

For peer review only - http://bmjopen.bmj.com/site/about/guidelines.xhtml

Table 3 External validation, comparing most recent record of alcohol status in CPRD to data from the Health Survey for England 2016.

	CPRD		HSE
	**n**	**%**	**%**
**Men**			
**Non-drinker***	76,597	20	17
**Current drinker**	313,349	80	83
**Units per week among current drinkers with units per week recorded:**			
≤14 (lower risk)	*122,747*	*67*	*63*
15-50 (increased risk)	*53,917*	*30*	*31*
>50 (higher risk)	*5,996*	*3*	*6*
**Women**			
**Non-drinker***	129,950	28	22
**Current drinker**	342,434	73	78
**Units per week among current drinkers with units per week recorded:**			
≤14 (lower risk)	*146,183*	*88*	*78*
15-35 (increased risk)	*16,431*	*10*	*16*
>35 (higher risk)	*3,096*	*2*	*5*

*Includes patients labelled non- and ex-drinker

For peer review only

Figures

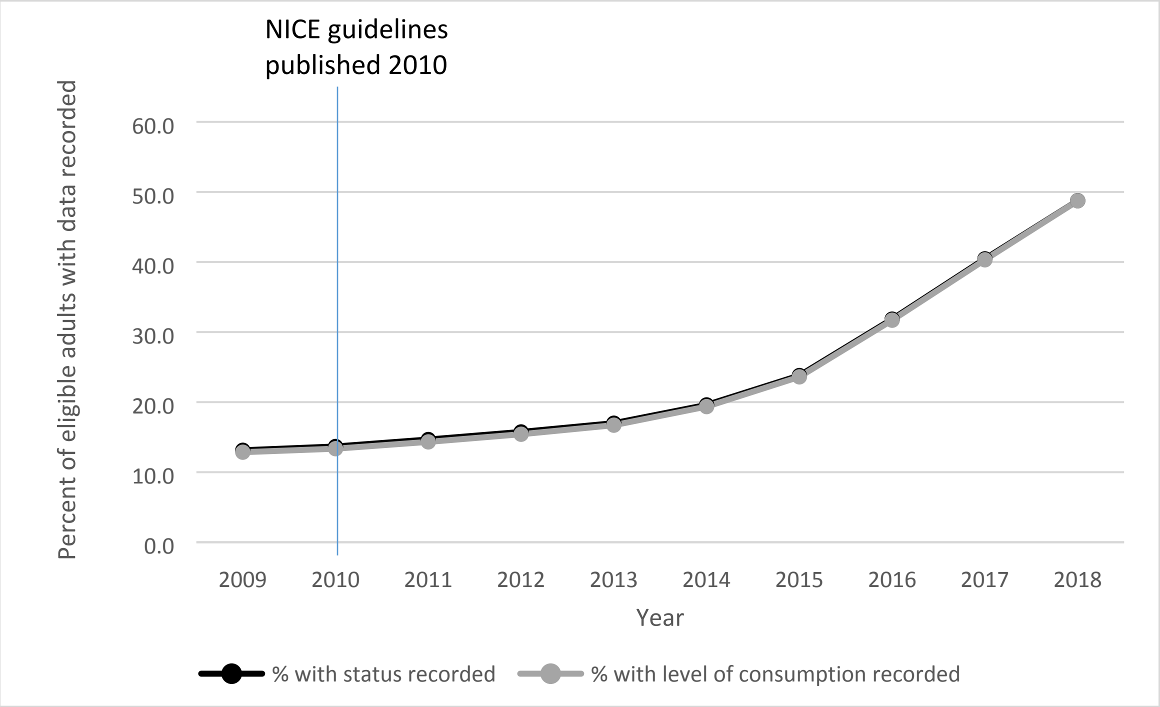

Figure 1. Recording of alcohol status and time trends between 2009 and 2018.

For peer review only

For peer review only - http://bmjopen.bmj.com/site/about/guidelines.xhtml

Appendices

Detailed variable definitions:

i. Any code suggesting that alcohol was discussed in the consultation (Appendix Table 1, column 1).

ii. Codes indicating AUDIT or FAST screening (Appendix Table 1, columns 4 and 5)

iii. Codes quantifying alcohol use, comprised of:

a. Drinking status (categorised as current drinker, ex drinker, non drinker; recorded in:
   - Read codes (Appendix Table 1, column 2)
   - Structured data, using data from the CPRD Additional Clinical Details file recorded under Entity Type 5, data field 1 (status) (Entity Type is the identifier that represents the structured data area in Vision where the data was entered)

b. Level of drinking (non, light, moderate, heavy drinker; as recorded in Read codes (Appendix Table 1, Column 3))

c. Alcohol units consumed per week, using an existing (CALIBER: https://www.ucl.ac.uk/health-informatics/caliber) variable definition for 'number of alcohol unit consumption per week recorded in primary care (CPRD)',[1] using data from the CPRD Additional Clinical Details file recorded under Entity Type 5, data field 2 (units per week). We categorised units per week as: "None", "1-14, "15-42" or "43+" units per week. 14 units per week is the recommended limit for men and women according to current UK government guidelines.[2]

d. AUDIT or FAST scores (AUDIT categorised according to WHO guidelines on risk level: 1-7 - low risk drinking; 8-15 - hazardous drinking; 16-19 - harmful drinking; 20-40 – possible dependence.[3]). Scores resulting from AUDIT/FAST screening (i.e. associated with a Read morbidity code

For peer review only

indicating use of AUDIT/FAST), were obtained from the Additional Clinical Details file under Entity Type 172.

For peer review only

Table 1. Read codes for alcohol data in CPRD

For peer review only

medcode	readcode	readterm	1 Any alcohol-related code	2 Current drinking status	3 Codes indicating level of alcohol use	4 AUDIT screening	5 FAST screening
47555	90.95	cerebral degeneration due to alcoholism	1	0	0	0	0
47238	0C5..12	drink processor	0	0	0	0	0
27	136..00	alcohol consumption	1	0	0	0	0
12949	1361.00	Teetotaller	1	non	non	0	0
12970	1361.11	non drinker alcohol	1	non	non	0	0
4447	1361.12	non-drinker alcohol	1	non	non	0	0
12975	1362.00	trivial drinker - <1u/day	1	curr	L	0	0
385	1362.11	drinks rarely	1	curr	L	0	0
749	1362.12	drinks occasionally	1	curr	L	0	0
12972	1363.00	light drinker - 1-2u/day	1	curr	L	0	0
322	1364.00	moderate drinker - 3-6u/day	1	curr	M	0	0
1618	1365.00	heavy drinker - 7-9u/day	1	curr	H	0	0
12977	1366.00	very heavy drinker - >9u/day	1	curr	H	0	0
967	1367.00	stopped drinking alcohol	1	ex	non	0	0
12978	1368.00	alcohol consumption unknown	1	0	0	0	0
12976	1369.00	suspect alcohol abuse - denied	1	0	0	0	0
22933	136A.00	ex-trivial drinker (<1u/day)	1	0	0	0	0
102665	136a.00	increasing risk drinking	1	curr	H	0	0
26471	136B.00	ex-light drinker - (1-2u/day)	1	0	0	0	0
99877	136b.00	feels should cut down drinking	1	curr	H	0	0
19495	136C.00	ex-moderate drinker - (3-6u/d)	1	0	0	0	0
102448	136c.00	higher risk drinking	1	curr	H	0	0
19493	136D.00	ex-heavy drinker - (7-9u/day)	1	0	0	0	0
103230	136d.00	lower risk drinking	1	curr	M	0	0
12983	136E.00	ex-very heavy drinker-(>9u/d)	1	0	0	0	0
106560	136e.00	declines to state current alcohol consumption	1	0	0	0	0
12971	136F.00	spirit drinker	1	curr	0	0	0
2689	136G.00	beer drinker	1	curr	0	0	0
12968	136H.00	drinks beer and spirits	1	curr	0	0	0
12969	136I.00	drinks wine	1	curr	0	0	0
956	136J.00	social drinker	1	curr	M	0	0

For peer review only

12982	136K.00	alcohol intake above recommended sensible limits	1	curr	H	0	0
26472	136L.00	alcohol intake within recommended sensible limits	1	curr	M	0	0
12979	136M.00	current non drinker	1	non	non	0	0
12980	136N.00	light drinker	1	curr	L	0	0
12985	136O.00	moderate drinker	1	curr	M	0	0
8999	136P.00	heavy drinker	1	curr	H	0	0
12984	136Q.00	very heavy drinker	1	curr	H	0	0
19401	136R.00	binge drinker	1	curr	H	0	0
19494	136S.00	hazardous alcohol use	1	curr	H	0	0
30695	136T.00	harmful alcohol use	1	curr	H	0	0
93415	136V.00	alcohol units per week	1	0	0	0	0
94670	136W.00	alcohol misuse	1	curr	H	0	0
97126	136X.00	alcohol units consumed on heaviest drinking day	1	0	0	0	0
101718	136Y.00	drinks in morning to get rid of hangover	1	curr	0	0	0
12981	136Z.00	alcohol consumption nos	1	0	0	0	0
18156	13Y8.00	alcoholics anonymous	1	0	0	0	0
84218	13ZY.00	disqualified from driving due to excess alcohol	1	0	0	0	0
8430	1462.00	h/o: alcoholism	1	0	0	0	0
38061	1B1c.00	alcohol induced hallucinations	1	curr	0	0	0
44783	1D19.00	pain in lymph nodes after alcohol consumption	1	0	0	0	0
104649	1F9D.00	replaces meals with drinks	0	0	0	0	0
24735	2577.00	o/e - breath - alcohol smell	1	curr	0	0	0
10161	2577.11	o/e - alcoholic breath	1	curr	0	0	0
69691	388j.00	cage questionnaire	1	0	0	0	0
70161	388j.00	cage questionnaire	1	0	0	0	0
93624	388u.00	fast alcohol screening test	1	0	0	0	1
96107	38D2.00	single alcohol screening questionnaire	1	0	0	0	0
94838	38D3.00	alcohol use disorders identification test	1	0	0	1	0
95744	38D4.00	alcohol use disorder identificatn test consumptn questionnre	1	0	0	1	0
97501	38D5.00	alcoh use disor id test piccinelli consumption questionnaire	1	0	0	1	0
100493	38Df.00	five-shot questionnaire on heavy drinking	1	0	0	0	0
102577	38Dz.00	severity of alcohol dependence questionnaire	1	0	0	0	0
102770	38Dz.11	sadq - severity of alcohol dependence questionnaire	1	0	0	0	0

For peer review only

100989	4I91.11	breath alcohol level	1	0	0	0	0
17266	63C7.00	maternal alcohol abuse	1	0	0	0	0
12442	66e..00	alcohol disorder monitoring	1	curr	0	0	0
32964	66e0.00	alcohol abuse monitoring	1	0	0	0	0
11491	6792.00	health ed. - alcohol	1	0	0	0	0
35859	67A5.00	pregnancy alcohol advice	1	0	0	0	0
18711	67H0.00	lifestyle advice regarding alcohol	1	0	0	0	0
105144	67K6.00	cycle of change stage, alcohol	1	0	0	0	0
19489	6892.00	alcohol consumption screen	1	0	0	0	0
9264	68S..00	alcohol consumption screen	1	0	0	0	0
56410	7P22100	delivery of rehabilitation for alcohol addiction	1	0	0	0	0
2083	8BA8.00	alcohol detoxification	1	0	0	0	0
7692	8CAM.00	patient advised about alcohol	1	0	0	0	0
102564	8CAM000	advised to abstain from alcohol consumption	1	0	0	0	0
97309	8CAv.00	advised to contact primary care alcohol worker	1	curr	0	0	0
37264	8CE1.00	alcohol leaflet given	1	0	0	0	0
29691	8G32.00	aversion therapy - alcoholism	1	0	0	0	0
21650	8H35.00	admitted to alcohol detoxification centre	1	0	0	0	0
9849	8H7p.00	referral to community alcohol team	1	0	0	0	0
12554	8HHe.00	referral to community drug and alcohol team	1	0	0	0	0
94553	8HkG.00	referral to specialist alcohol treatment service	1	0	0	0	0
96993	8HkJ.00	referral to alcohol brief intervention service	1	0	0	0	0
94669	8IA7.00	alcohol consumption screening test declined	1	0	0	0	0
97261	8IAF.00	brief intervention for excessive alcohol consumptn declined	1	curr	0	0	0
97680	8IAJ.00	declined referral to specialist alcohol treatment service	1	curr	0	0	0
102247	8IAt.00	extended interven for excessive alcohol consumption declined	1	curr	0	0	0
103459	8IEA.00	referral to community alcohol team declined	1	curr	0	0	0
43813	9EQ..11	police:venesect-alcohol	1	0	0	0	0
102121	9EQ..12	police:venesect-alcohol	1	0	0	0	0
11740	9k1..00	alcohol misuse - enhanced services administration	1	curr	0	0	0
35330	9k11.00	alcohol consumption counselling	1	curr	0	0	0
63529	9k12.00	alcohol misuse - enhanced service completed	1	0	0	0	0
48545	9k13.00	alcohol questionnaire completed	1	0	0	0	0

For peer review only

47123	9k14.00	alcohol counselling by other agencies	1	0	0	0	0
90714	9k15.00	alcohol screen - audit completed	1	0	0	1	0
94963	9k16.00	alcohol screen - fast alcohol screening test completed	1	0	0	0	1
95663	9k17.00	alcohol screen - audit c completed	1	0	0	1	0
94485	9k18.00	alcohol screen - audit pc completed	1	0	0	1	0
95944	9k19.00	alcohol assesment declined - enhanced services admin	1	0	0	0	0
96259	9k19.11	alcohol assessment declined	1	0	0	0	0
96053	9k1A.00	brief intervention for excessive alcohol consumptn completed	1	0	0	0	0
96054	9k1B.00	extended intervention for excessive alcohol consumptn complt	1	0	0	0	0
9489	9NN2.00	under care of community alcohol team	1	0	0	0	0
65754	C150500	alcohol-induced pseudo-cushing's syndrome	1	0	0	0	0
16237	E01..00	alcoholic psychoses	1	0	0	0	0
16225	E010.00	alcohol withdrawal delirium	1	curr	0	0	0
22277	E010.11	dts - delirium tremens	1	curr	0	0	0
1476	E010.12	Delirium tremens	1	curr	0	0	0
20762	E011.00	alcohol amnestic syndrome	1	0	0	0	0
4500	E011000	korsakov's alcoholic psychosis	1	0	0	0	0
11106	E011100	korsakov's alcoholic psychosis with peripheral neuritis	1	0	0	0	0
18636	E011200	wernicke-korsakov syndrome	1	0	0	0	0
41920	E011z00	alcohol amnestic syndrome nos	1	0	0	0	0
54505	E012.00	other alcoholic dementia	1	0	0	0	0
27342	E012.11	alcoholic dementia nos	1	0	0	0	0
37946	E012000	chronic alcoholic brain syndrome	1	0	0	0	0
25110	E013.00	alcohol withdrawal hallucinosis	1	curr	0	0	0
57939	E014.00	pathological alcohol intoxication	1	curr	0	0	0
20407	E014.11	drunkenness - pathological	1	curr	0	0	0
30404	E015.00	alcoholic paranoia	1	curr	0	0	0
33670	E01y.00	other alcoholic psychosis	1	0	0	0	0
2082	E01y000	alcohol withdrawal syndrome	1	curr	0	0	0
68111	E01yz00	other alcoholic psychosis nos	1	0	0	0	0
67651	E01z.00	alcoholic psychosis nos	1	0	0	0	0
27414	E040.00	non-alcoholic amnestic syndrome	0	0	0	0	0
23835	E040.11	korsakoff's non-alcoholic psychosis	0	0	0	0	0

For peer review only

2084	E23..00	alcohol dependence syndrome	1	curr	0	0	0
2081	E23..11	alcoholism	1	curr	0	0	0
1399	E23..12	alcohol problem drinking	1	curr	H	0	0
5740	E230.00	acute alcoholic intoxication in alcoholism	1	curr	0	0	0
57714	E230.11	alcohol dependence with acute alcoholic intoxication	1	curr	0	0	0
40530	E230000	acute alcoholic intoxication, unspecified, in alcoholism	1	curr	0	0	0
56947	E230100	continuous acute alcoholic intoxication in alcoholism	1	curr	0	0	0
21624	E230200	episodic acute alcoholic intoxication in alcoholism	1	curr	0	0	0
59574	E230300	acute alcoholic intoxication in remission, in alcoholism	1	0	0	0	0
36296	E230z00	acute alcoholic intoxication in alcoholism nos	1	curr	0	0	0
31443	E231.00	chronic alcoholism	1	curr	0	0	0
37605	E231.11	Dipsomania	1	curr	0	0	0
43193	E231000	unspecified chronic alcoholism	1	curr	0	0	0
24064	E231100	continuous chronic alcoholism	1	curr	0	0	0
26106	E231200	episodic chronic alcoholism	1	curr	0	0	0
24485	E231300	chronic alcoholism in remission	1	0	0	0	0
33635	E231z00	chronic alcoholism nos	1	curr	0	0	0
6169	E23z.00	alcohol dependence syndrome nos	1	curr	0	0	0
7746	E250.00	nondependent alcohol abuse	1	curr	H	0	0
12271	E250.11	Drunkenness NOS	1	curr	0	0	0
27518	E250.12	hangover (alcohol)	1	curr	0	0	0
17777	E250.13	Inebriety NOS	1	curr	0	0	0
3782	E250.14	intoxication - alcohol	1	curr	0	0	0
669	E250000	nondependent alcohol abuse, unspecified	1	curr	H	0	0
23610	E250100	nondependent alcohol abuse, continuous	1	curr	H	0	0
12974	E250200	nondependent alcohol abuse, episodic	1	curr	H	0	0
31569	E250300	nondependent alcohol abuse in remission	1	0	0	0	0
28150	E250z00	nondependent alcohol abuse nos	1	curr	H	0	0
57993	Eu03.11	[x]korsakov's psychosis, nonalcoholic	0	0	0	0	0
7389	Eu04.12	[x]acute / subacute confusional state, nonalcoholic	0	0	0	0	0
5611	Eu10.00	[x]mental and behavioural disorders due to use of alcohol	1	0	0	0	0
44299	Eu10000	[x]mental & behav dis due to use alcohol: acute intoxication	1	curr	0	0	0
9508	Eu10011	[x]acute alcoholic drunkenness	1	curr	0	0	0

For peer review only

21879	Eu10100	[x]mental and behav dis due to use of alcohol: harmful use	1	curr	0	0	0
39327	Eu10200	[x]mental and behav dis due to use alcohol: dependence syndr	1	curr	0	0	0
28780	Eu10211	[x]alcohol addiction	1	curr	0	0	0
5758	Eu10212	[x]chronic alcoholism	1	curr	0	0	0
20514	Eu10300	[x]mental and behav dis due to use alcohol: withdrawal state	1	curr	0	0	0
64101	Eu10400	[X]Men & behav dis due alcohl: withdrawl state with delirium	1	curr	0	0	0
17259	Eu10411	[x]delirium tremens, alcohol induced	1	curr	0	0	0
12353	Eu10500	[x]mental & behav dis due to use alcohol: psychotic disorder	1	0	0	0	0
6467	Eu10511	[x]alcoholic hallucinosis	1	curr	0	0	0
65932	Eu10512	[x]alcoholic jealousy	1	curr	0	0	0
30162	Eu10513	[x]alcoholic paranoia	1	curr	0	0	0
17607	Eu10514	[x]alcoholic psychosis nos	1	0	0	0	0
39799	Eu10600	[x]mental and behav dis due to use alcohol: amnesic syndrome	1	0	0	0	0
11670	Eu10611	[x]korsakov's psychosis, alcohol induced	1	0	0	0	0
62000	Eu10700	[X]Men & behav dis due alcoh: resid & late-onset psychot dis	1	0	0	0	0
26323	Eu10711	[x]alcoholic dementia nos	1	0	0	0	0
37691	Eu10712	[x]chronic alcoholic brain syndrome	1	0	0	0	0
32927	Eu10800	[x]alcohol withdrawal-induced seizure	1	curr	0	0	0
45169	Eu10y00	[x]men & behav dis due to use alcohol: oth men & behav dis	1	0	0	0	0
64389	Eu10z00	[x]ment & behav dis due use alcohol: unsp ment & behav dis	1	0	0	0	0
36748	F11x011	alcoholic encephalopathy	1	0	0	0	0
33839	F144000	cerebellar ataxia due to alcoholism	1	0	0	0	0
30604	F25B.00	alcohol-induced epilepsy	1	0	0	0	0
2925	F375.00	alcoholic polyneuropathy	1	0	0	0	0
31742	F394100	alcoholic myopathy	1	0	0	0	0
4915	G555.00	alcoholic cardiomyopathy	1	0	0	0	0
8363	G852300	oesophageal varices in alcoholic cirrhosis of the liver	1	0	0	0	0
4506	J153.00	alcoholic gastritis	1	0	0	0	0
10691	J610.00	alcoholic fatty liver	1	0	0	0	0
3216	J611.00	acute alcoholic hepatitis	1	0	0	0	0
4743	J612.00	alcoholic cirrhosis of liver	1	0	0	0	0
21713	J612000	alcoholic fibrosis and sclerosis of liver	1	0	0	0	0
7885	J613.00	alcoholic liver damage unspecified	1	0	0	0	0

For peer review only

17330	J613000	alcoholic hepatic failure	1	0	0	0	0
16725	J615.00	cirrhosis - non alcoholic	0	0	0	0	0
16455	J615z00	non-alcoholic cirrhosis nos	0	0	0	0	0
7943	J617.00	alcoholic hepatitis	1	0	0	0	0
7602	J617000	chronic alcoholic hepatitis	1	0	0	0	0
42843	J61y.00	other non-alcoholic chronic liver disease	0	0	0	0	0
10234	J61y100	non-alcoholic fatty liver	0	0	0	0	0
98148	J61y800	nonalcoholic steatohepatitis	0	0	0	0	0
33597	J61yz00	other non-alcoholic chronic liver disease nos	0	0	0	0	0
104611	J670800	alcohol-induced acute pancreatitis	1	0	0	0	0
24984	J671000	alcohol-induced chronic pancreatitis	1	0	0	0	0
66019	L254.11	suspect fetal damage from maternal alcohol	1	0	0	0	0
27670	L255300	maternal care for (suspected) damage to fetus from alcohol	1	0	0	0	0
23945	PK80.00	fetal alcohol syndrome	0	0	0	0	0
66699	PK83.00	fetus and newborn affected by maternal use of alcohol	0	0	0	0	0
72757	Q007100	fetus/neonate affected by placental/breast transfer alcohol	1	0	0	0	0
97916	Q007111	fetal alcohol syndrome	1	0	0	0	0
9169	R103.00	[d]alcohol blood level excessive	1	0	0	0	0
36687	SLH3.00	alcohol deterrent poisoning	1	0	0	0	0
19217	SM0..00	alcohol causing toxic effect	1	0	0	0	0
8984	SM00.00	ethyl alcohol causing toxic effect	1	0	0	0	0
48514	SM00100	denatured alcohol causing toxic effect	1	0	0	0	0
55536	SM00z00	ethyl alcohol causing toxic effect nos	1	0	0	0	0
88997	SM01.00	methyl alcohol causing toxic effect	1	0	0	0	0
11263	SM01100	wood alcohol causing toxic effect	1	0	0	0	0
106018	SM02.00	isopropyl alcohol causing toxic effect	1	0	0	0	0
102086	SM02200	rubbing alcohol causing toxic effect	1	0	0	0	0
64396	SM03000	amyl alcohol causing toxic effect	1	0	0	0	0
102321	SM03100	butyl alcohol causing toxic effect	1	0	0	0	0
101543	SM0y.00	other alcohol causing toxic effect	1	0	0	0	0
36499	SM0z.00	alcohol causing toxic effect nos	1	0	0	0	0
95410	SyuG000	[x]toxic effect of other alcohols	1	0	0	0	0
18252	T90..00	accidental poisoning by alcohol, nec	1	0	0	0	0

For peer review only

40541	T900.00	accidental poisoning by alcoholic beverages	1	0	0	0	0
15503	T901.00	accidental poisoning by other ethyl alcohol and its products	1	0	0	0	0
101426	T901z00	accidental poisoning by ethyl alcohol nos	1	0	0	0	0
37174	T902.00	accidental poisoning by methyl alcohol	1	0	0	0	0
60752	T903300	accidental poisoning by secondary propyl alcohol	1	0	0	0	0
53139	T90y.00	accidental poisoning by other alcohols	1	0	0	0	0
55415	T90z.00	accidental poisoning by alcohol nos	1	0	0	0	0
21412	TJH3.00	adverse reaction to alcohol deterrents	1	0	0	0	0
31605	U1A9.00	[x]accident poisoning/exposure to alcohol	1	0	0	0	0
63306	U1A9000	[x]accident poison/exposure to alcohol at home	1	0	0	0	0
103069	U1A9200	[x]acc poison/expos alcohol school/pub admin area	1	0	0	0	0
104702	U1A9300	[x]accid pois/expos alcohol in sport/athletic area	1	0	0	0	0
63876	U1A9400	[x]accid poison/expos alcohol in street/highway	1	0	0	0	0
92908	U1A9500	[x]accid poison/expos alcohol trade/service area	1	0	0	0	0
104734	U1A9y00	[x]accid pois/expos to alcohol other spec place	1	0	0	0	0
61187	U1A9z00	[x]accid poison/expos to alcohol unspecif place	1	0	0	0	0
10463	U209.00	[x]intent self poison/exposure to alcohol	1	0	0	0	0
41638	U209000	[x]int self poison/exposure to alcohol at home	1	0	0	0	0
97085	U209y00	[x]int self poison alcohol other spec place	1	0	0	0	0
59414	U209z00	[x]intent self poison alcohol unspecif place	1	0	0	0	0
30036	U409.00	[x]poisoning/exposure, ? intent, to alcohol	1	0	0	0	0
68159	U409000	[x]poison/exposure ?intent, to alcohol at home	1	0	0	0	0
69407	U409200	[x]pois/exp ?intent alcohol school/pub admin area	1	0	0	0	0
96219	U409400	[x]pois/expos ?intent alcohol in street/highway	1	0	0	0	0
61190	U409z00	[x]pois/expos ?intent to alcohol unspecif place	1	0	0	0	0
73876	U60H300	[x]alcohol deterrents caus adverse effects in therapeut use	1	0	0	0	0
48241	U60H311	[x] adverse reaction to alcohol deterrents	1	0	0	0	0
44019	U80..00	[x]evidence of alcohl involv determin by blood alcohl level	1	0	0	0	0
12950	U800.00	[x]eviden of alcohl involv blood alcohl level <20 mg/100 ml	1	0	0	0	0
57202	U801.00	[x]eviden of alcohl involv blood alcohl level 20-39mg/100ml	1	0	0	0	0
59079	U802.00	[x]eviden of alcohl involv blood alcohl level 40-59mg/100ml	1	0	0	0	0
57242	U803.00	[x]eviden of alcohl involv blood alcohl level 60-79mg/100ml	1	0	0	0	0
53428	U804.00	[x]eviden of alcohl involv blood alcohl level 80-99mg/100ml	1	0	0	0	0

For peer review only - http://bmjopen.bmj.com/site/about/guidelines.xhtml

54504	U805.00	[x]eviden of alcoh involv blood alcoh level 100-119mg/100ml	1	0	0	0	0
32454	U806.00	[x]eviden of alcoh involv blood alcoh level 120-199mg/100ml	1	0	0	0	0
47907	U807.00	[x]eviden of alcoh involv blood alcoh level 200-239mg/100ml	1	0	0	0	0
39726	U808.00	[x]eviden alcoh involv blood alcoh level 240mg/100ml or more	1	0	0	0	0
103698	U80z.00	[x]evid alcoh invol detrm by pres alcoh in bld levl not spec	1	0	0	0	0
23978	U81..00	[x]evid of alcohol involv determind by level of intoxication	1	0	0	0	0
105273	U810.00	[x]evid alcoh invol determ by levl of intox mild alcoh intox	1	0	0	0	0
73480	U811.00	[x]evid alcoh invol determ by level of intox mod alcoh intox	1	0	0	0	0
44686	U812.00	[x]evid alcoh invol determ by level of intox sev alcoh intox	1	0	0	0	0
39738	U813.00	[x]evid alcoh invl determ by levl intox very sev alcoh intox	1	0	0	0	0
41983	Z191.00	alcohol detoxification	1	0	0	0	0
46677	Z191100	alcohol withdrawal regime	1	0	0	0	0
61383	Z191200	planned reduction of alcohol consumption	1	curr	0	0	0
95181	Z191211	alcohol reduction programme	1	curr	0	0	0
64409	Z191400	self-monitoring of alcohol intake	1	0	0	0	0
30460	Z4B1.00	alcoholism counselling	1	curr	0	0	0
21829	Z786200	drinking practice	1	0	0	0	0
95650	ZC22100	advice to change drink intake	1	curr	0	0	0
97163	ZC22200	advice to change alcoholic drink intake	1	curr	0	0	0
54209	ZC2H.00	advice to change alcohol intake	1	curr	0	0	0
104109	ZC67111	Drinks only	0	0	0	0	0
11140	ZG23100	advice on alcohol consumption	1	0	0	0	0
50507	ZR1E.00	alcohol dependence scale	1	0	0	0	0
40602	ZR1E.11	ads - alcohol dependence scale	1	0	0	0	0
32850	ZR1F.00	alcohol use disorders identification test	1	0	0	1	0
59873	ZR1F.11	audit - alcohol use disorders identification test	1	0	0	1	0
65980	ZR1G.00	alcohol use inventory	1	0	0	0	0
45557	ZR31.00	cage questionnaire	1	0	0	0	0
61750	ZR3f.00	comprehensive drinker profile	1	0	0	0	0
63457	ZR3f.11	cdp - comprehensive drinker profile	1	0	0	0	0
56441	ZRa1.00	michigan alcoholism screening test	1	0	0	0	0
69331	ZRa1.11	mast - michigan alcoholism screening test	1	0	0	0	0
99985	ZRa1100	brief michigan alcoholism screening test	1	0	0	0	0

For peer review only

41251	ZRa1111	bmast - brief michigan alcoholism screening test	1	0	0	0	0
70939	ZRaU.00	munich alcoholism test	1	0	0	0	0
22707	ZRBJ.00	drinking problem scale	1	0	0	0	0
46848	ZRBJ.11	dps - drinking problem scale	1	0	0	0	0
42305	ZRk6.00	severity of alcohol dependence questionnaire	1	0	0	0	0
66831	ZRk6.11	sadq - severity of alcohol dependence questionnaire	1	0	0	0	0
62300	ZRk9.00	short alcohol dependence data	1	0	0	0	0
62299	ZRk9.11	sadd - short alcohol dependence data	1	0	0	0	0
10658	ZRLfD12	honos item 3 - alcohol/drug problem	1	0	0	0	0
66552	ZRR..00	inventory of drinking situations	1	0	0	0	0
59776	ZRVK.00	last six months of drinking questionnaire	1	0	0	0	0
7123	ZV11300	[v]personal history of alcoholism	1	0	0	0	0
16587	ZV11311	[v]problems related to lifestyle alcohol use	1	curr	H	0	0
7545	ZV4KC00	[v] alcohol use	1	0	0	0	0
8388	ZV57A00	[v]alcohol rehabilitation	1	0	0	0	0
8030	ZV6D600	[v]alcohol abuse counselling and surveillance	1	curr	0	0	0
61583	ZV70411	[v]medicolegal blood alcohol test	1	0	0	0	0
17149	ZV70L00	[v]blood-alcohol and blood-drug test	1	0	0	0	0
10458	ZV79100	[v]screening for alcoholism	1	0	0	0	0

For peer review only

Appendix Table 2. Internal validity comparing alcohol consumption recorded up to 30 days before or after an AUDIT score or measure of units per week.

	N	(%)	N patients with AUDIT score on the same date as record n	(%)	AUDIT score median	(IQR)	N patients with units per week recorded on same date as record n	(%)	Units per week median	(IQR)
**Current drinking status***										
Non	175,022	(20.3)	3440	(2)	0	(0-0)	913	(0.5)	0	(0-1)
Ex	31,525	(3.7)	348	(1.1)	0	(0-0)	999	(3.2)	0	(0-1)
Current	655,783	(76)	25782	(3.9)	3	(1-5)	333,371	(50.8)	6	(2-14)
**Current drinking level (Read codes)**										
Non drinker	222,830	(39.9)	3,856	(1.7)	0	(0-0)	13,033	(25)	0	(0-2)
Light drinker	255,922	(45.8)	3,767	(1.5)	2	(1-3)	19,767	(7.7)	3	(1-8)
Moderate drinker	52,066	(9.3)	1,300	(2.5)	1	(0-4)	13,033	(25)	6	(2-14)
Heavy drinker	27,929	(5)	2,849	(10.2)	6	(5-9)	10,570	(37.8)	18	(10-30)
**Units per week**										
0	38,917	(9)	1,941	(5)	0	(0-0)	n/a		n/a	
1-14	298,463	(69.2)	12,734	(4.3)	3	(2-4)				
15-42	80,434	(18.6)	2,988	(3.7)	6	(4-8)				
43+	13,580	(3.1)	241	(1.8)	11	(8-12)				
**AUDIT score category**										
0-7; Alcohol Education	79,764	(92.8)	n/a		n/a		16,859	(21.1)	4	(2-10)
8-15; Simple Advice	5,752	(6.7)					1,604	(27.9)	20	(12-30)
16-19; Simple Advice plus Brief Counselling and Continued Monitoring	194	(0.2)					38	(19.6)	40	(20-60)
20-40; Referral to Specialist for Diagnostic Evaluation and Treatment	213	(0.2)					30	(14.1)	60	(25-88)

*based on recorded status in Read codes and data captured through structured data areas in GP software (i.e. included in the Additional data file) (not including derived status based on units consumed or AUDIT score)

For peer review only

References

1. Denaxas, S.C., et al., *Data resource profile: cardiovascular disease research using linked bespoke studies and electronic health records (CALIBER).* Int J Epidemiol, 2012. **41**(6): p. 1625-38.
2. Department of Health, *UK Chief Medical Officers' alcohol guidelines review: Summary of the proposed new guidelines.* 2016.
3. Babor, T.F., et al. *The Alcohol Use Disorders Identification Test: Guidelines for Use in Primary Care*. 2001; Available from: http://www.talkingalcohol.com/files/pdfs/WHO_audit.pdf.

For peer review only

ISAC APPLICATION FORM

PROTOCOLS FOR RESEARCH USING THE CLINICAL PRACTICE RESEARCH DATALINK (CPRD)

For ISAC use only		
Protocol No.	....	***IMPORTANT***
Submission date (DD/MM/YYYY)	....	*Please refer to the **guidance** for '**Completing the ISAC application form'** found on the CPRD website (www.cprd.com/isac). If you have any queries, please contact the ISAC Secretariat at isac@cprd.com.*

SECTION A: GENERAL INFORMATION ABOUT THE PROPOSED RESEARCH STUDY

1. Study Title§ (*Please state the study title below)*

Variation in recording of alcohol use in CPRD: a cross-sectional study

§Please note: This information will be published on the CPRD's website as part of its transparency policy.

2. Has any part of this research proposal or a related proposal been previously submitted to ISAC?

Yes* ☐ No ☒

**If yes, please provide the previous protocol number/s below. Please also state in your current submission how this/these are related or relevant to this study.*

3. Has this protocol been peer reviewed by another Committee? (e.g. grant award or ethics committee)

Yes* ☐ No ☒

**If Yes, please state the name of the reviewing Committee(s) below and provide an outline of the review process and outcome as an Appendix to this protocol :*

4. Type of Study (please tick all the relevant boxes which apply)

Adverse Drug Reaction/Drug Safety	☐	Drug Effectiveness	☐
Drug Utilisation	☐	Pharmacoeconomics	☐
Disease Epidemiology	☐	Post-authorisation Safety	☐
Health care resource utilisation	☐	Methodological Research	☒
Health/Public Health Services Research	☐	Other*	☐

**If Other, please specify the type of study here and in the lay summary below:*

5. Health Outcomes to be Measured§

§Please note: This information will be published on CPRD's website as part of its transparency policy.

Please summarise below the primary/secondary health outcomes to be measured in this research protocol:

- Alcohol use recording:
  - Alcohol screening using recommended tools (AUDIT and FAST)
  - Alcohol units per week
  - Other alcohol screening recorded using morbidity codes

For peer review only

6. Publication: This study is intended for (please tick all the relevant boxes which apply)**:**

Publication in peer-reviewed journals	☒	Presentation at scientific conference	☐
Presentation at company/institutional meetings	☐	Regulatory purposes	☐
Other*	☐		

**If Other, please provide further information:*

SECTION B: INFORMATION ON INVESTIGATORS AND COLLABORATORS

7. Chief Investigator§
Please state the full name, job title, organisation name & e-mail address for correspondence - see guidance notes for eligibility. Please note that there can only be one Chief Investigator per protocol.

Dr Emily Herrett, Assistant Professor, London School of Hygiene & Tropical Medicine
Email: emily.herrett@lshtm.ac.uk

§Please note: The name and organisation of the Chief Investigator and will be published on CPRD's website as part of its transparency policy

CV has been previously submitted to ISAC ☒ **CV number:** 085_15
A new CV is being submitted with this protocol ☐
An updated CV is being submitted with this protocol ☐

8. Affiliation of Chief Investigator (full address)

Department of Non-communicable Disease Epidemiology, Faculty of Epidemiology and Population Health, London School of Hygiene and Tropical Medicine, Keppel Street, London, WC1E 7HT.

9. Corresponding Applicant§
Please state the full name, affiliation(s) and e-mail address below:

Emily Herrett, Department of Non-communicable Disease Epidemiology, Faculty of Epidemiology & Population Health, London School of Hygiene & Tropical Medicine
Email: Emily.herrett@lshtm.ac.uk

§Please note: The name and organisation of the corresponding applicant and their organisation name will be published on CPRD's website as part of its transparency policy

Same as chief investigator ☐
CV has been previously submitted to ISAC ☒ **CV number:** 353_16S
A new CV is being submitted with this protocol ☐
An updated CV is being submitted with this protocol ☐

10. List of all investigators/collaborators§
Please list the full name, affiliation(s) and e-mail address* of all collaborators, other than the Chief Investigator below:

§Please note: The name of all investigators and their organisations/institutions will be published on CPRD's website as part of its transparency policy

Other investigator: Sarah Cook, Department of Non-communicable Disease Epidemiology, Faculty of Epidemiology & Population Health, London School of Hygiene & Tropical Medicine
Email: sarah.cook@lshtm.ac.uk
CV has been previously submitted to ISAC ☐ **CV number:**
A new CV is being submitted with this protocol ☒
An updated CV is being submitted with this protocol ☐

Other investigator: Kathryn Mansfield, Department of Non-communicable Disease Epidemiology, Faculty of Epidemiology & Population Health, London School of Hygiene & Tropical Medicine

For peer review only - http://bmjopen.bmj.com/site/about/guidelines.xhtml

Email: kathryn.mansfield@lshtm.ac.uk
CV has been previously submitted to ISAC ☒ **CV number:** 157_15CESL
A new CV is being submitted with this protocol ☐
An updated CV is being submitted with this protocol ☐

Other investigator: Jennifer Quint, National Heart and Lung Institute, Imperial College London
Email: j.quint@imperial.ac.uk
CV has been previously submitted to ISAC ☒ **CV number:** 042_15CEPSL
A new CV is being submitted with this protocol ☐
An updated CV is being submitted with this protocol ☐

Other investigator: Rachel Denholm, School of Social and Community Medicine, University of Bristol
Email: r.denholm@bristol.ac.uk
CV has been previously submitted to ISAC ☒ **CV number:** 631_16C
A new CV is being submitted with this protocol ☐
An updated CV is being submitted with this protocol ☐

Other investigator: Liam Smeeth, Department of Non-communicable Disease Epidemiology, Faculty of Epidemiology & Population Health, London School of Hygiene and Tropical Medicine
Email: liam.smeeth@lshtm.ac.uk
CV has been previously submitted to ISAC ☒ **CV number:** 045_15CEPSL
A new CV is being submitted with this protocol ☐
An updated CV is being submitted with this protocol ☐

Please note that your ISAC application form and protocol **must be copied to all e-mail addresses listed above at the time of submission of your application to the ISAC mailbox. Failure to do so will result in delays in the processing of your application.*

11. Conflict of interest statement*

Please provide a draft of the conflict (or competing) of interest (COI) statement that you intend to include in any publication which might result from this work

The authors declare no conflict of interest for the submitted work; no financial relationships with any organisations that might have an interest in the submitted work in the previous three years; no other relationships or activities that could appear to have influenced the submitted work.

**Please refer to the International Committee of Medical Journal Editors (ICMJE) for guidance on what constitutes a COI.*

12. Experience/expertise available

Please complete the following questions to indicate the experience/ expertise available within the team of investigators/collaborators actively involved in the proposed research, including the analysis of data and interpretation of results.

	Previous GPRD/CPRD Studies	Publications using GPRD/CPRD data
None	☐	☐
1-3	☐	☐
> 3	☒	☒

Experience/Expertise available	Yes	No
**Is statistical expertise available within the research team?**<br>*If yes, please indicate the name(s) of the relevant investigator(s)*<br>Sarah Cook	☒	☐
**Is experience of handling large data sets (>1 million records) available within the research team?**<br>*If yes, please indicate the name(s) of the relevant investigator(s)*<br>Elizabeth Crellin, Emily Herrett, Kathryn Mansfield	☒	☐

For peer review only - http://bmjopen.bmj.com/site/about/guidelines.xhtml

**Is experience of practising in UK primary care available to or within the research team?**<br>*If yes, please indicate the name(s) of the relevant investigator(s)*<br>Liam Smeeth	☒	☐

13. References relating to your study

Please list up to 3 references (most relevant) relating to your proposed study:

1. NICE. Alcohol-use disorders: prevention. NICE guidelines PH24. (2010).
2. Babor, T. F., Higgins-Biddle, J. C., Saunders, J. B. & Monteiro, M. G. *The Alcohol Use Disorders Identification Test: Guidelines for Use in Primary Care*, <http://www.talkingalcohol.com/files/pdfs/WHO_audit.pdf> (2001).
3. Otete, H. E., Orton, E., West, J. & Fleming, K. M. Sex and age differences in the early identification and treatment of alcohol use: a population-based study of patients with alcoholic cirrhosis. *Addiction (Abingdon, England)* **110**, 1932-1940, doi:10.1111/add.13081 (2015).

SECTION C: ACCESS TO THE DATA

14. Financial Sponsor of study§

§*Please note: The name of the source of funding will be published on CPRD's website as part of its transparency policy*

Pharmaceutical Industry	☐	*Please specify name and country:*
Academia	☐	*Please specify name and country:*
Government / NHS	☒	*Please specify name and country:* NIHR, UK
Charity	☒	*Please specify name and country:* Wellcome Trust, UK
Other	☐	*Please specify name and country:*
None	☐	

15. Type of Institution conducting the research

Pharmaceutical Industry	☐	*Please specify name and country:*
Academia	☒	*Please specify name and country:* LSHTM, UK
Government Department	☐	*Please specify name and country:*
Research Service Provider	☐	*Please specify name and country:*
NHS	☐	*Please specify name and country:*
Other	☐	*Please specify name and country:*

16. Data access arrangements

The financial sponsor/ collaborator* has a licence for CPRD GOLD and will extract the data	☐
The institution carrying out the analysis has a licence for CPRD GOLD and will extract the data**	☒
A data set will be provided by the CPRD¥€	☐
CPRD has been commissioned to extract the data <u>and</u> perform the analyses€	☐
Other:	☐

If Other, please specify:

**Collaborators supplying data for this study must be named on the protocol as co-applicants.*
***If data sources other than CPRD GOLD are required, these will be supplied by CPRD*
¥*Please note that datasets provided by CPRD are limited in size; applicants should contact CPRD (enquiries@cprd.com) if a dataset of >300,000 patients is required.*
€*Investigators must discuss their request with a member of the CPRD Research team before submitting an ISAC application. Please contact the CPRD Research Team on +44 (20) 3080 6383 or email (enquiries@cprd.com) to discuss your requirements. Please also state the name of CPRD Research team with whom you have discussed this request (provide the date of discussion and any relevant reference information):*

Name of CPRD Researcher	Reference number (where available)	Date of contact

17. Primary care data

Please specify which primary care data set(s) are required)

Vision only (Default for CPRD studies	☒	Both Vision and EMIS®*	☐
EMIS® only*	☐		

Note: Vision and EMIS are different practice management systems. CPRD has traditionally collected data from Vision practice. Data collected from EMIS is currently under evaluation prior to wider release.

For peer review only - http://bmjopen.bmj.com/site/about/guidelines.xhtml

Investigators requiring the use of EMIS data **must discuss the study with a member of the CPRD Research team before submitting an ISAC application*

Please state the name of the CPRD Researcher with whom you have discussed your request for EMIS data:
Name of CPRD Researcher Reference number (where available) Date of contact

SECTION D: INFORMATION ON DATA LINKAGES

18. Does this protocol seek access to linked data

Yes* ☒ No☐ If No, please move to section E.

Research groups which have not previously accessed CPRD linked data resources **must discuss access to these resources with a member of the CPRD Research team, before submitting an ISAC application. Investigators requiring access to HES Accident and Emergency data, HES Diagnostic Imaging Dataset PROMS data and the Pregnancy Register **must** also discuss this with a member of the CPRD Research team before submitting an ISAC application. Please contact the CPRD Research Team on +44 (20) 3080 6383 or email enquiries@cprd.comto discuss your requirements **before** submitting your application.*

Please state the name of the CPRD Researcher with whom you have discussed your linkage request.

Name of CPRD Researcher: Rebecca Ghosh
Reference number (where available): CPRD00007105
Date of contact: 13 June 2017

Please note that as part of the ISAC review of linkages, your protocol may be shared - in confidence - with a representative of the requested linked data set(s) and summary details may be shared - in confidence - with the Confidentiality Advisory Group of the Health Research Authority.

19. Please select the source(s) of linked data being requested§
§Please note: This information will be published on the CPRD's website as part of its transparency policy.

☐ ONS Death Registration Data
☐ HES Admitted Patient Care
☐ HES Outpatient
☐ HES Accident and Emergency
☐ HES Diagnostic Imaging Dataset
☐ MINAP (Myocardial Ischaemia National Audit Project)
☐ Cancer Registration Data*
☐ PROMS (Patient Reported Outcomes Measure)**
☐ CPRD Mother Baby Link
☐ Pregnancy Register

☐ Practice Level Index of Multiple Deprivation (Standard)
☐ Practice Level Index of Multiple Deprivation (Bespoke)
☒ Patient Level Index of Multiple Deprivation***
☐ Patient Level Townsend Score ***
☐ Other**** *Please specify:*

**Applicants seeking access to cancer registration data must complete a Cancer Dataset Agreement form (available from CPRD). This should be submitted to the ISAC as an appendix to your protocol. Please also note that applicants seeking access to cancer registry data must provide consent for publication of their study title and study institution on the UK Cancer Registry website.*
***Assessment of the quality of care delivered to NHS patients in England undergoing four procedures: hip replacement, knee replacement, groin hernia and varicose veins. Please note that patient level PROMS data are only accessible by academics*
**** 'Patient level IMD and Townsend scores will not be supplied for the same study*
*****If "Other" is specified, please provide the name of the individual in the CPRD Research team with whom this linkage has been discussed.*

Name of CPRD Researcher Reference number (where available) Date of contact

20. Total number of linked datasets requested <u>including</u> CPRD GOLD

Number of linked datasets requested *(practice/ 'patient' level Index of Multiple Deprivation, Townsend Score, the CPRD Mother Baby Link and the Pregnancy Register should **not** be included in this count)* 1

Please note: Where ≥5 linked datasets are requested, approval may be required from the Confidentiality Advisory Group (CAG) to access these data

21. Is linkage to a <u>local</u>¥ dataset with <1 million patients being requested?

For peer review only - http://bmjopen.bmj.com/site/about/guidelines.xhtml

Yes* ☐ No ☒

*If yes, please provide further details:
¥ Data from defined geographical areas i.e. non-national datasets.

22. If you have requested one or more linked data sets, please indicate whether the Chief Investigator or any of the collaborators listed in question 5 above, have access to these data in a patient identifiable form (e.g. full date of birth, NHS number, patient post code), or associated with an identifiable patient index.

Yes* ☐ No ☒

* If yes, please provide further details:

23. Does this study involve linking to patient *identifiable* data (e.g. hold date of birth, NHS number, patient post code) from other sources?

Yes ☐ No ☒

SECTION E: VALIDATION/VERIFICATION

24. Does this protocol describe a purely observational study using CPRD data?

Yes* ☒ No** ☐

** Yes: If you will be using data obtained from the CPRD Group, this study does not require separate ethics approval from an NHS Research Ethics Committee.*
*** No: You may need to seek separate ethics approval from an NHS Research Ethics Committee for this study. The ISAC will provide advice on whether this may be needed.*

25. Does this protocol involve requesting any additional information from GPs?

Yes* ☐ No ☒

* If yes, please indicate what will be required:

Completion of questionnaires by the GPᵠ	Yes ☐	No ☐
Is the questionnaire a validated instrument?	Yes ☐	No ☐
If yes, has permission been obtained to use the instrument?	Yes ☐	No ☐
Please provide further information:		

Other (please describe)

ᵠ Any questionnaire for completion by GPs or other health care professional must be approved by ISAC before circulation for completion.

26. Does this study require contact with patients in order for them to complete a questionnaire?

Yes* ☐ No ☒

Please note that any questionnaire for completion by patients must be approved by ISAC before circulation for completion.

27. Does this study require contact with patients in order to collect a sample?

Yes* ☐ No ☒

* Please state what will be collected:

SECTION F: DECLARATION

For peer review only - http://bmjopen.bmj.com/site/about/guidelines.xhtml

28. Signature from the Chief Investigator

- I have read the guidance on '***Completion of the ISAC application form***' and '***Contents of CPRD ISAC Research Protocols***' and have understood these;
- I have read the submitted version of this research protocol, including all supporting documents, and confirm that these are accurate.
- I am suitably qualified and experienced to perform and/or supervise the research study proposed.
- I agree to conduct or supervise the study described in accordance with the relevant, current protocol
- I agree to abide by all ethical, legal and scientific guidelines that relate to access and use of CPRD data for research
- I understand that the details provided in sections marked with (§) in the application form and protocol will be published on the CPRD website in line with CPRD's transparency policy.
- I agree to inform the CPRD of the final outcome of the research study: publication, prolonged delay, completion or termination of the study.

Name: Emily Herrett Date: 11/08/17 e-Signature (type name): Emily Herrett

For peer review only

For peer review only - http://bmjopen.bmj.com/site/about/guidelines.xhtml

PROTOCOL INFORMATION REQUIRED

The following sections below **must** be included in the CPRD ISAC research protocol. Please refer to the guidance on '***Contents of CPRD ISAC Research Protocols***' (www.cprd.com/isac) for more information on how to complete the sections below. Pages should be numbered. All abbreviations must be defined on first use.

Applicants must complete all sections listed below
Sections which do not apply should be completed as '*Not Applicable*'

A. Study Title§

§*Please note: This information will be published on CPRD's website as part of its transparency policy*

Variation in recording of alcohol use in CPRD: a cross-sectional study

B. Lay Summary (Max. 200 words)§

§*Please note: This information will be published on CPRD's website as part of its transparency policy*

Hazardous alcohol use is a key behaviour risk factor that affects health. Routine alcohol screening for adults is recommended by the National Institute for Health and Care Excellence (NICE) to help prevent alcohol-use disorders. NICE recommends using the Alcohol Use Disorders Identification Test (AUDIT), a screening questionnaire. General practitioners should be carrying out alcohol screening as an integral part of their practice. Little is known about how many patients are screened and whether alcohol use is being recorded accurately in general practice. In this study we will use data from the Clinical Practice Research Datalink (CPRD), a database of routinely collected medical record data from general practitioners in the UK, to find out how many patients had a record of alcohol use, and whether they were screened using a recommended questionnaire. We will look at how the recording of alcohol use varies in different patient groups, such as those with particular health problems. This will show to what extent recommendations for alcohol screening are followed, and whether particular patient groups are more likely to be screened than others. This will help to guide researchers using alcohol data in future research.

C. Technical Summary (Max. 200 words)§

§*Please note: This information will be published on CPRD's website as part of its transparency policy*

Alcohol screening for adults is recommended by the National Institute for Health and Care Excellence (NICE) as an integral part of practice for the prevention of alcohol-use disorders. We will assess the utility of recorded alcohol data within the Clinical Practice Research Datalink (CPRD) by assessing prevalence and accuracy of recording of alcohol. We will determine the prevalence of screening at different levels: alcohol screening using recommended screening tools (AUDIT or FAST), alcohol units per week, or other alcohol screening using morbidity codes. We will also investigate how prevalence of recording varies with patient characteristics. We will conduct a cross-sectional study, where the study population is all people aged 16 years or over who were registered in CPRD (up-to-standard) on the study date. The study date will be chosen to allow us to use the most recent data available in CPRD. We will then validate information gathered from morbidity codes and alcohol units per week, using scores from recommended screening tools as the gold standard. This study will provide insight into how well routine practice is aligned with NICE guidelines for alcohol screening, and help establish best practise in using CPRD data to investigate alcohol use in research.

For peer review only - http://bmjopen.bmj.com/site/about/guidelines.xhtml

Applicants must complete all sections listed below
Sections which do not apply should be completed as '*Not Applicable*'

D. Objectives, Specific Aims and Rationale

Broad research aim:

The overall aim of this project is to investigate the completeness and accuracy of recording of alcohol use within primary care records in the UK.

Specific objectives:

1. To determine the prevalence of recording of any morbidity codes relating to alcohol in electronic medical records among adult patients in CPRD.

2. To determine the prevalence and frequency of: a) alcohol screening using recommended tools, b) alcohol units per week and c) other alcohol screening recorded using morbidity codes for alcohol consumption, and to investigate how prevalence of these varies with patient characteristics.

3. To validate morbidity coding for recording of level of alcohol use, using scores from recommended screening tools (AUDIT/FAST) and recorded units of alcohol per week, by comparing:
   a. Read morbidity coding and alcohol status from Clinical Additional file against AUDIT/FAST scores and units per week data, and
   b. Units per week data against AUDIT/FAST scores.

Rationale:

This study will provide insight into how well routine practice is aligned with NICE guidelines for alcohol screening in primary care, and help establish best practise in using CPRD data in research investigating alcohol use. Understanding patient- and practice-level factors that are related to whether alcohol screening is carried out and recorded appropriately will have public health benefit in identifying potential missed opportunities for intervention in particular patient groups, and benefit research by helping us understand the potential limitations and biases of alcohol use data recorded in CPRD. For example, if alcohol use is less likely to be recorded in patients with higher socio-economic status, this will affect the representativeness of any study sample restricted to patients with alcohol use recorded.

E. Study Background

Hazardous alcohol use is a key behaviour risk factor affecting health. Identification of high risk patients should take place in primary care, where appropriate interventions can also be promoted. Alcohol screening for adults is recommended by the National Institute for Health and Care Excellence (NICE) as an integral part of practice for the prevention of alcohol-use disorders.[1] NICE guidelines published in 2010 recommend that opportunistic screening of the general population should take place during new patient registrations, and when screening or managing other conditions, promoting sexual health, during antenatal appointments and treating minor injuries. In addition,

For peer review only - http://bmjopen.bmj.com/site/about/guidelines.xhtml

Applicants must complete all sections listed below
Sections which do not apply should be completed as '*Not Applicable'*

guidelines recommend screening 'high risk' groups, including those with relevant physical conditions (such as hypertension) and relevant mental health problems (such as depression).[1]

The Alcohol Use Disorders Identification Test (AUDIT) is a 10-item screening tool for harmful or hazardous alcohol consumption designed and validated for use in primary care settings.[2] The AUDIT is the main tool recommended for alcohol screening in primary care by NICE. A range of shortened versions related to AUDIT (such as AUDIT-C and FAST) are also considered acceptable tools for alcohol screening, depending on time available.[1] The NHS health check, which should be offered to all people in the UK aged 40-74 years every five years, includes the AUDIT questions within it.[3]

The Clinical Practice Research Datalink (CPRD) is an ongoing primary care database of anonymised medical records from general practitioners with coverage of 11.3 million patients from 674 practices in the UK. Little is known about the accuracy of recording of alcohol use within primary care records in the UK, both in terms of completeness of recording and the validity of recorded data, although a study of patients with alcoholic cirrhosis using CPRD data found that men were more likely than women to have their alcohol use recorded.[4]

Our study will assess the utility of recorded alcohol data in the CPRD by assessing completeness of recording of alcohol status at different levels. We will first assess the prevalence of codes relating to alcohol (whether relating to harm from alcohol use, alcohol use disorders, or management of alcohol use). Then we will determine the prevalence of screening at different levels: alcohol screening using recommended screening tools (AUDIT or FAST), alcohol units per week, or other alcohol screening using morbidity codes. We will also compare the prevalence of alcohol use recording at these different levels in patient groups with different characteristics: by age and sex, geographical region, socio-economic status, time since registration, chronic diseases associated with harmful alcohol consumption, smoking status, body mass index (BMI) and ethnicity. We will then validate information gathered from morbidity codes for alcohol use, using scores from recommended screening tools as a gold standard.

This study will provide insight into the completeness of alcohol use data in the CPRD in different patient groups, including specifically in those identified as being at higher risk according to NICE recommendations, and will provide guidance for future studies related to alcohol use using CPRD data. Further work may investigate whether alcohol use scores are followed up with appropriate interventions and whether alcohol use, when recorded appropriately, is predictive of future alcohol-related health problems.

F. Study Type

Methodological/validation study.

For peer review only - http://bmjopen.bmj.com/site/about/guidelines.xhtml

Applicants must complete all sections listed below
Sections which do not apply should be completed as '*Not Applicable*'

G. Study Design

We will use a cross-sectional study to quantify and characterise the completeness and frequency of primary care records for alcohol use within the previous five years, on a given date (the study date will be chosen based on use of the most recent data available in CPRD at the time we start the study).

H. Feasibility counts

Because we intend to determine the prevalence of recording of alcohol use in CPRD, we will include all individuals (16 years and over) registered in the CPRD (up to standard) with at least one year of research-standard follow-up, on the study date. However, we will conduct separate analyses for those aged 16-17 and those 18 years and over on the study date. Therefore, to be conservative our feasibility count is limited to individuals aged 18 years and over.

We identified 2,355,525 individuals aged 18 years and over who were registered in the CPRD (up to standard), with at least one year of research-standard follow-up, on 1st January 2016. Based on an initial feasibility count using a sample of 1 million patients from an earlier release from the CPRD, we found that 54% of individuals over 18 years had any alcohol-related record within the previous five years on 1st January 2015.

The AUDIT or FAST score is the gold standard against which we will validate alcohol Read morbidity coding, alcohol status recorded in the Additional Clinical Details file, and recorded units per week. In the feasibility analysis we found that 31% of individuals over 18 had a Read code relating to alcohol use, 51% had a record of alcohol status from the Additional Clinical Details file, 29% had a record of the number of units of alcohol drunk per week, and 12% had a recorded AUDIT or FAST score.

I. Sample size considerations

See section H (Feasibility Counts). This is a validation study aiming to determine the prevalence of recording of alcohol use across the whole CPRD population on a specific date (without exploring any associations with health outcomes), therefore there was no need for a formal power calculation.

J. Data Linkage Required (if applicable):§

§Please note that the data linkage/s requested in research protocols will be published by the CPRD as part of its transparency policy

Index of Multiple Deprivation (IMD)

We will link to patient-level IMD in order to investigate whether the completeness of alcohol use recording differs by socio-economic status.

K. Study population

The study population will include all patients 16 years or over, alive and registered with an up-to-standard CPRD practice on the study date, with at least one year of research-standard follow-up to ensure accurate recording of relevant comorbidities. The study date will be chosen based on use of the most recent data available in CPRD at

For peer review only - http://bmjopen.bmj.com/site/about/guidelines.xhtml

Applicants must complete all sections listed below
Sections which do not apply should be completed as '*Not Applicable*'

the time we start the study. Results for patients aged 16 and 17 years will be reported separately from patients aged over 18 years.

L. Selection of comparison group(s) or controls

The comparison will be between patients with and without recorded alcohol use using different code-based definitions.

M. Exposures, Health Outcomes§ and Covariates

§Please note: Summary information on health outcomes (as included on the ISAC application form above)will be published on CPRD's website as part of its transparency policy

Preliminary code lists for all variables are included in the Appendix (Excel workbook).

Exposures

We will investigate the impact of a range of potential exposures on whether alcohol use was recorded including: age, sex, socio-economic status, geographic area, comorbidities related to alcohol use (liver disease, hypertension, depression and anxiety) or those where health function monitoring is likely to be more frequent/complete (diabetes mellitus), time since registration at the current practice, BMI, smoking status, and ethnicity.

Socioeconomic status at the patient level will be categorised using quintiles of index of multiple deprivation scores for 2015 (chosen as the closest to our study date). Time since registration will be categorized as i. 1-5 years, or ii. 5+ years.

Liver disease, hypertension and diabetes mellitus will be recorded based on presence of a recorded diagnosis at any time prior to the study date. Depression and anxiety may be transient. We will therefore record them based on the presence of a diagnosis at any time within five years prior to the study date. We will conduct sensitivity analyses to test these exposure definitions, defining liver disease, hypertension and diabetes mellitus as being present if there was a diagnosis within the five years prior to the study date, and defining depression and anxiety as being present if there was a diagnosis within the previous 12 months. We will use existing morbidity code lists and algorithms for smoking status, BMI and ethnicity.[5, 6]

Outcomes

The main outcomes will be prevalence of alcohol recording using different code-based definitions.

The following outcomes will be assessed to determine the prevalence of recording of morbidity codes related to alcohol (**Objective 1)**:

a. Any morbidity coding relating to alcohol use, using Clinical, Referral and Additional files
b. Codes relating to physical/psychological harm due to alcohol use (e.g. 'alcoholic cirrhosis of liver')
c. Codes relating to alcohol use disorders (e.g. 'alcohol dependence syndrome')
d. Codes relating to management of alcohol use (e.g. 'referral to specialist alcohol treatment service')
e. Codes indicating level of alcohol use (including codes such as 'drinks occasionally', units per week, and

For peer review only - http://bmjopen.bmj.com/site/about/guidelines.xhtml

Applicants must complete all sections listed below
Sections which do not apply should be completed as *'Not Applicable'*

codes for screening)

We will use the following outcomes to determine the prevalence and frequency of alcohol screening using recommended tools, recording of the number of units of alcohol consumed per week, and other alcohol screening using morbidity codes, and to investigate how prevalence of these varies with patient characteristics (**Objective 2**):

f. Read codes indicating current drinking status, categorized as 'Current', 'Ex-drinker' or 'Non-drinker'
g. Read codes indicating level of alcohol use (e.g. 'social drinker', 'drinks occasionally')
h. Alcohol status, using data from the CPRD Additional Clinical Details file recorded under Entity Type 5, data field 1 (status) (Entity Type is the identifier that represents the structured data area in Vision where the data was entered), categorized as 'Current', 'Ex-drinker' or 'Non-drinker'.
i. Alcohol units consumed per week, using an existing (CALIBER: https://www.ucl.ac.uk/health-informatics/caliber) variable definition for 'number of alcohol unit consumption per week recorded in primary care (CPRD)',[7] using data from the CPRD Additional Clinical Details file recorded under Entity Type 5, data field 2 (units per week). Units per week will be categorized as "None", "1-14, "15-42" or "43+" units per week. 14 units per week is the recommended limit for men and women according to current UK government guidelines.[8]
j. Read codes indicating screening using a recommended tool (AUDIT/FAST).
k. Scores resulting from AUDIT/FAST screening (i.e. associated with a Read morbidity code indicating use of AUDIT/FAST), which will be obtained from the Additional Clinical Details file under Entity Type 172. Scores will be categorised according to WHO guidelines on risk level: 1-7 - low risk drinking; 8-15 - hazardous drinking; 16-19 - harmful drinking; 20-40 – possible dependence.[9]

We will also use both the recorded number of units of alcohol consumed per week and AUDIT/FAST scores to validate alcohol-related morbidity coding (**Objective 3**).

N. Data/Statistical Analysis

We will present the overall prevalence of alcohol recording using different electronic health record based definitions, listed in **M. Exposures, Health Outcomes and Covariates** as outcomes a–e. (**Objective 1**).

We will then present the prevalence of recording of at least one of each of the following within five years prior to the study date: (i) alcohol screening using Read morbidity codes relating to current drinking status or level of alcohol use (**M. Exposures, Health Outcomes and Covariates** outcomes f. and g.), (ii) alcohol status from the Additional Clinical Details file (outcome h.), (iii) alcohol units per week (outcome i.), and (iv) alcohol screening using recommended screening tools (AUDIT/FAST) (outcomes j. and k.), across a range of potential exposures (**Objective 2**). We will also present the frequency of consultations with recorded alcohol use codes in the 5 years before the study date, and the number of days from a patient's registration to their first alcohol use code.

For peer review only - http://bmjopen.bmj.com/site/about/guidelines.xhtml

Applicants must complete all sections listed below
Sections which do not apply should be completed as '*Not Applicable*'

We will present results for patients who were at least 18 years old on the study date separately from patients who were 16-17 years old.

In order to validate information gathered from alcohol morbidity coding (**Objective 3**), we will present median scores for recommended screening tools (AUDIT/FAST) recorded in the same patients on the same date as each patient's most recent record of: (i) alcohol screening using Read morbidity codes relating to current drinking status or level of alcohol use (outcomes f. and g.), (ii) alcohol status from the Additional Clinical Details file (outcome h.), (iii) alcohol units per week (outcome i.), or (iv) alcohol screening using recommended screening tools (AUDIT/FAST) (outcomes j. and k.) (see table shell for Objective 3 below). We will also present the median units per week on the same date as a record of alcohol status from the Additional Clinical Details file, or other alcohol screening using Read codes relating to current drinking status or level of alcohol use.
In sensitivity analyses, we will present median AUDIT/FAST scores and units per week recorded up to 30 days and up to 12 months before or after alcohol morbidity coding.

Table shell for Objective 3 (validation of alcohol morbidity coding):

	Codes of interest	Median (IQR) AUDIT/FAST score on the same date	Median (IQR) alcohol units per week on the same date
**Codes for alcohol status (current/non/ex)[a]**	Codes for non-drinker		
	Codes for current drinker		
	Codes for ex-drinker		
**Codes for level of alcohol use[b]**	Codes for non-drinker		
	Codes for light/occasional alcohol use		
	Codes for moderate alcohol use		
	Codes for heavy alcohol use		
**Alcohol status from Additional Clinical Details file[c]**	Non-drinker		
	Current drinker		
	Ex-drinker		
**Alcohol units per week[d]**	None		NA
	1-14		NA
	15-42		NA
	43+		NA
	No measure of units per week		NA

For peer review only - http://bmjopen.bmj.com/site/about/guidelines.xhtml

Applicants must complete all sections listed below
Sections which do not apply should be completed as '*Not Applicable'*

	available		

[a] Read codes indicating current drinking status (see M. Exposures, Health Outcomes and Covariates, outcome f.)

[b] Read codes indicating level of alcohol use (see M. Exposures, Health Outcomes and Covariates, outcome g.)

[c] Alcohol status, using data from the CPRD Additional Clinical Details file recorded under Entity Type 5 (see M. Exposures, Health Outcomes and Covariates, outcome h.)

[d] Alcohol units per week (see M. Exposures, Health Outcomes and Covariates, outcome i.)

Sensitivity analyses

1. In the main analysis, we will restrict the validation part of the study to AUDIT/FAST scores recorded on the same date as the each patient's most recent alcohol record of each type. In sensitivity analyses we will:
   a. Include any AUDIT/FAST score recorded up to 30 days before or after the code of interest.
   b. Include any AUDIT/FAST score recorded up to 12 months before or after the code of interest.
2. In the main analysis, we will record liver disease, hypertension and diabetes mellitus based on presence of a diagnosis at any time prior to the study date. We will test this definition by repeating the analysis limiting to diagnoses of these comorbidities within five years prior to the study date.
3. In the main analysis, we will record depression and anxiety based on presence of a diagnosis within five years prior to the study date. We will test this definition by repeating the analysis limiting to diagnoses of these comorbidities within 12 months prior to the study date.

O. Plan for addressing confounding

This is not relevant in this descriptive study. However, we will be describing the prevalence of comorbidities which may be confounders for future research.

P. Plans for addressing missing data

In this descriptive study we will simply describe the proportion of missing data for exposure variables and potential biases that may arise as a result. Since our primary outcome of interest is whether alcohol use is recorded, missing data on alcohol use is part of the measurement of the outcome.

Q. Patient or user group involvement (if applicable)

There will be no patient or user group involvement in this study.

R. Plans for disseminating and communicating study results, including the presence or absence of any restrictions on the extent and timing of publication

The study findings will be submitted for publication in peer-reviewed scientific journals, and will be presented at appropriate conferences and other meetings.

For peer review only

Applicants must complete all sections listed below
Sections which do not apply should be completed as '*Not Applicable'*

S. Limitations of the study design, data sources, and analytic methods

Not all GP practices contribute to CPRD, and patients may refuse to participate in the CPRD programme. This means that although we will use all available CPRD data this will not give us a complete picture of alcohol use recording and screening in UK primary care. There may be some selection bias since practices that contribute to CPRD may be more likely to adhere to guidelines including regular alcohol screening. In addition some of the definitions of alcohol use (e.g. recording of entity codes) we intend to use are specific to the Vision software used by practices contributing to CPRD, and therefore our findings using these data will not be transferable to data collected by practices using alternative electronic health record software.

Since our primary outcome of interest is completeness and accuracy of recording of alcohol use, missing data and misclassification of alcohol use using different code-based definitions are part of the measurement of the outcome. However, those without lifestyle covariates and ethnicity recorded may be less likely to have alcohol use recorded (as it is likely that this type of information would be recorded during the same type of patient contact). We will describe the proportion of missing data for these variables and potential biases that may arise as a result.

In the internal validation element of the study (**Objective 3**), we will use AUDIT/FAST scores as the 'gold standard' for validation of alcohol use morbidity codes and units per week. Although the AUDIT and FAST tests are considered valid in comparison with other self-reported alcohol measures,[10, 11] self-reported measures of alcohol use are limited as levels of alcohol use are likely to be under-reported. In future we may explore the association between alcohol use recorded in CPRD and health outcomes directly attributable to alcohol, such as alcohol-related liver disease, to externally validate alcohol use codes in the CPRD.

We will be using AUDIT/FAST scores recorded on the same date of the codes of interest. GP practices that record both measures during the same consultation may be different to practices that only record one measure; perhaps more likely to record alcohol use measures accurately. We will deal with this via sensitivity analyses using AUDIT/FAST scores recorded within 30 days or 12 months before or after the date of the alcohol-related code of interest.

We may be less likely to find sufficient numbers of AUDIT/FAST scores to validate morbidity codes for non-drinking and light drinking, since these patients may be less likely to be screened. However this may not be the case if patients are screened routinely on joining a new practice or as part of the NHS Health Check.

T. References

1. NICE, *Alcohol-use disorders: prevention. NICE guidelines PH24.* 2010.
2. Babor, T.F. and J.C. Higgins-Biddle, *BRIEF INTERVENTION For Hazardous and Harmful Drinking: A Manual for Use in Primary Care.* 2001.
3. Public Health England. *NHS Health Check: Best practice guidance*. 2017; Available from: http://www.healthcheck.nhs.uk/document.php?o=1308.

For peer review only - http://bmjopen.bmj.com/site/about/guidelines.xhtml

Applicants must complete all sections listed below
Sections which do not apply should be completed as '*Not Applicable*'

4. Otete, H.E., et al., *Sex and age differences in the early identification and treatment of alcohol use: a population-based study of patients with alcoholic cirrhosis.* Addiction, 2015. **110**(12): p. 1932-40.
5. Mathur, R., et al., *Completeness and usability of ethnicity data in UK-based primary care and hospital databases.* J Public Health, 2014. **36**(4): p. 684-92.
6. Bhaskaran, K., et al., *Representativeness and optimal use of body mass index (BMI) in the UK Clinical Practice Research Datalink (CPRD).* BMJ Open, 2013. **3**(9): p. e003389.
7. Denaxas, S.C., et al., *Data resource profile: cardiovascular disease research using linked bespoke studies and electronic health records (CALIBER).* Int J Epidemiol, 2012. **41**(6): p. 1625-38.
8. Department of Health, *UK Chief Medical Officers' alcohol guidelines review: Summary of the proposed new guidelines.* 2016.
9. Babor, T.F., et al. *The Alcohol Use Disorders Identification Test: Guidelines for Use in Primary Care*. 2001; Available from: http://www.talkingalcohol.com/files/pdfs/WHO_audit.pdf.
10. Allen, J.P., et al., *A review of research on the Alcohol Use Disorders Identification Test (AUDIT).* Alcohol Clin Exp Res, 1997. **21**(4): p. 613-9.
11. Reinert, D.F. and J.P. Allen, *The Alcohol Use Disorders Identification Test (AUDIT): A Review of Recent Research.* Alcoholism: Clinical and Experimental Research, 2002. **26**(2): p. 272-279.

List of Appendices *(Submit all appendices as separate documents to this application)*

Preliminary code lists for all variables are included in the Appendix (Excel workbook CPRD_ISAC_AUDIT_appendix_codelists.xlsx).

Protocol amendment – 31st January 2019

K. Study population
In the main study, there were too few patients aged 16-18 with alcohol recorded to report separate statistics for that group. Therefore, patients aged 16-18 were excluded from analysis.

M. Exposures, Health Outcomes§ and Covariates
Drinking status data taken from Read codes ((f) above), and drinking status data taken from the structured additional details file ((h) above) will now be combined to report overall drinking status, with the most recent of the two indicating current drinking status.

Derived current drinking status will also be calculated based on:

i. drinking status (current drinker, ex, non; either as recorded in Read codes, or in additional data),
ii. level of drinking (heavy, moderate, light drinker; as recorded in Read codes),
iii. units per week,
iv. AUDIT or FAST scores

Patients without data in (i) drinking status, will be categorised as current drinkers if they have codes for heavy, moderate or light drinkers, if they have units per week>0, or AUDIT/FAST>0.

N. Data/Statistical analysis

Time trends in alcohol recording
An additional analysis will investigate recording of alcohol use over time. Annually between 2009 and 2018, we will report

(i) The proportion of patients with a recorded alcohol status in the five years prior to 1st January, and
(ii) The proportion of patients with a record of the level of alcohol consumption in the five years prior to 1st January.

Each year, we will carry out a cross sectional study to estimate these proportions. Eligibility each year will be the

For peer review only - http://bmjopen.bmj.com/site/about/guidelines.xhtml

Applicants must complete all sections listed below
Sections which do not apply should be completed as *'Not Applicable'*

same as in the main study (adults aged 18+ on 1st January, with at least one year of follow-up prior to study date).

External validation compared to the Health Survey for England
For each patient, the most recent alcohol record to the study date, indicating status or units per week will taken. Patients with a recorded status of 'non' or 'ex-drinker' will be combined. For men and women separately, current status will be described and compared to the Health Survey for England 2016. Then, for patients with a recorded 'current' drinking status, the number of units per week will grouped into the following categories:
Among men:

- ≤14 (lower risk),
- 15-50 (increased risk),
- >50 (higher risk);

Among women:

- ≤14 (lower risk),
- 15-35 (increased risk),
- >35 (higher risk).

The proportions of patients in these categories, and the proportion of non- and current drinkers will be compared to the Health Survey for England.

For peer review only - http://bmjopen.bmj.com/site/about/guidelines.xhtml

The RECORD statement – checklist of items, extended from the STROBE statement, that should be reported in observational studies using routinely collected health data.

	Item No.	STROBE items	Location in manuscript where items are reported	RECORD items	Location in manuscript where items are reported
**Title and abstract**					
	1	(a) Indicate the study's design with a commonly used term in the title or the abstract (b) Provide in the abstract an informative and balanced summary of what was done and what was found	Page 1	RECORD 1.1: The type of data used should be specified in the title or abstract. When possible, the name of the databases used should be included.<br><br>RECORD 1.2: If applicable, the geographic region and timeframe within which the study took place should be reported in the title or abstract.<br><br>RECORD 1.3: If linkage between databases was conducted for the study, this should be clearly stated in the title or abstract.	Page 3<br><br>Page 3<br><br>n/a
**Introduction**					
Background rationale	2	Explain the scientific background and rationale for the investigation being reported	Page 4		
Objectives	3	State specific objectives, including any prespecified hypotheses	Page 5		
**Methods**					
Study Design	4	Present key elements of study design early in the paper	Page 6		
Setting	5	Describe the setting, locations, and relevant dates, including periods of recruitment, exposure, follow-up, and data collection	Page 6		

For peer review only

Participants	6	*(a) Cohort study* - Give the eligibility criteria, and the sources and methods of selection of participants. Describe methods of follow-up<br>*Case-control study* - Give the eligibility criteria, and the sources and methods of case ascertainment and control selection. Give the rationale for the choice of cases and controls<br>*Cross-sectional study* - Give the eligibility criteria, and the sources and methods of selection of participants<br><br>*(b) Cohort study* - For matched studies, give matching criteria and number of exposed and unexposed<br>*Case-control study* - For matched studies, give matching criteria and the number of controls per case	Page 6	RECORD 6.1: The methods of study population selection (such as codes or algorithms used to identify subjects) should be listed in detail. If this is not possible, an explanation should be provided.<br><br>RECORD 6.2: Any validation studies of the codes or algorithms used to select the population should be referenced. If validation was conducted for this study and not published elsewhere, detailed methods and results should be provided.<br><br>RECORD 6.3: If the study involved linkage of databases, consider use of a flow diagram or other graphical display to demonstrate the data linkage process, including the number of individuals with linked data at each stage.	Page 6
Variables	7	Clearly define all outcomes, exposures, predictors, potential confounders, and effect modifiers. Give diagnostic criteria, if applicable.	Page 7	RECORD 7.1: A complete list of codes and algorithms used to classify exposures, outcomes, confounders, and effect modifiers should be provided. If these cannot be reported, an explanation should be provided.	Appendices and online via link
Data sources/ measurement	8	For each variable of interest, give sources of data and details of methods of assessment (measurement).<br>Describe comparability of assessment methods if there is more than one group	Page 7, online via link on page 7		

For peer review only

Bias	9	Describe any efforts to address potential sources of bias	Discussion		
Study size	10	Explain how the study size was arrived at	Appendix Figure 1		
Quantitative variables	11	Explain how quantitative variables were handled in the analyses. If applicable, describe which groupings were chosen, and why	Page 6, page 11		
Statistical methods	12	(a) Describe all statistical methods, including those used to control for confounding<br>(b) Describe any methods used to examine subgroups and interactions<br>(c) Explain how missing data were addressed<br>(d) *Cohort study* - If applicable, explain how loss to follow-up was addressed<br>*Case-control study* - If applicable, explain how matching of cases and controls was addressed<br>*Cross-sectional study* - If applicable, describe analytical methods taking account of sampling strategy<br>(e) Describe any sensitivity analyses	Pages 8-9		
Data access and cleaning methods		..		RECORD 12.1: Authors should describe the extent to which the investigators had access to the database population used to create the study population.	Page 23

For peer review only

				RECORD 12.2: Authors should provide information on the data cleaning methods used in the study.	
Linkage		..		RECORD 12.3: State whether the study included person-level, institutional-level, or other data linkage across two or more databases. The methods of linkage and methods of linkage quality evaluation should be provided.	n/a
**Results**					
Participants	13	(a) Report the numbers of individuals at each stage of the study (*e.g.*, numbers potentially eligible, examined for eligibility, confirmed eligible, included in the study, completing follow-up, and analysed)<br>(b) Give reasons for non-participation at each stage.<br>(c) Consider use of a flow diagram		RECORD 13.1: Describe in detail the selection of the persons included in the study (*i.e.,* study population selection) including filtering based on data quality, data availability and linkage. The selection of included persons can be described in the text and/or by means of the study flow diagram.	Page 6
Descriptive data	14	(a) Give characteristics of study participants (*e.g.*, demographic, clinical, social) and information on exposures and potential confounders<br>(b) Indicate the number of participants with missing data for each variable of interest<br>(c) *Cohort study* - summarise follow-up time (*e.g.*, average and total amount)	Table 1<br>Table 1		
Outcome data	15	*Cohort study* - Report numbers of outcome events or summary measures over time<br>*Case-control study* - Report numbers in each exposure	Table 1		

For peer review only

		category, or summary measures of exposure<br>*Cross-sectional study* - Report numbers of outcome events or summary measures			
Main results	16	(a) Give unadjusted estimates and, if applicable, confounder-adjusted estimates and their precision (e.g., 95% confidence interval). Make clear which confounders were adjusted for and why they were included<br>(b) Report category boundaries when continuous variables were categorized<br>(c) If relevant, consider translating estimates of relative risk into absolute risk for a meaningful time period	n/a		
Other analyses	17	Report other analyses done—e.g., analyses of subgroups and interactions, and sensitivity analyses	Pages 13-15		
**Discussion**					
Key results	18	Summarise key results with reference to study objectives	Page 16		
Limitations	19	Discuss limitations of the study, taking into account sources of potential bias or imprecision. Discuss both direction and magnitude of any potential bias	Page 18	RECORD 19.1: Discuss the implications of using data that were not created or collected to answer the specific research question(s). Include discussion of misclassification bias, unmeasured confounding, missing data, and changing eligibility over time, as they pertain to the study being reported.	Page 18
Interpretation	20	Give a cautious overall interpretation of results considering objectives,	Pages 19		

		limitations, multiplicity of analyses, results from similar studies, and other relevant evidence			
Generalisability	21	Discuss the generalisability (external validity) of the study results	Page 19		
**Other Information**					
Funding	22	Give the source of funding and the role of the funders for the present study and, if applicable, for the original study on which the present article is based	Page 23		
Accessibility of protocol, raw data, and programming code		..		RECORD 22.1: Authors should provide information on how to access any supplemental information such as the study protocol, raw data, or programming code.	Page 23

*Reference: Benchimol EI, Smeeth L, Guttmann A, Harron K, Moher D, Petersen I, Sørensen HT, von Elm E, Langan SM, the RECORD Working Committee. The REporting of studies Conducted using Observational Routinely-collected health Data (RECORD) Statement. *PLoS Medicine* 2015; in press.

*Checklist is protected under Creative Commons Attribution (CC BY) license.

For peer review only

BMJ Open

Completeness and validity of alcohol recording in general practice within the UK: a cross-sectional study

Journal:	*BMJ Open*
Manuscript ID	bmjopen-2019-031537.R1
Article Type:	Original research
Date Submitted by the Author:	13-Sep-2019
Complete List of Authors:	Mansfield, Kathryn; London School of Hygiene and Tropical Medicine, Non-communicable Disease Epidemiology<br>Crellin, Elizabeth; Clinical Practice Research Datalink<br>Denholm, Rachel; University of Bristol, Centre for Academic Primary Care<br>Quint, Jennifer; Imperial College London, Respiratory Epidemiology, Occupational Medicine and Public Health<br>Smeeth, Liam; London School of Hygiene and Tropical Medicine, Epidemiology and Population Health<br>Cook, Sarah; London School of Hygiene & Tropical Medicine,<br>Herrett, Emily; London School of Hygiene and Tropical Medicine, Non communicable disease epidemiology
**Primary Subject Heading**:	Epidemiology
Secondary Subject Heading:	General practice / Family practice
Keywords:	alcohol, general practice, recording, validity, screening

SCHOLARONE™
Manuscripts

Completeness and validity of alcohol recording in general practice within the UK: a cross-sectional study

Kathryn Mansfield[1*], Elizabeth Crellin[2*], Rachel Denholm[3], Jennifer Quint[4], Liam Smeeth[1], Sarah Cook[1], Emily Herrett[1]

* Joint first authors.

1 - Department of Non-Communicable Diseases Epidemiology
London School of Hygiene and Tropical Medicine
London, UK

2 – Clinical Practice Research Datalink
3 – Centre for Academic Primary Care, Bristol Medical School,
University of Bristol,
Bristol, UK
4 – Imperial College London

Correspondence:
Dr Emily Herrett
Department of Non-Communicable Diseases Epidemiology
London School of Hygiene and Tropical Medicine
London UK
emily.herrett@lshtm.ac.uk

Word count 4644

For peer review only

Abstract

Background: Since 2010 the National Institute for Health and Care Excellence has recommended screening adults for excessive alcohol consumption to try and help prevent alcohol-use disorders. Little is known about the extent to which these recommendations are followed, and the resulting completeness and validity of alcohol-related data recording in primary care.

Objective: To investigate the completeness and accuracy of recording of alcohol use within primary care records in the UK.

Design and setting: Cross-sectional study in the Clinical Practice Research Datalink

Participants: We included all adult patients registered on 1st January 2018 with ≥1 year of follow-up.

Primary and secondary outcome measures: We calculated prevalence of alcohol consumption recording overall and within patient groups. We then validated alcohol consumption data against recommended screening tools (AUDIT) as the gold standard. We also calculated how prevalence of alcohol recording changed over the preceding decade.

Results: In 2018, among 1.8 million registered adult patients, just over half (51.9%) had a record for a code related to alcohol in the previous five years. Recording of alcohol consumption was more common among women, older people, ex-smokers, and those from more deprived areas, who were overweight/obese, or with comorbidities. A quarter of patients had units per week recorded in the last five years, but <10% had an AUDIT or FAST alcohol screening test score. The recorded alcohol measures corresponded to results from gold standard AUDIT scores. The distribution of consumption among current drinkers was similar to the Health Survey for England.

Conclusions: Half of adults in UK primary care have no recorded alcohol consumption data. When consumption is recorded, we have demonstrated internal and external validity of the data, suggesting greater recording may help identify opportunities for interventions to reduce harms.

For peer review only

For peer review only - http://bmjopen.bmj.com/site/about/guidelines.xhtml

Strengths and limitations of the study

- This study investigated alcohol recording across nearly 1.8 million UK adults in primary care, providing the power to understand in detail how alcohol use is recorded in general practice.
- We demonstrated the validity of alcohol recording internally within general practice records, and externally compared to the Health Survey for England.
- Though we demonstrated levels of alcohol recording and screening, it does not mean that GPs have acted upon the screening result, or that they have screened at an appropriate time.

For peer review only

For peer review only - http://bmjopen.bmj.com/site/about/guidelines.xhtml

Introduction

Hazardous alcohol use is a key behavioural risk factor affecting health. It is estimated that over 20% of adults in England are drinking at levels that are considered harmful ,[1] with a resulting cost to the NHS of over £3 billion per year.[2] In the age group 15-49 years, alcohol is the leading risk factor for ill health, early death and disability.[3]

Identification of high-risk patients can usefully be undertaken in primary care, where appropriate interventions can be promoted. The Alcohol Use Disorders Identification Test (AUDIT) [4] is the main tool for alcohol screening recommended by National Institute for Health and Care Excellence (NICE), though shortened versions (such as AUDIT-C and FAST) are also considered acceptable.[5] National Institute for Health and Care Excellence (NICE) guidelines published in 2010 recommend that opportunistic screening of the general population should take place during new patient registrations, and when screening or managing other conditions, promoting sexual health, during antenatal appointments and treating minor injuries. In addition, guidelines recommend screening 'high risk' groups, including those with relevant physical conditions (such as hypertension) and relevant mental health problems (such as depression).[5] Additionally, the NHS health check, which is offered to all people in the UK aged 40-74 years every five years, includes the AUDIT questions.[6]

Khadjesari et al (2013) showed relatively high levels of alcohol recording (76%) among new registrants in primary care between 2007 and 2009, although the use of validated screening tools was low (9%). They did not report levels for the whole registered patient population, which includes those who are less mobile and not subject to new patient registrations, nor specifically among patients with conditions that may be associated with, or exacerbated by, high-risk drinking. Poor recording of alcohol use has two major impacts: first and most importantly, patients may not be receiving appropriate interventions; and secondly, for

For peer review only

researchers, poor recording may prohibit reliable studies of the impact of alcohol on health using these data. Indeed, some recent studies using alcohol data in a large UK-based primary care electronic health record database (Clinical Practice Research Datalink [CPRD]) have indicated that bias may have arisen due to poor recording of alcohol data.[7, 8] For researchers using the data it is important to know not just whether screening occurs but if there are particular sub-groups more likely to be screened and to what extent alcohol codes used are meaningful reflections of actual alcohol use.

Using primary care data from the CPRD, this study assessed the utility of recorded alcohol data in primary care by assessing completeness of recording of alcohol status at different levels and by various patient level factors, and comparing measures of alcohol recorded in the CPRD internally and to external sources. Our study also offers insight into the completeness of alcohol data in primary care since the introduction of 2010 NICE guidance, including specifically among those identified as being at higher risk according to NICE recommendations, and provides guidance for future studies related to alcohol use using primary care data.

For peer review only - http://bmjopen.bmj.com/site/about/guidelines.xhtml

Methods

Study design and setting

We undertook a cross-sectional study with data from the Clinical Practice Research Datalink GOLD database, an ongoing primary care database containing the anonymised medical records from general practitioners in the UK using the Vision software to capture health record data with coverage of 15.4 million patients. Data from CPRD includes diagnoses, tests, clinical measurements, prescriptions and specialist referrals. CPRD uses Read morbidity coding to summarise each patient encounter with a code (or codes) that correspond to a standard set of clinical terminology. Roughly half of all practices in the CPRD have consented to data linkage and therefore deprivation data (based on quintiles of the Indices of Multiple Deprivation (IMD)) is available for these practices. The study included all patients in CPRD GOLD who were 18 years or over, alive and registered, for at least one year, with a CPRD GOLD practice on 1st January 2018.

Factors associated with alcohol recording

We investigated a range of potential factors that may be associated with alcohol-use recording in primary care: age, sex, deprivation, geographic region, selected comorbidities related to alcohol use that could be captured in electronic health record data (liver disease, hypertension, depression and anxiety) or those where health function monitoring is likely to be more frequent/complete (diabetes mellitus), time since registration at the current practice (1-5 years vs ≥5 years), body mass index (BMI), smoking status, and ethnicity.

We identified liver disease, hypertension and diabetes mellitus based on presence of a recorded diagnosis at any time prior to the study date. We identified depression and anxiety based on the presence of a diagnosis at any time within the five years prior to the study date (1st January 2018). We used existing morbidity code lists and algorithms to define smoking status, BMI and ethnicity.[9, 10]

For peer review only - http://bmjopen.bmj.com/site/about/guidelines.xhtml

Outcomes

Our main outcomes were the prevalence of alcohol-use recording in the five years prior to the study date, 1st January 2018. Prevalence of alcohol recording included any record of alcohol use or its effects, and we also broke it down into the following categories:

i. Any code suggesting that alcohol was discussed in the consultation.
ii. Codes indicating AUDIT (including short-form AUDIT-C and full AUDIT) or FAST screening.
iii. Codes quantifying alcohol use, comprised of:
    a. drinking status (current drinker, ex, non; either as recorded in Read codes, or in structured data),
    b. level of drinking (non, light, moderate, heavy drinker; as recorded in Read codes),
    c. units per week,
    d. AUDIT, AUDIT-C or FAST scores (AUDIT categorised according to WHO guidelines on risk level: 0-7 – non-drinker or low-risk drinking; 8-15 – hazardous drinking; 16-19 – harmful drinking; 20-40 – possible dependence.[11]).

We have made all morbidity code lists used in this study available to download (https://doi.org/10.17037/data.00001071). We have also provided further detail regarding variable definitions in the Appendix (**Supplementary Text 1 and Supplementary Table 1**).

We considered the record of a score from an alcohol-screening test using a validated tool (AUDIT, AUDIT-C or FAST) as the gold standard for alcohol recording. The AUDIT score is a 10-item instrument with a maximum score of 40 designed specifically for screening for hazardous or harmful alcohol use in primary care.[11, 12] A score of eight or more on the AUDIT tool is associated with harmful or hazardous drinking, although a slightly lower threshold can be used in women. The AUDIT tool can be abbreviated in time-limited

For peer review only - http://bmjopen.bmj.com/site/about/guidelines.xhtml

settings to the first three questions (dealing with alcohol consumption only) (AUDIT-C) to give a score out of 12 or a four-item screening tool (FAST), which give a score out 16. These abbreviated tools have lower thresholds to indicate harmful or hazardous drinking than the standard AUDIT tool: AUDIT-C – three for women and four for men; and FAST – three.[13-15] Due to differences in scoring systems and different threshold scores to identify problem dinking between the AUDIT, AUDIT-C and FAST tools, we restricted our internal validation to those with the full AUDIT or AUDIT-C score.

For peer review only

Statistical analysis

Completeness of alcohol recording

To investigate the completeness of alcohol recording, we estimated the proportion of patients with a record for one or more morbidity codes relating to alcohol in the categories outlined above (i.e. i. Any code suggesting that alcohol was discussed in the consultation; ii. Codes indicating AUDIT, AUDIT-C or FAST screening; or iii. Codes quantifying alcohol use) in the five years prior to 1st January 2018 (each patient could have codes recorded in more than one category). We then described alcohol recording prevalence in strata of individual characteristics (i.e. age, sex, and other factors described above under the heading 'Factors associated with alcohol recording'). We also described the frequency of consultations with a record of an alcohol code in the five years before the study date, and the number of days from a patient's registration to their first record of an alcohol use code.

Internal validity

We validated codes quantifying the level of alcohol use (i.e. current/ex/non; light/moderate/heavy; units/week [Category iii above]) against an AUDIT score from the *same date* and the *same patient*. Full AUDIT versus AUDIT-C screening were considered separately. We calculated median AUDIT and AUDIT-C scores for each category of level of alcohol use recording: (a) drinking status, (b) drinking level and (c) units per week.

For peer review only - http://bmjopen.bmj.com/site/about/guidelines.xhtml

Similarly, we also validated codes indicating drinking status, drinking level and AUDIT/AUDIT-C scores against the number of units per week. For each variable, we only included a patient's most recent record (to the study date).

External validity

We identified current alcohol-use status for each individual with a relevant primary care alcohol record by identifying the most recent record indicating alcohol intake; i.e. either a morbidity code indicating alcohol-use status (classified as current or non-/ex-drinkers) or a record indicating the number of units of alcohol consumed per week. We described current alcohol-use status separately for men and women from CPRD, and compared the results to the Health Survey for England 2016.[16] Then, for CPRD patients with a recorded 'current' drinking status, we grouped the recorded number of units consumed per week into the following categories: men ≤14 (lower risk), 15-50 (increased risk), >50 (higher risk); women ≤14 (lower risk), 15-35 (increased risk), >35 (higher risk), and again compared these data to the Health Survey for England data 2016.[16]

Time trends in alcohol recording

To understand how alcohol recording has been changing over time, we carried out a cross sectional study on the 1st January each year between 2009 and 2018. We estimated the number of patients who would be eligible for the study, using the same criteria as above (registered for one year at a CPRD practice, and aged 18 or over on the study date), and calculated the proportion of patients with a record of alcohol consumption (based on a record of drinking status), and level of drinking (based on Read codes, units per week, AUDIT and AUDIT-C scores) over the past five years.

For peer review only - http://bmjopen.bmj.com/site/about/guidelines.xhtml

Sensitivity analyses

1. In the main analysis, we restricted the validation of alcohol codes to cases where patients had an AUDIT/AUDIT-C score recorded on the same date as another alcohol code of interest. In sensitivity analyses, we included cases where any AUDIT/AUDIT-C score was recorded up to 30 days before or after the code of interest.
2. In the main analysis, we recorded liver disease, hypertension and diabetes mellitus based on presence of a diagnosis at any time prior to the study date. We tested this definition by repeating the analysis limiting to diagnoses of these comorbidities within five years prior to the study date.
3. In the main analysis, we recorded depression and anxiety based on presence of a diagnosis within five years prior to the study date. We tested this definition by repeating the analysis limiting to diagnoses of these comorbidities within 12 months prior to the study date.

Data were analysed using Stata v14 (StataCorp, Texas, USA).

Patient involvement

Neither patients nor the public were involved in the design or analysis of the study. This work uses data provided by patients and collected by the UK National Health Service as part of their care and support.

For peer review only - http://bmjopen.bmj.com/site/about/guidelines.xhtml

Results

There were 1,768,651 adults aged 18 years or over, alive and registered, for at least one year, with a CPRD practice on 1st January 2018, and therefore included in the study; 49.4% were male, and their mean age was 49.7 years. The mean duration of registration prior to the study date was 18.7 years (standard deviation 14.5 years). One fifth had hypertension, 2.0% had liver disease, 6.8% had diabetes, 6.7% had depression and 4.6% had anxiety.

Prevalence

Overall, 918,254 (51.9%) patients had a record indicating that alcohol was discussed within the last five years. This included diagnoses of alcohol use disorders, their management, harm due to alcohol consumption, in addition to the consumption of alcohol.

Drinking status

Drinking status (current, ex-, non-drinker) was recorded in 862,330 (48.8%) of patients, though this was not consistent across patient groups. Recording of drinking status was higher in women compared to men (52.7% in women vs 44.7% in men), increased with age (30.6% in 18-24 years, 62.2% in 75+ years) and was more common among recently registered patients (in the last one to five years) than in those registered for five or more years (73.6% vs 43.0%, respectively). There were also differences in recording between geographic region (higher recording in the North of England, lower recording in Northern Ireland and Wales), by deprivation (recording was more common in deprived areas) and by ethnicity (highest for white patients). Patients who had missing smoking or BMI data had particularly low levels of alcohol status recorded, but even in those with smoking status recorded, drinking status was only recorded in approximately 50%-60% of patients. If smoking and BMI were recorded there were differences in the recording of alcohol status at different levels of smoking/BMI: for example, ex and current smokers were more likely to have alcohol status recorded than non-smokers, as were patients who were overweight.

For peer review only - http://bmjopen.bmj.com/site/about/guidelines.xhtml

Patients were also more likely to have alcohol status recorded with each of the morbidities investigated (liver disease, hypertension, diabetes, depression and anxiety). Patients with diabetes were the most likely to have alcohol levels recorded (82.3% had an alcohol record) (**Table 1**).

Level of consumption

Overall, 862,642 (48.8%) of adult patients had some level of alcohol consumption (including non-drinkers) recorded in the previous five years, identified through recording of Read codes (heavy, moderate, light or non-drinker), units per week or formal alcohol screening score using AUDIT, AUDIT-C or FAST.

Level of alcohol consumption data were recorded using Read coding for 31.6% of patients, in units per week for 24.4% of patients, and via a record of formal alcohol screening for 11.5% of patients with a Read code indicating that they had completed an AUDIT, AUDIT-C or FAST questionnaire, but only 8.5% of patients had the actual score available.

Recording of level of alcohol consumption by patient and practice characteristics are shown in **Table 1**. Patients with diabetes had the highest levels of alcohol consumption recording, but only 39.5% of them had a recording of units per week, and only 10.1% had a recorded AUDIT, AUDIT-C or FAST score.

Among patients with a recorded drinking status, 20.3% were non-drinkers, 3.7% ex-drinkers, and 76.1% current drinkers. **Table 2** describes levels of drinking amongst patients for whom it was recorded.

If we use all data (from all levels of alcohol consumption categories, i.e. coded heavy/moderate/light/non-drinker, recorded units per week or formal alcohol screening

For peer review only - http://bmjopen.bmj.com/site/about/guidelines.xhtml

score) to derive drinking status based on recorded status, units per week, and AUDIT scores, an additional 35,105 patients (2.0% of eligible patients) have a drinking status. This derived status shows that 20.8% are non-drinkers, 3.5% ex-drinkers and 75.7% are current drinkers.

Internal validity

In our study population, there were 77,212 (4.4%) patients with at least one AUDIT score record available, 21,099 (1.2%) patients with at least one AUDIT-C score record available, and 431,394 (24.4%) with units-per-week records. **Table 2** describes the cross validation between alcohol measures and AUDIT scores, AUDIT-C scores and units per week.

Drinking status: Current drinking (based on Read coding) was associated with a median AUDIT-C score of three (IQR 2-5), which is under the standard threshold defining risky drinking, and non/ex drinkers had a median AUDIT-C score of zero. For those with full AUDIT scores, the non and ex drinkers had median scores of zero, and current drinkers had a score of three (IQR 1-5). On average, current drinkers drank six units per week (IQR 2-14).

Drinking level: light and moderate drinkers had low AUDIT and AUDIT-C scores, while heavy drinkers had a median AUDIT-C score of 8 (IQR 6-9) and a median AUDIT score of 6 (IQR 5-9), which indicates alcohol education. Light drinkers drank 3 units per week (IQR 1-8), moderate drinkers 6 per week (IQR 2-14), and heavy drinkers 18 per week (IQR 9-30).

Units per week: increased with increasing AUDIT and AUDIT-C scores, but even the highest category of units per week (43+) only had a median AUDIT score of 11 (IQR 8-12), which indicates simple alcohol advice.

AUDIT scores: the majority (92.5%) of patients had scores in the lowest category (AUDIT score 0-7, where alcohol education is recommended according to WHO guidelines [11]). Patients with higher AUDIT scores consumed more units per week; for example, those in the lowest AUDIT score category (0-7,alcohol education) had a median units per week of four (IQR 2-10) compared to a median of 55 units per week (IQR 32-70) in the highest AUDIT

For peer review only - http://bmjopen.bmj.com/site/about/guidelines.xhtml

score category (score 20-40, indicating need for referral to specialist for diagnostic evaluation and treatment according to WHO guidelines [11]).

AUDIT-C scores: Patients with low risk AUDIT-C scores (<5) consumed fewer units per week; median 4 units per week (IQR 2-8) compared to a median of 12 units per week (IQR 6-20) in the high risk category (score ≥5). Where there were records for both AUDIT and AUDIT-C, in those with high risk AUDIT-C scores (which would indicate the need for a full AUDIT) the median AUDIT score was 6 (IQR 5-7).

For peer review only

External validity

For men and women, the proportions of current and non-drinkers identified using Read-coding in CPRD were close to those estimated in HSE (men: 80% current drinkers in CPRD, 83% in HSE; women: 73% current drinkers in CPRD, 78% in HSE). Units per week were also broadly comparable between CPRD and HSE although prevalence of higher risk drinking in CPRD looked slightly lower (**Table 3**). However, for estimates for both the proportion of current drinkers and units consumed per week were slightly lower in CPRD than HSE.

Time trends

Figure 1 illustrates time trends in recording of alcohol status and level of consumption. Between 2009 and 2018, there was an increase in recording in CPRD of both alcohol status and level of alcohol consumption from 19% to over 50%.

Frequency of recording

Among patients with at least one alcohol record in the last five years, 48.9% had just one record, 21.8% had two records, 10.8% had three records, and the remaining 48.5% had four or more records. 51.4% of patients with a record had their first alcohol record on the date of registration, 84.4% within the first month, and 95.5% within the first year of registration.

For peer review only - http://bmjopen.bmj.com/site/about/guidelines.xhtml

For patients with more than one record of alcohol consumption, the median duration between records was 339 days (IQR 175-452). This was shorter for patients with heavy consumption (237 days (IQR 70-432) compared with light drinkers (median 351 (195-459 days)) or non-drinkers (median 335 (179-438 days)).

For peer review only

For peer review only - http://bmjopen.bmj.com/site/about/guidelines.xhtml

Sensitivity analyses

1. Internal validity where we required that AUDIT score and units per week were recorded within 30 days of another code (rather than on the same date) resulted in more records being validated, but the results were identical to the main analysis (**Appendix Table 2**).

2. Individuals with diabetes mellitus, hypertension and liver disease diagnosed in the five years prior to the study date had slightly higher alcohol recording than shown in the main analysis (where these variables included all patients ever diagnosed) (Diabetes 84.7% with recorded alcohol use in sensitivity analysis compared to 83.0% in main analysis with diabetes ever recorded; hypertension 75.8% with recorded alcohol use in sensitivity analysis, compared to 72.6% in main analysis; and liver disease 77.1% with recorded alcohol use in sensitivity analysis, compared to 69.9% in main analysis).

3. Similarly, depression and anxiety diagnosed in the past year had higher levels of alcohol recording than when diagnosed in the previous five years (depression: 62.4% vs 61.0% in main analysis; anxiety: 62.0% vs 60.9% in main analysis).

For peer review only

Discussion

Summary

In 2018, information about alcohol consumption was available for roughly half of all adult patients registered with practices contributing to the CPRD. Large differences in recording were seen across patient characteristics, in particular increasing with age, deprivation, and higher in patients with liver disease, hypertension, diabetes mellitus, depression and anxiety. Patients who were missing data on other risk factors such as smoking, BMI and ethnicity were also less likely to have any recording of alcohol use. Those with missing alcohol, smoking, BMI and ethnicity data may represent a group who have minimal contact with primary care. However, even among those with smoking recorded, GPs only recorded drinking status in approximately 50%-60% of patients. This suggests that GPs record alcohol use less frequently than smoking status; so even when there are opportunities to ask about health behaviours, alcohol use in not necessarily included. Alcohol was more likely to be recorded in those with higher levels of other health risks (smokers, obese) also suggesting that GPs may be more likely to ask about alcohol in patients who they perceive as at higher risk. These patients may also visit the GP more frequently, increasing the opportunities to ask about alcohol use.

The patterns of alcohol recording seen here show that UK GPs may be failing to record alcohol consumption at the most basic level of drinking status for half of their adult population. However, patients who are at greater risk from alcohol-related illness are being appropriately targeted (i.e. those with mental health conditions and diabetes, liver disease, hypertension) and have better recording. Importantly, 20-40% of these at-risk patients still lack data on alcohol consumption and the proportion of patients screened for alcohol consumption appropriately using a valid screening tool is even lower (although one explanation for this observations might be that individuals scoring at low risk are screened but this information is not recorded). Alcohol use should be discussed as part of the

For peer review only - http://bmjopen.bmj.com/site/about/guidelines.xhtml

cardiovascular disease health check for all patients over 40,[17] and suboptimal levels of alcohol recording are consistent with the low uptake of the health check recently described.[18] Importantly, just one third of 18-24 year olds had alcohol consumption recorded, and they are particularly likely to enagage in risky drinking behaviours.

Compared to a 2013 study using CPRD data (Khadjesari et al, 2013[19]), our study showed that while recording among new registrants has plateaued since 2009, remaining at around 75%, screening with AUDIT appears to have increased from 9% among new registrants in 2009 to 21.8% in our study. This may correspond with the introduction of new NICE guidance in 2010.[5] However, screening with a recommended screening tool for all registered adults remains at only 8%.

Our internal validation showed that, when there was a record of level of alcohol consumption, other measures of intake recorded in CPRD were likely to correspond. Recording of units per week corresponded to AUDIT scores (full AUDIT and AUDIT-C), and both measures corresponded to informal coding of alcohol consumption using Read codes (e.g. codes for light/moderate/heavy drinker) although the informal codes were less useful for discrimination between heavier drinkers (the median AUDIT score in those with a heavy drinker code was 6). AUDIT scores were used as the 'gold standard' because they are considered valid in comparison with other self-reported alcohol measures,[20, 21] but all self-reported measures of alcohol use are limited as they are likely to be under-reported.[22]

The Health Survey for England found that, in 2016, 17% of men and 22% of women were teetotal. Using CPRD drinking status, 20.5% of patients were recorded as non-drinkers, so this is comparable and demonstrates external validity, albeit also self-reported. The internal and external validity demonstrated in this study give weight to any study using alcohol data from CPRD. While care must be taken to avoid introducing bias by restricting to only patients with alcohol recorded, the measures of consumption are likely to be useful in

For peer review only - http://bmjopen.bmj.com/site/about/guidelines.xhtml

ranking participants in terms of their alcohol of consumption, with the caveats required by all self-reported alcohol research.

Strengths and Limitations

This study investigated alcohol recording across nearly 1.8 million UK adults, providing the power to explore recording in different subgroups of patients, and to understand in detail how alcohol use is recorded in primary care.

To validate, we used AUDIT scores recorded on the same date as the codes of interest. If an AUDIT questionnaire is completed, subsequent recording of alcohol consumption (in Read codes or units per week) might be more accurate than at a time when no scoring is done, and validity may be lower outside of these times. Additionally, GP practices that record both measures during the same consultation may be different to practices that only record one measure, and perhaps more likely to record alcohol use measures accurately. However, our sensitivity analyses using AUDIT scores recorded within 30 days before or after the date of the alcohol-related code of interest showed that records within a month of an AUDIT score or measure of units were internally consistent. For the external validation we used data from the health survey for England 2016, whereas the time frame for the prevalence estimates of alcohol use from CPRD were from 2013-2018 however although the time frames do not match perfectly 2016 is within the middle of the time frame and this seems an appropriate comparator.

Evidence suggests alcohol use in young people is common[23]. Indeed, the importance of screening and prevention in young people has been highlighted by recent NICE guidelines promoting alcohol intervention and education in the under 25s.[24] Unfortunately, we were unable to include 16-17 year olds in our analyses due to limited alcohol records in this age group and the resulting risk to anonymity. However, the finding of limited alcohol recording in

For peer review only - http://bmjopen.bmj.com/site/about/guidelines.xhtml

16-17 year olds is, in itself, an interesting insight into primary care alcohol recording in this vulnerable population.

Although we have demonstrated that alcohol recording and screening are higher for patients with certain high-risk conditions, it does not mean that GPs have acted upon the screening result, or that they have screened at an appropriate time. For example, patients with depression or anxiety may not have been screened at the time when depression was diagnosed. We showed that the majority of screening took place within one month of registration, so whether this is repeated during consultations for high-risk conditions is not clear from these data.

Between 2009 and 2018, the proportion of CPRD patients with alcohol recorded more than tripled from around 13% to nearly 49%. It is likely that this increase in primary-care alcohol recording was contributed to by the 2010 NICE guidelines recommending screening on registration with a GP, and studies demonstrating the efficacy and cost effectiveness of alcohol screening and brief interventions for alcohol misuse.[25] Screening is most likely to happen at the time of registration, shown here and in Khadjesari et al[19], and therefore the increase in recording in our study may be linked to patient turnover. The result is that patients who have been at a practice for a long time are much less likely to have any record of their alcohol consumption; while recording has improved, there are clear and important differences between patients with and without a record for alcohol use.

Implications for research

This study helps establish best practise in using primary care data to investigate alcohol use in research. Researchers using CPRD data should be aware of the limitations of alcohol data. Though the validity of the existing data has been described here, using only patients with complete alcohol use data may introduce selection bias into studies by preferentially including older, sicker, and more deprived patients.

For peer review only - http://bmjopen.bmj.com/site/about/guidelines.xhtml

Implications for practice

This study provides insight into how well aligned routine primary care practice is with NICE guidelines for alcohol screening. Recording of alcohol in primary care is not complete, meaning that opportunities for intervention may not be being identified. While we found evidence that alcohol use recording is higher in the high-risk groups highlighted by NICE (those with hypertension, depression), and at key times of patient registration recommended by NICE (among newly registered patients), there are still large numbers of patients in whom we might expect risky drinking who are not having their alcohol use recorded, and who therefore may not be targeted for intervention. In particular, younger individuals, in whom alcohol is a major cause of morbidity, maybe being overlooked.

For peer review only

For peer review only - http://bmjopen.bmj.com/site/about/guidelines.xhtml

References

1. NHS Digital, *Health Survey for England, 2017: Multiple risk factors, Table 13.* 2018.
2. Home Office, *A minimum unit price for alcohol. Impact Assessment.* 2012.
3. GBD 2016 Alcohol Collaborators, *Alcohol use and burden for 195 countries and territories, 1990-2016: a systematic analysis for the Global Burden of Disease Study 2016.* Lancet, 2018. **392**(10152): p. 1015-1035.
4. Babor, T.F. and J.C. Higgins-Biddle, *BRIEF INTERVENTION For Hazardous and Harmful Drinking: A Manual for Use in Primary Care.* 2001.
5. NICE, *Alcohol-use disorders: prevention. NICE guidelines PH24.* 2010.
6. Public Health England. *NHS Health Check: Best practice guidance*. 2017; Available from: http://www.healthcheck.nhs.uk/document.php?o=1308.
7. Stewart, D., et al., *Alcohol consumption and all-cause mortality: an analysis of general practice database records for patients with long-term conditions.* J Epidemiol Community Health, 2017. **71**(8): p. 729-735.
8. Otete, H.E., et al., *Sex and age differences in the early identification and treatment of alcohol use: a population-based study of patients with alcoholic cirrhosis.* Addiction, 2015. **110**(12): p. 1932-40.
9. Mathur, R., et al., *Completeness and usability of ethnicity data in UK-based primary care and hospital databases.* J Public Health, 2014. **36**(4): p. 684-92.
10. Bhaskaran, K., et al., *Representativeness and optimal use of body mass index (BMI) in the UK Clinical Practice Research Datalink (CPRD).* BMJ Open, 2013. **3**(9): p. e003389.
11. Babor, T., et al., *The Alcohol Use Disorders Identification Test: Guidelines for Use in Primary Care. Available from http://apps.who.int/iris/bitstream/handle/10665/67205/WHO_MSD_MSB_01.6a.pdf?sequence=1*. 2001.
12. Saunders, J.B., et al., *Development of the Alcohol Use Disorders Identification Test (AUDIT): WHO Collaborative Project on Early Detection of Persons with Harmful Alcohol Consumption--II.* Addiction, 1993. **88**(6): p. 791-804.
13. Reinert, D.F. and J.P. Allen, *The alcohol use disorders identification test: an update of research findings.* Alcohol Clin Exp Res, 2007. **31**(2): p. 185-99.
14. Hodgson, R., et al., *The FAST Alcohol Screening Test.* Alcohol Alcohol, 2002. **37**(1): p. 61-6.
15. Hodgson, R.J., et al., *Fast screening for alcohol misuse.* Addict Behav, 2003. **28**(8): p. 1453-63.
16. NHS Digital, *Health Survey for England, 2016.* 2016.
17. Public Health England, *NHS Health Check: Best practice guidance. 2017; Available from: http://www.healthcheck.nhs.uk/document.php?o=1308*. 2017.
18. Coghill, N., et al., *NHS health checks: a cross- sectional observational study on equity of uptake and outcomes.* BMC Health Serv Res, 2018. **18**(1): p. 238.
19. Khadjesari, Z., et al., *Alcohol consumption screening of newly-registered patients in primary care: a cross-sectional analysis.* Br J Gen Pract, 2013. **63**(615): p. e706-12.
20. Allen, J.P., et al., *A review of research on the Alcohol Use Disorders Identification Test (AUDIT).* Alcohol Clin Exp Res, 1997. **21**(4): p. 613-9.
21. Reinert, D.F. and J.P. Allen, *The Alcohol Use Disorders Identification Test (AUDIT): A Review of Recent Research.* Alcoholism: Clinical and Experimental Research, 2002. **26**(2): p. 272-279.
22. Feunekes, G.I., et al., *Alcohol intake assessment: the sober facts.* Am J Epidemiol, 1999. **150**(1): p. 105-12.
23. NHS Digital, *Smoking, Drinking and Drug Use Among Young People in England - 2016*. 2017.
24. NICE, *Alcohol interventions in secondary and further education. NG135. Published 12 August 2019.* 2019.

For peer review only - http://bmjopen.bmj.com/site/about/guidelines.xhtml

25. Angus, C., et al., *What are the Implications for Policy Makers? A Systematic Review of the Cost-Effectiveness of Screening and Brief Interventions for Alcohol Misuse in Primary Care.* Front Psychiatry, 2014. **5**: p. 114.

For peer review only

For peer review only - http://bmjopen.bmj.com/site/about/guidelines.xhtml

Author statement

EC and KM contributed equally. EH, SC, KM, LS, JQ, EC and RD contributed to the design of the study. EH and EC extracted the data, EC, KM and EH wrote the statistical programmes, and wrote the first draft. All authors (EH, SC, KM, EC, LS, JQ, RD) contributed to further drafts and approved the final manuscript.

Conflicts of interest

None.

Funding

This research received no specific grant from any funding agency in the public, commercial or not-for-profit sectors. EH holds a NIHR postdoctoral fellowship (grant number PDF-2016-09-029).

Role of the funding source

The NIHR had no role in the design, analysis or writing up of this study.

Patient and public involvement

We did not involve patients or the public in our work.

Data access

EC and EH had full access to the CPRD database in order to create the study population.

Data sharing statement

We obtained data from the Clinical Practice Research Datalink (CPRD). CPRD is a research service that provides primary care and linked data for public health research. CPRD data governance, and our own license to use CPRD data, do not allow us to distribute or make available patient data directly to other parties. Researchers may apply for data access at www.cprd.com, and must have their study protocol approved by the Independent Scientific Advisory Committee for MHRA database research (details at www.cprd.com/isac).

For peer review only - http://bmjopen.bmj.com/site/about/guidelines.xhtml

For peer review only

Ethical approval

This study was approved by the London School of Hygiene and Tropical Medicine Ethics Committee (Approval Number 14454).

For peer review only

For peer review only - http://bmjopen.bmj.com/site/about/guidelines.xhtml

Tables

For peer review only

For peer review only - http://bmjopen.bmj.com/site/about/guidelines.xhtml

Table 1 Characteristics of adult patients registered in the CPRD, on 1st January 2018, and those with records indicating drinking status, level of consumption, units per week and AUDIT/FAST scores.

	Study population: All individuals 18+yrs registered with a CPRD practice on 1st January 2018*		Individuals in the study population with recording of alcohol use in different categories in the 5 years before 1st January 2018**															
			Has a record for any Read code relating to alcohol use		Has a record for a Read code indicating alcohol use, from which alcohol status could be derived[a]		Has a record of any drinking status recorded as Read code, or captured through structured data areas in GP software[b]		Has level of consumption recorded in Read codes[c]		Has units per week recorded		Has had AUDIT[d] or FAST screen		Has AUDIT[d] or FAST score recorded		Has any level of drinking recorded in Read, units or score[e]	
	N (col %)*		n (row %)**		n (row %)**		n (row %)**		n (row %)**		n (row %)**		n (row %)**		n (row %)**		n (row %)**	
**N**	1,768,651	(100)	918,254	(51.9)	897,535	(50.7)	862,330	(48.8)	558,747	(31.6)	431,394	(24.4)	204,182	(11.5)	150,457	(8.5)	862,642	(48.8)
**Sex**																		
Male	872,865	(49.4)	416,611	(47.7)	406,508	(46.6)	389,946	(44.7)	231,204	(26.5)	222,678	(25.5)	93,900	(10.8)	69,661	(8)	389,771	(44.7)
Female	895,786	(50.6)	501,643	(56)	491,027	(54.8)	472,384	(52.7)	327,543	(36.6)	208,716	(23.3)	110,282	(12.3)	80,796	(9)	472,871	(52.8)
**Age group**																		
18-24	168,796	(9.5)	58,379	(34.6)	54,830	(32.5)	51,584	(30.6)	36,755	(21.8)	16,537	(9.8)	15,629	(9.3)	8,564	(5.1)	51,647	(30.6)
25-34	285,015	(16.1)	131,905	(46.3)	128,051	(44.9)	119,772	(42)	76,097	(26.7)	50,686	(17.8)	34,313	(12)	25,222	(8.8)	120,830	(42.4)
35-44	300,819	(17)	136,265	(45.3)	133,289	(44.3)	125,865	(41.8)	77,277	(25.7)	60,492	(20.1)	34,273	(11.4)	26,405	(8.8)	126,761	(42.1)
45-54	333,132	(18.8)	171,021	(51.3)	167,309	(50.2)	160,742	(48.3)	96,024	(28.8)	88,593	(26.6)	37,693	(11.3)	28,709	(8.6)	160,636	(48.2)
55-64	277,217	(15.7)	160,137	(57.8)	157,180	(56.7)	152,786	(55.1)	94,656	(34.1)	86,476	(31.2)	32,478	(11.7)	24,618	(8.9)	151,987	(54.8)
65-74	223,120	(12.6)	145,105	(65)	142,776	(64)	139,320	(62.4)	92,173	(41.3)	77,896	(34.9)	29,967	(13.4)	22,483	(10.1)	139,043	(62.3)
75+	180,552	(10.2)	115,442	(63.9)	114,100	(63.2)	112,261	(62.2)	85,765	(47.5)	50,714	(28.1)	19,829	(11)	14,456	(8)	111,738	(61.9)
**Time since registration**																		
1-5 years	334,629	(18.9)	274,706	(82.1)	266,784	(79.7)	246,126	(73.6)	150,578	(45)	114,320	(34.2)	102,332	(30.6)	76,254	(22.8)	253,079	(75.6)
>=5 years	1,434,022	(81.1)	643,548	(44.9)	630,751	(44)	616,204	(43)	408,169	(28.5)	317,074	(22.1)	101,850	(7.1)	74,203	(5.2)	609,563	(42.5)
**Region**																		
North England	231,297	(13.1)	138,506	(59.9)	136,272	(58.9)	128,522	(55.6)	76,323	(33)	76,396	(33)	45,957	(19.9)	40,689	(17.6)	132,999	(57.5)
South England	607,016	(34.3)	320,297	(52.8)	308,052	(50.7)	285,002	(47)	174,695	(28.8)	143,027	(23.6)	106,862	(17.6)	77,866	(12.8)	295,640	(48.7)
Northern Ireland	114,311	(6.5)	55,838	(48.8)	53,203	(46.5)	52,981	(46.3)	34,830	(30.5)	22,297	(19.5)	13,363	(11.7)	1,104	(1)	50,621	(44.3)
Scotland	397,133	(22.5)	207,247	(52.2)	205,159	(51.7)	201,171	(50.7)	138,776	(34.9)	103,816	(26.1)	35,951	(9.1)	30,202	(7.6)	197,790	(49.8)
Wales	418,894	(23.7)	196,366	(46.9)	194,849	(46.5)	194,654	(46.5)	134,123	(32)	85,858	(20.5)	2,049	(0.5)	596	(0.1)	185,592	(44.3)
**Deprivation**																		
1 (least deprived)	190,262	(10.8)	102,692	(54)	99,633	(52.4)	90,969	(47.8)	44,305	(23.3)	57,838	(30.4)	34,487	(18.1)	27,071	(14.2)	95,812	(50.4)
2	134,191	(7.6)	74,536	(55.5)	72,437	(54)	66,664	(49.7)	35,727	(26.6)	39,943	(29.8)	25,722	(19.2)	20,614	(15.4)	69,844	(52)
3	118,777	(6.7)	67,455	(56.8)	65,713	(55.3)	61,953	(52.2)	36,121	(30.4)	33,892	(28.5)	20,030	(16.9)	15,365	(12.9)	63,199	(53.2)
4	108,991	(6.2)	62,745	(57.6)	61,689	(56.6)	58,234	(53.4)	39,674	(36.4)	26,879	(24.7)	19,040	(17.5)	15,816	(14.5)	59,403	(54.5)
5 (most deprived)	96,301	(5.4)	57,840	(60.1)	56,804	(59)	51,770	(53.8)	38,342	(39.8)	21,983	(22.8)	21,497	(22.3)	18,212	(18.9)	54,968	(57.1)
Missing	1,120,129	(63.3)	552,986	(49.4)	541,259	(48.3)	532,740	(47.6)	364,578	(32.5)	250,859	(22.4)	83,406	(7.4)	53,379	(4.8)	519,416	(46.4)

CONTINUED…

For peer review only - http://bmjopen.bmj.com/site/about/guidelines.xhtml

	Study population: All individuals 18+yrs registered with a CPRD practice on 1st January 2018* N (col %)*	Individuals in the study population with recording of alcohol use in different categories in the 5 years before 1st January 2018** Has a record for any Read code relating to alcohol use n (row %)**	Has a record for a Read code indicating alcohol use, from which alcohol status could be derived[a] n (row %)**	Has a record of any drinking status recorded as Read code, or captured through structured data areas in GP software[b] n (row %)**	Has level of consumption recorded in Read codes[c] n (row %)**	Has units per week recorded n (row %)**	Has had AUDIT[d] or FAST screen n (row %)**	Has AUDIT[d] or FAST score recorded n (row %)**	Has any level of drinking recorded in Read, units or score[e] n (row %)**
**Ethnicity**									
White	807,709 (45.7)	502,125 (62.2)	489,808 (60.6)	465,333 (57.6)	287,175 (35.6)	254,409 (31.5)	135,229 (16.7)	104,643 (13)	470,554 (58.3)
South Asian	46,593 (2.6)	28,089 (60.3)	27,111 (58.2)	25,603 (55)	21,297 (45.7)	5,874 (12.6)	9,611 (20.6)	6,835 (14.7)	26,306 (56.5)
Other	60,520 (3.4)	38,086 (62.9)	36,482 (60.3)	33,898 (56)	26,559 (43.9)	9,974 (16.5)	13,896 (23)	10,120 (16.7)	35,212 (58.2)
Missing	853,829 (48.3)	349,954 (41)	344,134 (40.3)	337,496 (39.5)	223,716 (26.2)	161,137 (18.9)	45,446 (5.3)	28,859 (3.4)	330,570 (38.7)
**Smoking status**									
Non smoker	746,074 (42.2)	383,302 (51.4)	373,622 (50.1)	356,424 (47.8)	233,122 (31.2)	163,733 (21.9)	91,386 (12.2)	66,152 (8.9)	359,039 (48.1)
Current smoker	254,850 (14.4)	147,616 (57.9)	144,275 (56.6)	139,028 (54.6)	90,505 (35.5)	68,758 (27)	31,617 (12.4)	23,328 (9.2)	137,108 (53.8)
Ex-smoker	565,583 (32)	347,945 (61.5)	342,126 (60.5)	332,036 (58.7)	213,908 (37.8)	181,109 (32)	71,773 (12.7)	53,832 (9.5)	331,127 (58.5)
Missing	202,144 (11.4)	39,391 (19.5)	37,512 (18.6)	34,842 (17.2)	21,212 (10.5)	17,794 (8.8)	9,406 (4.7)	7,145 (3.5)	35,368 (17.5)
**BMI**									
Normal (18·5–25 kg/m²)	533,130 (30.1)	298,415 (56)	290,354 (54.5)	277,070 (52)	172,796 (32.4)	139,540 (26.2)	74,169 (13.9)	54,609 (10.2)	278,618 (52.3)
Underweight (BMI <18·5 kg/m²)	32,421 (1.8)	16,915 (52.2)	16,262 (50.2)	15,481 (47.7)	10,904 (33.6)	6,055 (18.7)	4,327 (13.3)	2,938 (9.1)	15,570 (48)
Overweight/obese (BMI ≥25 kg/m²)	865,940 (49)	543,118 (62.7)	534,282 (61.7)	519,336 (60)	346,490 (40)	265,441 (30.7)	110,880 (12.8)	82,856 (9.6)	516,657 (59.7)
Missing	337,160 (19.1)	59,806 (17.7)	56,637 (16.8)	50,443 (15)	28,557 (8.5)	20,358 (6)	14,806 (4.4)	10,054 (3)	51,797 (15.4)
**Liver disease**	35,542 (2)	25,404 (71.5)	24,854 (69.9)	24,340 (68.5)	16,978 (47.8)	12,847 (36.1)	5,012 (14.1)	3,684 (10.4)	24,069 (67.7)
**Hypertension**	358,285 (20.3)	262,785 (73.3)	260,050 (72.6)	256,762 (71.7)	183,616 (51.2)	134,479 (37.5)	44,646 (12.5)	33,173 (9.3)	254,136 (70.9)
**Diabetes**	120,744 (6.8)	100,787 (83.5)	100,158 (83)	99,378 (82.3)	79,211 (65.6)	47,668 (39.5)	16,145 (13.4)	12,164 (10.1)	98,451 (81.5)
**Depression**	118,644 (6.7)	73,868 (62.3)	72,314 (61)	69,961 (59)	47,499 (40)	33,663 (28.4)	15,581 (13.1)	11,185 (9.4)	69,083 (58.2)
**Anxiety**	80,658 (4.6)	50,048 (62)	49,111 (60.9)	47,593 (59)	31,840 (39.5)	23,330 (28.9)	10,158 (12.6)	7,348 (9.1)	47,006 (58.3)

a. Alcohol status: Current drinker, ex-drinker, non-drinker from Read codes.
b. Alcohol status: Current drinker, ex-drinker, non-drinker from Read codes and captured through structured data areas in GP software.
c. Level of drinking: none, light, moderate, heavy drinker
d. Includes full AUDIT and short-form AUDIT-C screening.
e. Level of drinking derived from: Read codes, units per week or AUDIT/AUDIT-C/FAST screening score

*For the column (shaded) summarising characteristics for the whole study population (i.e. all adults registered in CPRD on 1st January 2018) – percentages are out of the whole study population (i.e. column %s).
**For subsequent columns (unshaded) – which cover individuals identified through different types of alcohol-use recording – percentages represent the number of individuals recorded with the specific category of coding as a percentage of the study population in that particular stratum; That is, row percentage with denominator from study population in that stratum (e.g. for all women in the study population [n=895,786], 56% [n=501,643] have a record of any Read code relating to alcohol use).

Table 2 Internal validation comparing measures of alcohol use in CPRD to AUDIT and AUDIT-C scores, and to units per week. N=1,768,651

For peer review only - http://bmjopen.bmj.com/site/about/guidelines.xhtml

	N	(%)	N patients with AUDIT score on the same date as record n	(%)	AUDIT score median	(IQR)	N patients with AUDIT C score on the same date as record n	(%)	AUDIT-C score median	(IQR)	N patients with units per week recorded on same date as record n	(%)	Units per week median	(IQR)
**Current drinking status***														
Non	174,970	(20.3)	2571	(1.5)	0	(0-0)	1022	(0.6)	0	(0-0)	905	(0.5)	0	(0-1)
Ex	31,526	(3.7)	292	(0.9)	0	(0-0)	39	(0.1)	0	(0-0)	1,004	(3.2)	0	(0-1)
Current	655,834	(76.1)	21563	(3.3)	3	(1-5)	4283	(0.7)	3	(2-5)	333,374	(50.8)	6	(2-14)
**Current drinking level (Read codes)**														
Non-drinker	222835	(39.9)	2880	(1.3)	0	(0-0)	1061	(0.5)	0	(0-0)	2,278	(1)	0	(0-2)
Light drinker	255,919	(45.8)	2,962	(1.2)	2	(1-3)	1,058	(0.4)	2	(1-3)	19,063	(7.4)	3	(1-8)
Moderate drinker	52,070	(9.3)	1,122	(2.2)	0	(0-4)	225	(0.4)	3	(2-4)	12,753	(24.5)	6	(2-14)
Heavy drinker	27,923	(5)	2,860	(10.2)	6	(5-9)	125	(0.4)	8	(6-9)	10,319	(36.9)	18	(9-30)
**Units per week**														
0	38,929	(9)	1,830	(4.7)	0	(0-0)	123	(0.3)	0	(0-1)				
1-14	298,441	(69.2)	10,905	(3.7)	3	(2-4)	1,982	(0.7)	3	(2-4)	n/a		n/a	
15-42	80,449	(18.6)	2,761	(3.4)	6	(4-8)	364	(0.5)	6	(4-7)				
43+	13,575	(3.1)	222	(1.6)	11	(8-12)	17	(0.1)	8	(6-10)				
**AUDIT C score category**														
0-4; Low risk	16,206	(76.8)	3,994	(24.6)	2	(0-3)	n/a		n/a		1,781	(11)	4	(2-8)
5-12; High risk	4,893	(23.2)	1,129	(23.1)	6	(5-7)					702	(14.3)	12	(6-20)
**AUDIT score category (and corresponding WHO recommended intervention[11])**														
0-7; Alcohol Education	71,401	(92.5)					4,881	(6.8)	2	(1-4)	14,156	(19.8)	4	(2-10)
8-15; Simple Advice	5,405	(7)					242	(4.5)	9	(8-10)	1,322	(24.9)	20	(14-30)
16-19; Simple Advice plus Brief Counseling and Continued Monitoring	206	(0.3)	n/a		n/a		0	(0)		(-)	45	(21.8)	35	(20-50)
20-40; Referral to Specialist for Diagnostic Evaluation and Treatment	200	(0.3)					0	(0)		(-)	22	(11.2)	55	(32-70)

*Current drinking status based on recorded status in Read codes and captured through structured data areas in GP software (not including derived status based on units consumed or AUDIT score).

For peer review only

Table 3 External validation, comparing most recent record of alcohol status in CPRD to data from the Health Survey for England 2016.

	CPRD		HSE
	n	%	%
**Men**			
**Non drinker***	76,544	20	17
**Current drinker**	313,402	80	83
**Units per week among current drinkers with units per week recorded:**			
≤14 (lower risk)	*122,787*	*67*	*63*
15-50 (increased risk)	*53,923*	*30*	*31*
>50 (higher risk)	*5,987*	*3*	*6*
**Women**			
**Non drinker***	129,952	28	22
**Current drinker**	342,432	73	78
**Units per week among current drinkers with units per week recorded:**			
≤14 (lower risk)	*146,177*	*88*	*78*
15-35 (increased risk)	*16,434*	*10*	*16*
>35 (higher risk)	*3,094*	*2*	*5*

*Includes patients labelled non- and ex-drinker

For peer review only

Figure captions

Figure 1. Recording of alcohol status and time trends between 2009 and 2018.

For peer review only

For peer review only - http://bmjopen.bmj.com/site/about/guidelines.xhtml

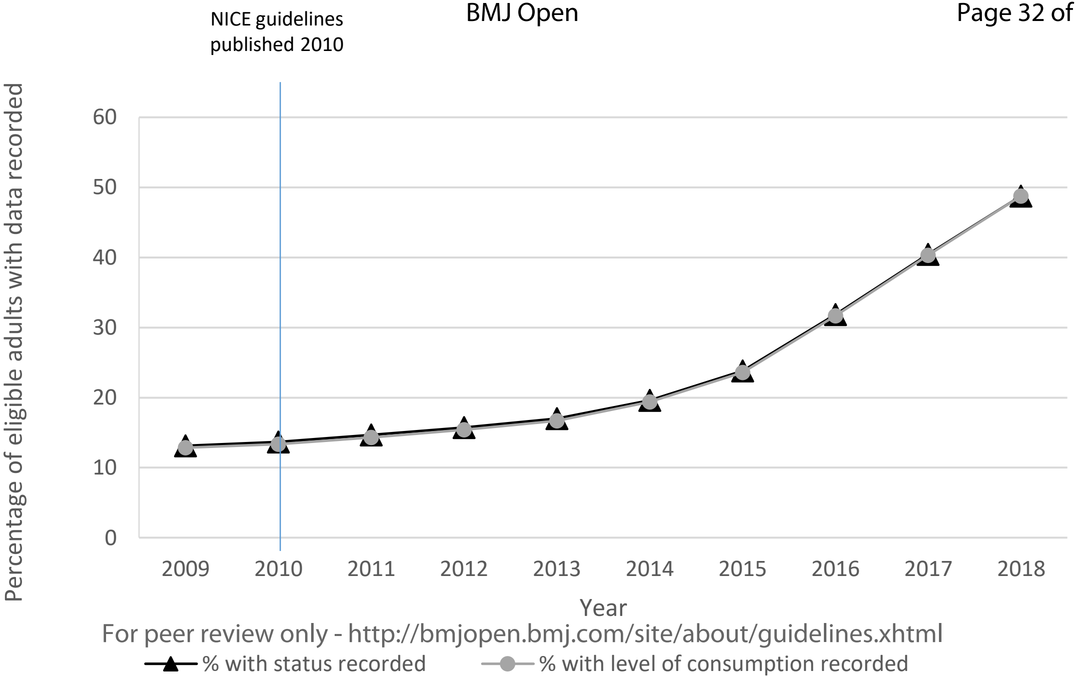

For peer review only - http://bmjopen.bmj.com/site/about/guidelines.xhtml

Appendices

Supplementary Text 1. Detailed alcohol-use definitions

i. Any code suggesting that alcohol was discussed in the consultation (**Supplementary Table 1**, Column 1).
ii. Codes indicating AUDIT or FAST screening (**Supplementary Table 1**, Columns 4 and 5)
iii. Codes quantifying alcohol use, comprised of:
    a. Drinking status (categorised as current drinker, ex drinker, non drinker; recorded in:
        - Read codes (**Supplementary Table 1**, Column 2)
        - Structured data, using data from the CPRD Additional Clinical Details file recorded under Entity Type 5, data field 1 (status) (Entity Type is the identifier that represents the structured data area in Vision where the data was entered)
    b. Level of drinking (non, light, moderate, heavy drinker; as recorded in Read codes (**Supplementary Table 1**, Column 3))
    c. Alcohol units consumed per week, using an existing (CALIBER: https://www.ucl.ac.uk/health-informatics/caliber) variable definition for 'number of alcohol unit consumption per week recorded in primary care (CPRD)',[1] using data from the CPRD Additional Clinical Details file recorded under Entity Type 5, data field 2 (units per week). We categorised units per week as: "None", "1-14, "15-42" or "43+" units per week. 14 units per week is the recommended limit for men and women according to current UK government guidelines.[2]
    d. AUDIT or FAST scores (AUDIT categorised according to WHO guidelines on risk level: 1-7 - low risk drinking; 8-15 - hazardous drinking; 16-19 - harmful drinking; 20-40 – possible dependence.[3]). Scores resulting from AUDIT/FAST screening (i.e. associated with a Read morbidity code indicating use of AUDIT/FAST), were obtained from the Additional Clinical Details file under Entity Type 172.

For peer review only

For peer review only - http://bmjopen.bmj.com/site/about/guidelines.xhtml

Supplementary Table 1. Read codes for alcohol data in CPRD (code lists also available for download: https://doi.org/10.17037/data.00001071).

CPRD medcode	Read code	Clinical term	1 Any alcohol-related code	2 Current drinking status	3 Codes indicating level of alcohol use	4 AUDIT screening	5 FAST screening
47555	90.95	cerebral degeneration due to alcoholism	1	0	0	0	0
27	136..00	alcohol consumption	1	0	0	0	0
12949	1361.00	Teetotaller	1	non	non	0	0
12970	1361.11	non drinker alcohol	1	non	non	0	0
4447	1361.12	non-drinker alcohol	1	non	non	0	0
12975	1362.00	trivial drinker - <1u/day	1	curr	L	0	0
385	1362.11	drinks rarely	1	curr	L	0	0
749	1362.12	drinks occasionally	1	curr	L	0	0
12972	1363.00	light drinker - 1-2u/day	1	curr	L	0	0
322	1364.00	moderate drinker - 3-6u/day	1	curr	M	0	0
1618	1365.00	heavy drinker - 7-9u/day	1	curr	H	0	0
12977	1366.00	very heavy drinker - >9u/day	1	curr	H	0	0
967	1367.00	stopped drinking alcohol	1	ex	non	0	0
12978	1368.00	alcohol consumption unknown	1	0	0	0	0
12976	1369.00	suspect alcohol abuse - denied	1	0	0	0	0
22933	136A.00	ex-trivial drinker (<1u/day)	1	0	0	0	0
102665	136a.00	increasing risk drinking	1	curr	H	0	0
26471	136B.00	ex-light drinker - (1-2u/day)	1	0	0	0	0
99877	136b.00	feels should cut down drinking	1	curr	H	0	0
19495	136C.00	ex-moderate drinker - (3-6u/d)	1	0	0	0	0
102448	136c.00	higher risk drinking	1	curr	H	0	0
19493	136D.00	ex-heavy drinker - (7-9u/day)	1	0	0	0	0
103230	136d.00	lower risk drinking	1	curr	M	0	0
12983	136E.00	ex-very heavy drinker-(>9u/d)	1	0	0	0	0
106560	136e.00	declines to state current alcohol consumption	1	0	0	0	0
12971	136F.00	spirit drinker	1	curr	0	0	0
2689	136G.00	beer drinker	1	curr	0	0	0
12968	136H.00	drinks beer and spirits	1	curr	0	0	0
12969	136I.00	drinks wine	1	curr	0	0	0
956	136J.00	social drinker	1	curr	M	0	0

For peer review only

CPRD medcode	Read code	Clinical term	1 Any alcohol-related code	2 Current drinking status	3 Codes indicating level of alcohol use	4 AUDIT screening	5 FAST screening
12982	136K.00	alcohol intake above recommended sensible limits	1	curr	H	0	0
26472	136L.00	alcohol intake within recommended sensible limits	1	curr	M	0	0
12979	136M.00	current non drinker	1	non	non	0	0
12980	136N.00	light drinker	1	curr	L	0	0
12985	136O.00	moderate drinker	1	curr	M	0	0
8999	136P.00	heavy drinker	1	curr	H	0	0
12984	136Q.00	very heavy drinker	1	curr	H	0	0
19401	136R.00	binge drinker	1	curr	H	0	0
19494	136S.00	hazardous alcohol use	1	curr	H	0	0
30695	136T.00	harmful alcohol use	1	curr	H	0	0
93415	136V.00	alcohol units per week	1	0	0	0	0
94670	136W.00	alcohol misuse	1	curr	H	0	0
97126	136X.00	alcohol units consumed on heaviest drinking day	1	0	0	0	0
101718	136Y.00	drinks in morning to get rid of hangover	1	curr	0	0	0
12981	136Z.00	alcohol consumption nos	1	0	0	0	0
18156	13Y8.00	alcoholics anonymous	1	0	0	0	0
84218	13ZY.00	disqualified from driving due to excess alcohol	1	0	0	0	0
8430	1462.00	h/o: alcoholism	1	0	0	0	0
38061	1B1c.00	alcohol induced hallucinations	1	curr	0	0	0
44783	1D19.00	pain in lymph nodes after alcohol consumption	1	0	0	0	0
24735	2577.00	o/e - breath - alcohol smell	1	curr	0	0	0
10161	2577.11	o/e - alcoholic breath	1	curr	0	0	0
69691	388j.00	cage questionnaire	1	0	0	0	0
70161	388j.00	cage questionnaire	1	0	0	0	0
93624	388u.00	fast alcohol screening test	1	0	0	0	1
96107	38D2.00	single alcohol screening questionnaire	1	0	0	0	0
94838	38D3.00	alcohol use disorders identification test	1	0	0	1	0
95744	38D4.00	alcohol use disorder identificatn test consumptn questionnre	1	0	0	1	0
97501	38D5.00	alcoh use disor id test piccinelli consumption questionnaire	1	0	0	1	0
100493	38Df.00	five-shot questionnaire on heavy drinking	1	0	0	0	0
102577	38Dz.00	severity of alcohol dependence questionnaire	1	0	0	0	0
102770	38Dz.11	sadq - severity of alcohol dependence questionnaire	1	0	0	0	0

For peer review only

CPRD medcode	Read code	Clinical term	1 Any alcohol-related code	2 Current drinking status	3 Codes indicating level of alcohol use	4 AUDIT screening	5 FAST screening
100989	4I91.11	breath alcohol level	1	0	0	0	0
17266	63C7.00	maternal alcohol abuse	1	0	0	0	0
12442	66e..00	alcohol disorder monitoring	1	curr	0	0	0
32964	66e0.00	alcohol abuse monitoring	1	0	0	0	0
11491	6792.00	health ed. - alcohol	1	0	0	0	0
35859	67A5.00	pregnancy alcohol advice	1	0	0	0	0
18711	67H0.00	lifestyle advice regarding alcohol	1	0	0	0	0
105144	67K6.00	cycle of change stage, alcohol	1	0	0	0	0
19489	6892.00	alcohol consumption screen	1	0	0	0	0
9264	68S..00	alcohol consumption screen	1	0	0	0	0
56410	7P22100	delivery of rehabilitation for alcohol addiction	1	0	0	0	0
2083	8BA8.00	alcohol detoxification	1	0	0	0	0
7692	8CAM.00	patient advised about alcohol	1	0	0	0	0
102564	8CAM000	advised to abstain from alcohol consumption	1	0	0	0	0
97309	8CAv.00	advised to contact primary care alcohol worker	1	curr	0	0	0
37264	8CE1.00	alcohol leaflet given	1	0	0	0	0
29691	8G32.00	aversion therapy - alcoholism	1	0	0	0	0
21650	8H35.00	admitted to alcohol detoxification centre	1	0	0	0	0
9849	8H7p.00	referral to community alcohol team	1	0	0	0	0
12554	8HHe.00	referral to community drug and alcohol team	1	0	0	0	0
94553	8HkG.00	referral to specialist alcohol treatment service	1	0	0	0	0
96993	8HkJ.00	referral to alcohol brief intervention service	1	0	0	0	0
94669	8IA7.00	alcohol consumption screening test declined	1	0	0	0	0
97261	8IAF.00	brief intervention for excessive alcohol consumptn declined	1	curr	0	0	0
97680	8IAJ.00	declined referral to specialist alcohol treatment service	1	curr	0	0	0
102247	8IAt.00	extended interven for excessive alcohol consumption declined	1	curr	0	0	0
103459	8IEA.00	referral to community alcohol team declined	1	curr	0	0	0
43813	9EQ..11	police:venesect-alcohol	1	0	0	0	0
102121	9EQ..12	police:venesect-alcohol	1	0	0	0	0
11740	9k1..00	alcohol misuse - enhanced services administration	1	curr	0	0	0
35330	9k11.00	alcohol consumption counselling	1	curr	0	0	0
63529	9k12.00	alcohol misuse - enhanced service completed	1	0	0	0	0

For peer review only - http://bmjopen.bmj.com/site/about/guidelines.xhtml

For peer review only

CPRD medcode	Read code	Clinical term	1 Any alcohol-related code	2 Current drinking status	3 Codes indicating level of alcohol use	4 AUDIT screening	5 FAST screening
48545	9k13.00	alcohol questionnaire completed	1	0	0	0	0
47123	9k14.00	alcohol counselling by other agencies	1	0	0	0	0
90714	9k15.00	alcohol screen - audit completed	1	0	0	1	0
94963	9k16.00	alcohol screen - fast alcohol screening test completed	1	0	0	0	1
95663	9k17.00	alcohol screen - audit c completed	1	0	0	1	0
94485	9k18.00	alcohol screen - audit pc completed	1	0	0	1	0
95944	9k19.00	alcohol assesment declined - enhanced services admin	1	0	0	0	0
96259	9k19.11	alcohol assessment declined	1	0	0	0	0
96053	9k1A.00	brief intervention for excessive alcohol consumptn completed	1	0	0	0	0
96054	9k1B.00	extended intervention for excessive alcohol consumptn complt	1	0	0	0	0
9489	9NN2.00	under care of community alcohol team	1	0	0	0	0
65754	C150500	alcohol-induced pseudo-cushing's syndrome	1	0	0	0	0
16237	E01..00	alcoholic psychoses	1	0	0	0	0
16225	E010.00	alcohol withdrawal delirium	1	curr	0	0	0
22277	E010.11	dts - delirium tremens	1	curr	0	0	0
1476	E010.12	Delirium tremens	1	curr	0	0	0
20762	E011.00	alcohol amnestic syndrome	1	0	0	0	0
4500	E011000	korsakov's alcoholic psychosis	1	0	0	0	0
11106	E011100	korsakov's alcoholic psychosis with peripheral neuritis	1	0	0	0	0
18636	E011200	wernicke-korsakov syndrome	1	0	0	0	0
41920	E011z00	alcohol amnestic syndrome nos	1	0	0	0	0
54505	E012.00	other alcoholic dementia	1	0	0	0	0
27342	E012.11	alcoholic dementia nos	1	0	0	0	0
37946	E012000	chronic alcoholic brain syndrome	1	0	0	0	0
25110	E013.00	alcohol withdrawal hallucinosis	1	curr	0	0	0
57939	E014.00	pathological alcohol intoxication	1	curr	0	0	0
20407	E014.11	drunkenness - pathological	1	curr	0	0	0
30404	E015.00	alcoholic paranoia	1	curr	0	0	0
33670	E01y.00	other alcoholic psychosis	1	0	0	0	0
2082	E01y000	alcohol withdrawal syndrome	1	curr	0	0	0
68111	E01yz00	other alcoholic psychosis nos	1	0	0	0	0
67651	E01z.00	alcoholic psychosis nos	1	0	0	0	0

For peer review only - http://bmjopen.bmj.com/site/about/guidelines.xhtml
For peer review only

CPRD medcode	Read code	Clinical term	1 Any alcohol-related code	2 Current drinking status	3 Codes indicating level of alcohol use	4 AUDIT screening	5 FAST screening
2084	E23..00	alcohol dependence syndrome	1	curr	0	0	0
2081	E23..11	alcoholism	1	curr	0	0	0
1399	E23..12	alcohol problem drinking	1	curr	H	0	0
5740	E230.00	acute alcoholic intoxication in alcoholism	1	curr	0	0	0
57714	E230.11	alcohol dependence with acute alcoholic intoxication	1	curr	0	0	0
40530	E230000	acute alcoholic intoxication, unspecified, in alcoholism	1	curr	0	0	0
56947	E230100	continuous acute alcoholic intoxication in alcoholism	1	curr	0	0	0
21624	E230200	episodic acute alcoholic intoxication in alcoholism	1	curr	0	0	0
59574	E230300	acute alcoholic intoxication in remission, in alcoholism	1	0	0	0	0
36296	E230z00	acute alcoholic intoxication in alcoholism nos	1	curr	0	0	0
31443	E231.00	chronic alcoholism	1	curr	0	0	0
37605	E231.11	Dipsomania	1	curr	0	0	0
43193	E231000	unspecified chronic alcoholism	1	curr	0	0	0
24064	E231100	continuous chronic alcoholism	1	curr	0	0	0
26106	E231200	episodic chronic alcoholism	1	curr	0	0	0
24485	E231300	chronic alcoholism in remission	1	0	0	0	0
33635	E231z00	chronic alcoholism nos	1	curr	0	0	0
6169	E23z.00	alcohol dependence syndrome nos	1	curr	0	0	0
7746	E250.00	nondependent alcohol abuse	1	curr	H	0	0
12271	E250.11	Drunkenness NOS	1	curr	0	0	0
27518	E250.12	hangover (alcohol)	1	curr	0	0	0
17777	E250.13	Inebriety NOS	1	curr	0	0	0
3782	E250.14	intoxication - alcohol	1	curr	0	0	0
669	E250000	nondependent alcohol abuse, unspecified	1	curr	H	0	0
23610	E250100	nondependent alcohol abuse, continuous	1	curr	H	0	0
12974	E250200	nondependent alcohol abuse, episodic	1	curr	H	0	0
31569	E250300	nondependent alcohol abuse in remission	1	0	0	0	0
28150	E250z00	nondependent alcohol abuse nos	1	curr	H	0	0
5611	Eu10.00	[x]mental and behavioural disorders due to use of alcohol	1	0	0	0	0
44299	Eu10000	[x]mental & behav dis due to use alcohol: acute intoxication	1	curr	0	0	0
9508	Eu10011	[x]acute alcoholic drunkenness	1	curr	0	0	0
21879	Eu10100	[x]mental and behav dis due to use of alcohol: harmful use	1	curr	0	0	0

For peer review only - http://bmjopen.bmj.com/site/about/guidelines.xhtml

CPRD medcode	Read code	Clinical term	1 Any alcohol-related code	2 Current drinking status	3 Codes indicating level of alcohol use	4 AUDIT screening	5 FAST screening
39327	Eu10200	[x]mental and behav dis due to use alcohol: dependence syndr	1	curr	0	0	0
28780	Eu10211	[x]alcohol addiction	1	curr	0	0	0
5758	Eu10212	[x]chronic alcoholism	1	curr	0	0	0
20514	Eu10300	[x]mental and behav dis due to use alcohol: withdrawal state	1	curr	0	0	0
64101	Eu10400	[X]Men & behav dis due alcohl: withdrawl state with delirium	1	curr	0	0	0
17259	Eu10411	[x]delirium tremens, alcohol induced	1	curr	0	0	0
12353	Eu10500	[x]mental & behav dis due to use alcohol: psychotic disorder	1	0	0	0	0
6467	Eu10511	[x]alcoholic hallucinosis	1	curr	0	0	0
65932	Eu10512	[x]alcoholic jealousy	1	curr	0	0	0
30162	Eu10513	[x]alcoholic paranoia	1	curr	0	0	0
17607	Eu10514	[x]alcoholic psychosis nos	1	0	0	0	0
39799	Eu10600	[x]mental and behav dis due to use alcohol: amnesic syndrome	1	0	0	0	0
11670	Eu10611	[x]korsakov's psychosis, alcohol induced	1	0	0	0	0
62000	Eu10700	[X]Men & behav dis due alcoh: resid & late-onset psychot dis	1	0	0	0	0
26323	Eu10711	[x]alcoholic dementia nos	1	0	0	0	0
37691	Eu10712	[x]chronic alcoholic brain syndrome	1	0	0	0	0
32927	Eu10800	[x]alcohol withdrawal-induced seizure	1	curr	0	0	0
45169	Eu10y00	[x]men & behav dis due to use alcohol: oth men & behav dis	1	0	0	0	0
64389	Eu10z00	[x]ment & behav dis due use alcohol: unsp ment & behav dis	1	0	0	0	0
36748	F11x011	alcoholic encephalopathy	1	0	0	0	0
33839	F144000	cerebellar ataxia due to alcoholism	1	0	0	0	0
30604	F25B.00	alcohol-induced epilepsy	1	0	0	0	0
2925	F375.00	alcoholic polyneuropathy	1	0	0	0	0
31742	F394100	alcoholic myopathy	1	0	0	0	0
4915	G555.00	alcoholic cardiomyopathy	1	0	0	0	0
8363	G852300	oesophageal varices in alcoholic cirrhosis of the liver	1	0	0	0	0
4506	J153.00	alcoholic gastritis	1	0	0	0	0
10691	J610.00	alcoholic fatty liver	1	0	0	0	0
3216	J611.00	acute alcoholic hepatitis	1	0	0	0	0
4743	J612.00	alcoholic cirrhosis of liver	1	0	0	0	0
21713	J612000	alcoholic fibrosis and sclerosis of liver	1	0	0	0	0
7885	J613.00	alcoholic liver damage unspecified	1	0	0	0	0

For peer review only - http://bmjopen.bmj.com/site/about/guidelines.xhtml
For peer review only

CPRD medcode	Read code	Clinical term	1 Any alcohol-related code	2 Current drinking status	3 Codes indicating level of alcohol use	4 AUDIT screening	5 FAST screening
17330	J613000	alcoholic hepatic failure	1	0	0	0	0
7943	J617.00	alcoholic hepatitis	1	0	0	0	0
7602	J617000	chronic alcoholic hepatitis	1	0	0	0	0
104611	J670800	alcohol-induced acute pancreatitis	1	0	0	0	0
24984	J671000	alcohol-induced chronic pancreatitis	1	0	0	0	0
66019	L254.11	suspect fetal damage from maternal alcohol	1	0	0	0	0
27670	L255300	maternal care for (suspected) damage to fetus from alcohol	1	0	0	0	0
72757	Q007100	fetus/neonate affected by placental/breast transfer alcohol	1	0	0	0	0
97916	Q007111	fetal alcohol syndrome	1	0	0	0	0
9169	R103.00	[d]alcohol blood level excessive	1	0	0	0	0
36687	SLH3.00	alcohol deterrent poisoning	1	0	0	0	0
19217	SM0..00	alcohol causing toxic effect	1	0	0	0	0
8984	SM00.00	ethyl alcohol causing toxic effect	1	0	0	0	0
48514	SM00100	denatured alcohol causing toxic effect	1	0	0	0	0
55536	SM00z00	ethyl alcohol causing toxic effect nos	1	0	0	0	0
88997	SM01.00	methyl alcohol causing toxic effect	1	0	0	0	0
11263	SM01100	wood alcohol causing toxic effect	1	0	0	0	0
106018	SM02.00	isopropyl alcohol causing toxic effect	1	0	0	0	0
102086	SM02200	rubbing alcohol causing toxic effect	1	0	0	0	0
64396	SM03000	amyl alcohol causing toxic effect	1	0	0	0	0
102321	SM03100	butyl alcohol causing toxic effect	1	0	0	0	0
101543	SM0y.00	other alcohol causing toxic effect	1	0	0	0	0
36499	SM0z.00	alcohol causing toxic effect nos	1	0	0	0	0
95410	SyuG000	[x]toxic effect of other alcohols	1	0	0	0	0
18252	T90..00	accidental poisoning by alcohol, nec	1	0	0	0	0
40541	T900.00	accidental poisoning by alcoholic beverages	1	0	0	0	0
15503	T901.00	accidental poisoning by other ethyl alcohol and its products	1	0	0	0	0
101426	T901z00	accidental poisoning by ethyl alcohol nos	1	0	0	0	0
37174	T902.00	accidental poisoning by methyl alcohol	1	0	0	0	0
60752	T903300	accidental poisoning by secondary propyl alcohol	1	0	0	0	0
53139	T90y.00	accidental poisoning by other alcohols	1	0	0	0	0
55415	T90z.00	accidental poisoning by alcohol nos	1	0	0	0	0

For peer review only - http://bmjopen.bmj.com/site/about/guidelines.xhtml

For peer review only

CPRD medcode	Read code	Clinical term	1 Any alcohol-related code	2 Current drinking status	3 Codes indicating level of alcohol use	4 AUDIT screening	5 FAST screening
21412	TJH3.00	adverse reaction to alcohol deterrents	1	0	0	0	0
31605	U1A9.00	[x]accident poisoning/exposure to alcohol	1	0	0	0	0
63306	U1A9000	[x]accident poison/exposure to alcohol at home	1	0	0	0	0
103069	U1A9200	[x]acc poison/expos alcohol school/pub admin area	1	0	0	0	0
104702	U1A9300	[x]accid pois/expos alcohol in sport/athletic area	1	0	0	0	0
63876	U1A9400	[x]accid poison/expos alcohol in street/highway	1	0	0	0	0
92908	U1A9500	[x]accid poison/expos alcohol trade/service area	1	0	0	0	0
104734	U1A9y00	[x]accid pois/expos to alcohol other spec place	1	0	0	0	0
61187	U1A9z00	[x]accid poison/expos to alcohol unspecif place	1	0	0	0	0
10463	U209.00	[x]intent self poison/exposure to alcohol	1	0	0	0	0
41638	U209000	[x]int self poison/exposure to alcohol at home	1	0	0	0	0
97085	U209y00	[x]int self poison alcohol other spec place	1	0	0	0	0
59414	U209z00	[x]intent self poison alcohol unspecif place	1	0	0	0	0
30036	U409.00	[x]poisoning/exposure, ? intent, to alcohol	1	0	0	0	0
68159	U409000	[x]poison/exposure ?intent, to alcohol at home	1	0	0	0	0
69407	U409200	[x]pois/exp ?intent alcohol school/pub admin area	1	0	0	0	0
96219	U409400	[x]pois/expos ?intent alcohol in street/highway	1	0	0	0	0
61190	U409z00	[x]pois/expos ?intent to alcohol unspecif place	1	0	0	0	0
73876	U60H300	[x]alcohol deterrents caus adverse effects in therapeut use	1	0	0	0	0
48241	U60H311	[x] adverse reaction to alcohol deterrents	1	0	0	0	0
44019	U80..00	[x]evidence of alcohl involv determin by blood alcohl level	1	0	0	0	0
12950	U800.00	[x]eviden of alcohl involv blood alcohl level <20 mg/100 ml	1	0	0	0	0
57202	U801.00	[x]eviden of alcohl involv blood alcohl level 20-39mg/100ml	1	0	0	0	0
59079	U802.00	[x]eviden of alcohl involv blood alcohl level 40-59mg/100ml	1	0	0	0	0
57242	U803.00	[x]eviden of alcohl involv blood alcohl level 60-79mg/100ml	1	0	0	0	0
53428	U804.00	[x]eviden of alcohl involv blood alcohl level 80-99mg/100ml	1	0	0	0	0
54504	U805.00	[x]eviden of alcoh involv blood alcoh level 100-119mg/100ml	1	0	0	0	0
32454	U806.00	[x]eviden of alcoh involv blood alcoh level 120-199mg/100ml	1	0	0	0	0
47907	U807.00	[x]eviden of alcoh involv blood alcoh level 200-239mg/100ml	1	0	0	0	0
39726	U808.00	[x]eviden alcoh involv blood alcoh level 240mg/100ml or more	1	0	0	0	0
103698	U80z.00	[x]evid alcoh invol detrm by pres alcoh in bld levl not spec	1	0	0	0	0
23978	U81..00	[x]evid of alcohol involv determind by level of intoxication	1	0	0	0	0

For peer review only - http://bmjopen.bmj.com/site/about/guidelines.xhtml

CPRD medcode	Read code	Clinical term	1 Any alcohol-related code	2 Current drinking status	3 Codes indicating level of alcohol use	4 AUDIT screening	5 FAST screening
105273	U810.00	[x]evid alcoh invol determ by levl of intox mild alcoh intox	1	0	0	0	0
73480	U811.00	[x]evid alcoh invol determ by level of intox mod alcoh intox	1	0	0	0	0
44686	U812.00	[x]evid alcoh invol determ by level of intox sev alcoh intox	1	0	0	0	0
39738	U813.00	[x]evid alcoh invl determ by levl intox very sev alcoh intox	1	0	0	0	0
41983	Z191.00	alcohol detoxification	1	0	0	0	0
46677	Z191100	alcohol withdrawal regime	1	0	0	0	0
61383	Z191200	planned reduction of alcohol consumption	1	curr	0	0	0
95181	Z191211	alcohol reduction programme	1	curr	0	0	0
64409	Z191400	self-monitoring of alcohol intake	1	0	0	0	0
30460	Z4B1.00	alcoholism counselling	1	curr	0	0	0
21829	Z786200	drinking practice	1	0	0	0	0
95650	ZC22100	advice to change drink intake	1	curr	0	0	0
97163	ZC22200	advice to change alcoholic drink intake	1	curr	0	0	0
54209	ZC2H.00	advice to change alcohol intake	1	curr	0	0	0
11140	ZG23100	advice on alcohol consumption	1	0	0	0	0
50507	ZR1E.00	alcohol dependence scale	1	0	0	0	0
40602	ZR1E.11	ads - alcohol dependence scale	1	0	0	0	0
32850	ZR1F.00	alcohol use disorders identification test	1	0	0	1	0
59873	ZR1F.11	audit - alcohol use disorders identification test	1	0	0	1	0
65980	ZR1G.00	alcohol use inventory	1	0	0	0	0
45557	ZR31.00	cage questionnaire	1	0	0	0	0
61750	ZR3f.00	comprehensive drinker profile	1	0	0	0	0
63457	ZR3f.11	cdp - comprehensive drinker profile	1	0	0	0	0
56441	ZRa1.00	michigan alcoholism screening test	1	0	0	0	0
69331	ZRa1.11	mast - michigan alcoholism screening test	1	0	0	0	0
99985	ZRa1100	brief michigan alcoholism screening test	1	0	0	0	0
41251	ZRa1111	bmast - brief michigan alcoholism screening test	1	0	0	0	0
70939	ZRaU.00	munich alcoholism test	1	0	0	0	0
22707	ZRBJ.00	drinking problem scale	1	0	0	0	0
46848	ZRBJ.11	dps - drinking problem scale	1	0	0	0	0
42305	ZRk6.00	severity of alcohol dependence questionnaire	1	0	0	0	0
66831	ZRk6.11	sadq - severity of alcohol dependence questionnaire	1	0	0	0	0

For peer review only - http://bmjopen.bmj.com/site/about/guidelines.xhtml

For peer review only

CPRD medcode	Read code	Clinical term	1 Any alcohol-related code	2 Current drinking status	3 Codes indicating level of alcohol use	4 AUDIT screening	5 FAST screening
62300	ZRk9.00	short alcohol dependence data	1	0	0	0	0
62299	ZRk9.11	sadd - short alcohol dependence data	1	0	0	0	0
10658	ZRLfD12	honos item 3 - alcohol/drug problem	1	0	0	0	0
66552	ZRR..00	inventory of drinking situations	1	0	0	0	0
59776	ZRVK.00	last six months of drinking questionnaire	1	0	0	0	0
7123	ZV11300	[v]personal history of alcoholism	1	0	0	0	0
16587	ZV11311	[v]problems related to lifestyle alcohol use	1	curr	H	0	0
7545	ZV4KC00	[v] alcohol use	1	0	0	0	0
8388	ZV57A00	[v]alcohol rehabilitation	1	0	0	0	0
8030	ZV6D600	[v]alcohol abuse counselling and surveillance	1	curr	0	0	0
61583	ZV70411	[v]medicolegal blood alcohol test	1	0	0	0	0
17149	ZV70L00	[v]blood-alcohol and blood-drug test	1	0	0	0	0
10458	ZV79100	[v]screening for alcoholism	1	0	0	0	0

For peer review only

Supplementary Table 2. Internal validity comparing alcohol consumption recorded up to 30 days before or after an AUDIT score, AUDIT-C score or measure of units per week.

	N	(%)	N patients with AUDIT score on the same date as record n	(%)	AUDIT score median	(IQR)	N patients with AUDIT C score on the same date as record n	(%)	AUDIT C score median	(IQR)	N patients with units per week recorded on same date as record n	(%)	Units per week median	(IQR)
**Current drinking status***														
Non	174,970	(20.3)	3053	(1.7)	0	(0-0)	1096	(0.6)	0	(0-0)	882	(0.5)	0	(0-1)
Ex	31,526	(3.7)	326	(1)	0	(0-0)	43	(0.1)	0	(0-0)	999	(3.2)	0	(0-1)
Current	655,834	(76.1)	24402	(3.7)	3	(1-5)	4707	(0.7)	3	(2-5)	333,402	(50.8)	6	(2-14)
**Current drinking level (Read codes)**														
Non-drinker	222835	(39.9)	3437	(1.5)	0	(0-0)	1145	(0.5)	0	(0-0)	2,715	(1.2)	0	(0-2)
Light drinker	255,919	(45.8)	3,401	(1.3)	2	(1-3)	1,131	(0.4)	2	(1-3)	19,777	(7.7)	3	(1-8)
Moderate drinker	52,070	(9.3)	1,205	(2.3)	1	(0-4)	236	(0.5)	3	(2-4)	13,024	(25)	6	(2-14)
Heavy drinker	27,923	(5)	2,906	(10.4)	6	(5-9)	127	(0.5)	8	(6-9)	10,572	(37.9)	18	(10-30)
**Units per week**														
0	38,929	(9)	1,919	(4.9)	0	(0-0)	159	(0.4)	0	(0-1)				
1-14	298,441	(69.2)	11,920	(4)	3	(2-4)	2,273	(0.8)	3	(2-4)	n/a		n/a	
15-42	80,449	(18.6)	2,983	(3.7)	6	(4-8)	412	(0.5)	5	(4-7)				
43+	13,575	(3.1)	248	(1.8)	10	(8-12)	19	(0.1)	8	(5-10)				
**AUDIT C score category**														
0-4; Low risk	16,206	(76.8)	4,006	(24.7)	2	(0-3)	n/a		n/a		2,120	(13.1)	4	(2-8)
5-12; High risk	4,893	(23.2)	1,160	(23.7)	6	(5-7)					901	(18.4)	12	(7-20)
**AUDIT score category**														
0-7; Alcohol Education	71,401	(92.5)					4,899	(6.9)	2	(1-4)	15,852	(22.2)	4	(2-10)
8-15; Simple Advice	5,405	(7)					242	(4.5)	9	(8-10)	1,605	(29.7)	20	(14-30)
16-19; Simple Advice plus Brief Counseling and Continued Monitoring	206	(0.3)	n/a		n/a		0	(0)		(-)	49	(23.8)	24	(16-48)
20-40; Referral to Specialist for Diagnostic Evaluation and Treatment	200	(0.3)					0	(0)		(-)	30	(15)	47	(20-80)

*based on recorded status in Read codes and Additional details (not including derived status based on units consumed or AUDIT score)

For peer review only - http://bmjopen.bmj.com/site/about/guidelines.xhtml

References

1. Denaxas, S.C., et al., *Data resource profile: cardiovascular disease research using linked bespoke studies and electronic health records (CALIBER).* Int J Epidemiol, 2012. **41**(6): p. 1625-38.

2. Department of Health, *UK Chief Medical Officers' alcohol guidelines review: Summary of the proposed new guidelines.* 2016.

3. Babor, T.F., et al. *The Alcohol Use Disorders Identification Test: Guidelines for Use in Primary Care*. 2001; Available from: http://www.talkingalcohol.com/files/pdfs/WHO_audit.pdf.

For peer review only

For peer review only - http://bmjopen.bmj.com/site/about/guidelines.xhtml

The RECORD statement – checklist of items, extended from the STROBE statement, that should be reported in observational studies using routinely collected health data.

	Item No.	STROBE items	Location in manuscript where items are reported	RECORD items	Location in manuscript where items are reported
**Title and abstract**					
	1	(a) Indicate the study's design with a commonly used term in the title or the abstract (b) Provide in the abstract an informative and balanced summary of what was done and what was found	Page 1	RECORD 1.1: The type of data used should be specified in the title or abstract. When possible, the name of the databases used should be included.<br><br>RECORD 1.2: If applicable, the geographic region and timeframe within which the study took place should be reported in the title or abstract.<br><br>RECORD 1.3: If linkage between databases was conducted for the study, this should be clearly stated in the title or abstract.	Page 2<br><br>Page 2<br><br>n/a
**Introduction**					
Background rationale	2	Explain the scientific background and rationale for the investigation being reported	Page 4		
Objectives	3	State specific objectives, including any prespecified hypotheses	Page 5		
**Methods**					
Study Design	4	Present key elements of study design early in the paper	Page 6		
Setting	5	Describe the setting, locations, and relevant dates, including periods of recruitment, exposure, follow-up, and data collection	Page 6		

For peer review only

Participants	6	*(a) Cohort study* - Give the eligibility criteria, and the sources and methods of selection of participants. Describe methods of follow-up<br>*Case-control study* - Give the eligibility criteria, and the sources and methods of case ascertainment and control selection. Give the rationale for the choice of cases and controls<br>*Cross-sectional study* - Give the eligibility criteria, and the sources and methods of selection of participants<br><br>*(b) Cohort study* - For matched studies, give matching criteria and number of exposed and unexposed<br>*Case-control study* - For matched studies, give matching criteria and the number of controls per case	Page 6	RECORD 6.1: The methods of study population selection (such as codes or algorithms used to identify subjects) should be listed in detail. If this is not possible, an explanation should be provided.<br><br>RECORD 6.2: Any validation studies of the codes or algorithms used to select the population should be referenced. If validation was conducted for this study and not published elsewhere, detailed methods and results should be provided.<br><br>RECORD 6.3: If the study involved linkage of databases, consider use of a flow diagram or other graphical display to demonstrate the data linkage process, including the number of individuals with linked data at each stage.	Page 6
Variables	7	Clearly define all outcomes, exposures, predictors, potential confounders, and effect modifiers. Give diagnostic criteria, if applicable.	Page 7	RECORD 7.1: A complete list of codes and algorithms used to classify exposures, outcomes, confounders, and effect modifiers should be provided. If these cannot be reported, an explanation should be provided.	Appendices and online via link
Data sources/ measurement	8	For each variable of interest, give sources of data and details of methods of assessment (measurement).<br>Describe comparability of assessment methods if there is more than one group	Page 7, online via link on page 7		

For peer review only

Bias	9	Describe any efforts to address potential sources of bias	Discussion		
Study size	10	Explain how the study size was arrived at	Appendix Figure 1		
Quantitative variables	11	Explain how quantitative variables were handled in the analyses. If applicable, describe which groupings were chosen, and why	Page 6, page 11		
Statistical methods	12	(a) Describe all statistical methods, including those used to control for confounding<br>(b) Describe any methods used to examine subgroups and interactions<br>(c) Explain how missing data were addressed<br>(d) *Cohort study* - If applicable, explain how loss to follow-up was addressed<br>*Case-control study* - If applicable, explain how matching of cases and controls was addressed<br>*Cross-sectional study* - If applicable, describe analytical methods taking account of sampling strategy<br>(e) Describe any sensitivity analyses	Pages 8-9		
Data access and cleaning methods		..		RECORD 12.1: Authors should describe the extent to which the investigators had access to the database population used to create the study population.	Page 24

For peer review only

				RECORD 12.2: Authors should provide information on the data cleaning methods used in the study.	
Linkage		..		RECORD 12.3: State whether the study included person-level, institutional-level, or other data linkage across two or more databases. The methods of linkage and methods of linkage quality evaluation should be provided.	n/a
**Results**					
Participants	13	(a) Report the numbers of individuals at each stage of the study (*e.g.*, numbers potentially eligible, examined for eligibility, confirmed eligible, included in the study, completing follow-up, and analysed)<br>(b) Give reasons for non-participation at each stage.<br>(c) Consider use of a flow diagram		RECORD 13.1: Describe in detail the selection of the persons included in the study (*i.e.,* study population selection) including filtering based on data quality, data availability and linkage. The selection of included persons can be described in the text and/or by means of the study flow diagram.	Page 6
Descriptive data	14	(a) Give characteristics of study participants (*e.g.*, demographic, clinical, social) and information on exposures and potential confounders<br>(b) Indicate the number of participants with missing data for each variable of interest<br>(c) *Cohort study* - summarise follow-up time (*e.g.*, average and total amount)	Table 1<br><br>Table 1		
Outcome data	15	*Cohort study* - Report numbers of outcome events or summary measures over time<br>*Case-control study* - Report numbers in each exposure	Table 1		

		category, or summary measures of exposure *Cross-sectional study* - Report numbers of outcome events or summary measures			
Main results	16	(a) Give unadjusted estimates and, if applicable, confounder-adjusted estimates and their precision (e.g., 95% confidence interval). Make clear which confounders were adjusted for and why they were included (b) Report category boundaries when continuous variables were categorized (c) If relevant, consider translating estimates of relative risk into absolute risk for a meaningful time period	n/a		
Other analyses	17	Report other analyses done—e.g., analyses of subgroups and interactions, and sensitivity analyses	Pages 13-15		
**Discussion**					
Key results	18	Summarise key results with reference to study objectives	Page 16		
Limitations	19	Discuss limitations of the study, taking into account sources of potential bias or imprecision. Discuss both direction and magnitude of any potential bias	Page 19	RECORD 19.1: Discuss the implications of using data that were not created or collected to answer the specific research question(s). Include discussion of misclassification bias, unmeasured confounding, missing data, and changing eligibility over time, as they pertain to the study being reported.	Page 19
Interpretation	20	Give a cautious overall interpretation of results considering objectives,	Pages 20		

		limitations, multiplicity of analyses, results from similar studies, and other relevant evidence			
Generalisability	21	Discuss the generalisability (external validity) of the study results	Page 20		
**Other Information**					
Funding	22	Give the source of funding and the role of the funders for the present study and, if applicable, for the original study on which the present article is based	Page 24		
Accessibility of protocol, raw data, and programming code		..		RECORD 22.1: Authors should provide information on how to access any supplemental information such as the study protocol, raw data, or programming code.	Page 24

*Reference: Benchimol EI, Smeeth L, Guttmann A, Harron K, Moher D, Petersen I, Sørensen HT, von Elm E, Langan SM, the RECORD Working Committee. The REporting of studies Conducted using Observational Routinely-collected health Data (RECORD) Statement. *PLoS Medicine* 2015; in press.

*Checklist is protected under Creative Commons Attribution (CC BY) license.

For peer review only

Completeness and validity of alcohol recording in general practice within the UK: a cross-sectional study

Journal:	*BMJ Open*
Manuscript ID	bmjopen-2019-031537.R2
Article Type:	Original research
Date Submitted by the Author:	26-Sep-2019

Complete List of Authors:	Mansfield, Kathryn; London School of Hygiene and Tropical Medicine, Non-communicable Disease Epidemiology<br>Crellin, Elizabeth; Clinical Practice Research Datalink<br>Denholm, Rachel; University of Bristol, Centre for Academic Primary Care<br>Quint, Jennifer; Imperial College London, Respiratory Epidemiology, Occupational Medicine and Public Health<br>Smeeth, Liam; London School of Hygiene and Tropical Medicine, Epidemiology and Population Health<br>Cook, Sarah; London School of Hygiene & Tropical Medicine,<br>Herrett, Emily; London School of Hygiene and Tropical Medicine, Non communicable disease epidemiology

**Primary Subject Heading**:	Epidemiology
Secondary Subject Heading:	General practice / Family practice
Keywords:	alcohol, general practice, recording, validity, screening

SCHOLARONE™
Manuscripts

Completeness and validity of alcohol recording in general practice within the UK: a cross-sectional study

Kathryn Mansfield[1*], Elizabeth Crellin[2*], Rachel Denholm[3], Jennifer Quint[4], Liam Smeeth[1], Sarah Cook[1], Emily Herrett[1]

* Joint first authors.

1 - Department of Non-Communicable Diseases Epidemiology
London School of Hygiene and Tropical Medicine
London, UK

2 – Clinical Practice Research Datalink
3 – Centre for Academic Primary Care, Bristol Medical School,
University of Bristol,
Bristol, UK
4 – Imperial College London

Correspondence:
Dr Emily Herrett
Department of Non-Communicable Diseases Epidemiology
London School of Hygiene and Tropical Medicine
London UK
emily.herrett@lshtm.ac.uk

Word count 4644

For peer review only

Abstract

Background: Since 2010 the National Institute for Health and Care Excellence has recommended screening adults for excessive alcohol consumption to try and help prevent alcohol-use disorders. Little is known about the extent to which these recommendations are followed, and the resulting completeness and validity of alcohol-related data recording in primary care.

Objective: To investigate the completeness and accuracy of recording of alcohol use within primary care records in the UK.

Design and setting: Cross-sectional study in the Clinical Practice Research Datalink

Participants: We included all adult patients registered on 1st January 2018 with ≥1 year of follow-up.

Primary and secondary outcome measures: We calculated prevalence of alcohol consumption recording overall and within patient groups. We then validated alcohol consumption data against recommended screening tools (AUDIT) as the gold standard. We also calculated how prevalence of alcohol recording changed over the preceding decade.

Results: In 2018, among 1.8 million registered adult patients, just over half (51.9%) had a record for a code related to alcohol in the previous five years. Recording of alcohol consumption was more common among women, older people, ex-smokers, and those from more deprived areas, who were overweight/obese, or with comorbidities. A quarter of patients had units per week recorded in the last five years, but <10% had an AUDIT or FAST alcohol screening test score. The recorded alcohol measures corresponded to results from gold standard AUDIT scores. The distribution of consumption among current drinkers was similar to the Health Survey for England.

Conclusions: Half of adults in UK primary care have no recorded alcohol consumption data. When consumption is recorded, we have demonstrated internal and external validity of the data, suggesting greater recording may help identify opportunities for interventions to reduce harms.

For peer review only - http://bmjopen.bmj.com/site/about/guidelines.xhtml

Strengths and limitations of the study

- This study investigated alcohol recording across nearly 1.8 million UK adults in primary care, providing the power to understand in detail how alcohol use is recorded in general practice.
- We demonstrated the validity of alcohol recording internally within general practice records, and externally compared to the Health Survey for England.
- Though we demonstrated levels of alcohol recording and screening, it does not mean that GPs have acted upon the screening result, or that they have screened at an appropriate time.

For peer review only

For peer review only - http://bmjopen.bmj.com/site/about/guidelines.xhtml

Introduction

Hazardous alcohol use is a key behavioural risk factor affecting health. It is estimated that over 20% of adults in England are drinking at levels that are considered harmful ,[1] with a resulting cost to the NHS of over £3 billion per year.[2] In the age group 15-49 years, alcohol is the leading risk factor for ill health, early death and disability.[3]

Identification of high-risk patients can usefully be undertaken in primary care, where appropriate interventions can be promoted. The Alcohol Use Disorders Identification Test (AUDIT) [4] is the main tool for alcohol screening recommended by National Institute for Health and Care Excellence (NICE), though shortened versions (such as AUDIT-C and FAST) are also considered acceptable.[5] National Institute for Health and Care Excellence (NICE) guidelines published in 2010 recommend that opportunistic screening of the general population should take place during new patient registrations, and when screening or managing other conditions, promoting sexual health, during antenatal appointments and treating minor injuries. In addition, guidelines recommend screening 'high risk' groups, including those with relevant physical conditions (such as hypertension) and relevant mental health problems (such as depression).[5] Additionally, the NHS health check, which is offered to all people in the UK aged 40-74 years every five years, includes the AUDIT questions.[6]

Khadjesari et al (2013) showed relatively high levels of alcohol recording (76%) among new registrants in primary care between 2007 and 2009, although the use of validated screening tools was low (9%). They did not report levels for the whole registered patient population, which includes those who are less mobile and not subject to new patient registrations, nor specifically among patients with conditions that may be associated with, or exacerbated by, high-risk drinking. Poor recording of alcohol use has two major impacts: first and most importantly, patients may not be receiving appropriate interventions; and secondly, for

For peer review only - http://bmjopen.bmj.com/site/about/guidelines.xhtml

researchers, poor recording may prohibit reliable studies of the impact of alcohol on health using these data. Indeed, some recent studies using alcohol data in a large UK-based primary care electronic health record database (Clinical Practice Research Datalink [CPRD]) have indicated that bias may have arisen due to poor recording of alcohol data.[7, 8] For researchers using the data it is important to know not just whether screening occurs but if there are particular sub-groups more likely to be screened and to what extent alcohol codes used are meaningful reflections of actual alcohol use.

Using primary care data from the CPRD, this study assessed the utility of recorded alcohol data in primary care by assessing completeness of recording of alcohol status at different levels and by various patient level factors, and comparing measures of alcohol recorded in the CPRD internally and to external sources. Our study also offers insight into the completeness of alcohol data in primary care since the introduction of 2010 NICE guidance,[5] including specifically among those identified as being at higher risk according to NICE recommendations, and provides guidance for future studies related to alcohol use using primary care data.

For peer review only

For peer review only - http://bmjopen.bmj.com/site/about/guidelines.xhtml

Methods

Study design and setting

We undertook a cross-sectional study with data from the Clinical Practice Research Datalink GOLD database, an ongoing primary care database containing the anonymised medical records from general practitioners in the UK using the Vision software to capture health record data with coverage of 15.4 million patients. Data from CPRD includes diagnoses, tests, clinical measurements, prescriptions and specialist referrals. CPRD uses Read morbidity coding to summarise each patient encounter with a code (or codes) that correspond to a standard set of clinical terminology. Roughly half of all practices in the CPRD have consented to data linkage and therefore deprivation data (based on quintiles of the Indices of Multiple Deprivation (IMD)) is available for these practices. The study included all patients in CPRD GOLD who were 18 years or over, alive and registered, for at least one year, with a CPRD GOLD practice on 1st January 2018.

Factors associated with alcohol recording

We investigated a range of potential factors that may be associated with alcohol-use recording in primary care: age, sex, deprivation, geographic region, selected comorbidities related to alcohol use that could be captured in electronic health record data (liver disease, hypertension, depression and anxiety) or those where health function monitoring is likely to be more frequent/complete (diabetes mellitus), time since registration at the current practice (1-5 years vs ≥5 years), body mass index (BMI), smoking status, and ethnicity.

We identified liver disease, hypertension and diabetes mellitus based on presence of a recorded diagnosis at any time prior to the study date. We identified depression and anxiety based on the presence of a diagnosis at any time within the five years prior to the study date (1st January 2018). We used existing morbidity code lists and algorithms to define smoking status, BMI and ethnicity.[9, 10]

For peer review only - http://bmjopen.bmj.com/site/about/guidelines.xhtml

Outcomes

Our main outcomes were the prevalence of alcohol-use recording in the five years prior to the study date, 1st January 2018. Prevalence of alcohol recording included any record of alcohol use or its effects, and we also broke it down into the following categories:

i. Any code suggesting that alcohol was discussed in the consultation.
ii. Codes indicating AUDIT (including short-form AUDIT-C and full AUDIT) or FAST screening.
iii. Codes quantifying alcohol use, comprised of:
    a. drinking status (current drinker, ex, non; either as recorded in Read codes, or in structured data),
    b. level of drinking (non, light, moderate, heavy drinker; as recorded in Read codes),
    c. units per week,
    d. AUDIT, AUDIT-C or FAST scores (AUDIT categorised according to WHO guidelines on risk level: 0-7 – non-drinker or low-risk drinking; 8-15 – hazardous drinking; 16-19 – harmful drinking; 20-40 – possible dependence.[11]).

We have made all morbidity code lists used in this study available to download (https://doi.org/10.17037/data.00001071). We have also provided further detail regarding variable definitions in the Appendix (**Supplementary Text 1 and Supplementary Table 1**).

We considered the record of a score from an alcohol-screening test using a validated tool (AUDIT, AUDIT-C or FAST) as the gold standard for alcohol recording. The AUDIT score is a 10-item instrument with a maximum score of 40 designed specifically for screening for hazardous or harmful alcohol use in primary care.[11, 12] A score of eight or more on the AUDIT tool is associated with harmful or hazardous drinking, although a slightly lower threshold can be used in women. The AUDIT tool can be abbreviated in time-limited

For peer review only - http://bmjopen.bmj.com/site/about/guidelines.xhtml

settings to the first three questions (dealing with alcohol consumption only) (AUDIT-C) to give a score out of 12 or a four-item screening tool (FAST), which give a score out 16. These abbreviated tools have lower thresholds to indicate harmful or hazardous drinking than the standard AUDIT tool: AUDIT-C – three for women and four for men; and FAST – three.[13-15] Due to differences in scoring systems and different threshold scores to identify problem dinking between the AUDIT, AUDIT-C and FAST tools, we restricted our internal validation to those with the full AUDIT or AUDIT-C score.

For peer review only

Statistical analysis

Completeness of alcohol recording

To investigate the completeness of alcohol recording, we estimated the proportion of patients with a record for one or more morbidity codes relating to alcohol in the categories outlined above (i.e. i. Any code suggesting that alcohol was discussed in the consultation; ii. Codes indicating AUDIT, AUDIT-C or FAST screening; or iii. Codes quantifying alcohol use) in the five years prior to 1st January 2018 (each patient could have codes recorded in more than one category). We then described alcohol recording prevalence in strata of individual characteristics (i.e. age, sex, and other factors described above under the heading 'Factors associated with alcohol recording'). We also described the frequency of consultations with a record of an alcohol code in the five years before the study date, and the number of days from a patient's registration to their first record of an alcohol use code.

Internal validity

We validated codes quantifying the level of alcohol use (i.e. current/ex/non; light/moderate/heavy; units/week [Category iii above]) against an AUDIT score from the *same date* and the *same patient*. Full AUDIT versus AUDIT-C screening were considered separately. We calculated median AUDIT and AUDIT-C scores for each category of level of alcohol use recording: (a) drinking status, (b) drinking level and (c) units per week.

Similarly, we also validated codes indicating drinking status, drinking level and AUDIT/AUDIT-C scores against the number of units per week. For each variable, we only included a patient's most recent record (to the study date).

External validity

We identified current alcohol-use status for each individual with a relevant primary care alcohol record by identifying the most recent record indicating alcohol intake; i.e. either a morbidity code indicating alcohol-use status (classified as current or non-/ex-drinkers) or a record indicating the number of units of alcohol consumed per week. We described current alcohol-use status separately for men and women from CPRD, and compared the results to the Health Survey for England 2016.[16] Then, for CPRD patients with a recorded 'current' drinking status, we grouped the recorded number of units consumed per week into the following categories: men ≤14 (lower risk), 15-50 (increased risk), >50 (higher risk); women ≤14 (lower risk), 15-35 (increased risk), >35 (higher risk), and again compared these data to the Health Survey for England data 2016.[16]

Time trends in alcohol recording

To understand how alcohol recording has been changing over time, we carried out a cross sectional study on the 1st January each year between 2009 and 2018. We estimated the number of patients who would be eligible for the study, using the same criteria as above (registered for one year at a CPRD practice, and aged 18 or over on the study date), and calculated the proportion of patients with a record of alcohol consumption (based on a record of drinking status), and level of drinking (based on Read codes, units per week, AUDIT and AUDIT-C scores) over the past five years.

For peer review only - http://bmjopen.bmj.com/site/about/guidelines.xhtml

Sensitivity analyses

1. In the main analysis, we restricted the validation of alcohol codes to cases where patients had an AUDIT/AUDIT-C score recorded on the same date as another alcohol code of interest. In sensitivity analyses, we included cases where any AUDIT/AUDIT-C score was recorded up to 30 days before or after the code of interest.
2. In the main analysis, we recorded liver disease, hypertension and diabetes mellitus based on presence of a diagnosis at any time prior to the study date. We tested this definition by repeating the analysis limiting to diagnoses of these comorbidities within five years prior to the study date.
3. In the main analysis, we recorded depression and anxiety based on presence of a diagnosis within five years prior to the study date. We tested this definition by repeating the analysis limiting to diagnoses of these comorbidities within 12 months prior to the study date.

Data were analysed using Stata v14 (StataCorp, Texas, USA).

Patient involvement

Neither patients nor the public were involved in the design or analysis of the study. This work uses data provided by patients and collected by the UK National Health Service as part of their care and support.

For peer review only - http://bmjopen.bmj.com/site/about/guidelines.xhtml

Results

There were 1,768,651 adults aged 18 years or over, alive and registered, for at least one year, with a CPRD practice on 1st January 2018, and therefore included in the study; 49.4% were male, and their mean age was 49.7 years. The mean duration of registration prior to the study date was 18.7 years (standard deviation 14.5 years). One fifth had hypertension, 2.0% had liver disease, 6.8% had diabetes, 6.7% had depression and 4.6% had anxiety.

Prevalence

Overall, 918,254 (51.9%) patients had a record indicating that alcohol was discussed within the last five years. This included diagnoses of alcohol use disorders, their management, harm due to alcohol consumption, in addition to the consumption of alcohol.

Drinking status

Drinking status (current, ex-, non-drinker) was recorded in 862,330 (48.8%) of patients, though this was not consistent across patient groups. Recording of drinking status was higher in women compared to men (52.7% in women vs 44.7% in men), increased with age (30.6% in 18-24 years, 62.2% in 75+ years) and was more common among recently registered patients (in the last one to five years) than in those registered for five or more years (73.6% vs 43.0%, respectively). There were also differences in recording between geographic region (higher recording in the North of England, lower recording in Northern Ireland and Wales), by deprivation (recording was more common in deprived areas) and by ethnicity (highest for white patients). Patients who had missing smoking or BMI data had particularly low levels of alcohol status recorded, but even in those with smoking status recorded, drinking status was only recorded in approximately 50%-60% of patients. If smoking and BMI were recorded there were differences in the recording of alcohol status at different levels of smoking/BMI: for example, ex and current smokers were more likely to have alcohol status recorded than non-smokers, as were patients who were overweight.

For peer review only

Patients were also more likely to have alcohol status recorded with each of the morbidities investigated (liver disease, hypertension, diabetes, depression and anxiety). Patients with diabetes were the most likely to have alcohol levels recorded (82.3% had an alcohol record) (**Table 1**).

Level of consumption

Overall, 862,642 (48.8%) of adult patients had some level of alcohol consumption (including non-drinkers) recorded in the previous five years, identified through recording of Read codes (heavy, moderate, light or non-drinker), units per week or formal alcohol screening score using AUDIT, AUDIT-C or FAST.

Level of alcohol consumption data were recorded using Read coding for 31.6% of patients, in units per week for 24.4% of patients, and via a record of formal alcohol screening for 11.5% of patients with a Read code indicating that they had completed an AUDIT, AUDIT-C or FAST questionnaire, but only 8.5% of patients had the actual score available.

Recording of level of alcohol consumption by patient and practice characteristics are shown in **Table 1**. Patients with diabetes had the highest levels of alcohol consumption recording, but only 39.5% of them had a recording of units per week, and only 10.1% had a recorded AUDIT, AUDIT-C or FAST score.

Among patients with a recorded drinking status, 20.3% were non-drinkers, 3.7% ex-drinkers, and 76.1% current drinkers. **Table 2** describes levels of drinking amongst patients for whom it was recorded.

If we use all data (from all levels of alcohol consumption categories, i.e. coded heavy/moderate/light/non-drinker, recorded units per week or formal alcohol screening

For peer review only - http://bmjopen.bmj.com/site/about/guidelines.xhtml

score) to derive drinking status based on recorded status, units per week, and AUDIT scores, an additional 35,105 patients (2.0% of eligible patients) have a drinking status. This derived status shows that 20.8% are non-drinkers, 3.5% ex-drinkers and 75.7% are current drinkers.

Internal validity

In our study population, there were 77,212 (4.4%) patients with at least one AUDIT score record available, 21,099 (1.2%) patients with at least one AUDIT-C score record available, and 431,394 (24.4%) with units-per-week records. **Table 2** describes the cross validation between alcohol measures and AUDIT scores, AUDIT-C scores and units per week.

Drinking status: Current drinking (based on Read coding) was associated with a median AUDIT-C score of three (IQR 2-5), which is under the standard threshold defining risky drinking, and non/ex drinkers had a median AUDIT-C score of zero. For those with full AUDIT scores, the non and ex drinkers had median scores of zero, and current drinkers had a score of three (IQR 1-5). On average, current drinkers drank six units per week (IQR 2-14).

Drinking level: light and moderate drinkers had low AUDIT and AUDIT-C scores, while heavy drinkers had a median AUDIT-C score of 8 (IQR 6-9) and a median AUDIT score of 6 (IQR 5-9), which indicates alcohol education. Light drinkers drank 3 units per week (IQR 1-8), moderate drinkers 6 per week (IQR 2-14), and heavy drinkers 18 per week (IQR 9-30).

Units per week: increased with increasing AUDIT and AUDIT-C scores, but even the highest category of units per week (43+) only had a median AUDIT score of 11 (IQR 8-12), which indicates simple alcohol advice.

AUDIT scores: the majority (92.5%) of patients had scores in the lowest category (AUDIT score 0-7, where alcohol education is recommended according to WHO guidelines [11]). Patients with higher AUDIT scores consumed more units per week; for example, those in the lowest AUDIT score category (0-7,alcohol education) had a median units per week of four (IQR 2-10) compared to a median of 55 units per week (IQR 32-70) in the highest AUDIT

For peer review only - http://bmjopen.bmj.com/site/about/guidelines.xhtml

For peer review only

score category (score 20-40, indicating need for referral to specialist for diagnostic evaluation and treatment according to WHO guidelines [11]).

AUDIT-C scores: Patients with low risk AUDIT-C scores (<5) consumed fewer units per week; median 4 units per week (IQR 2-8) compared to a median of 12 units per week (IQR 6-20) in the high risk category (score ≥5). Where there were records for both AUDIT and AUDIT-C, in those with high risk AUDIT-C scores (which would indicate the need for a full AUDIT) the median AUDIT score was 6 (IQR 5-7).

For peer review only

External validity

For men and women, the proportions of current and non-drinkers identified using Read-coding in CPRD were close to those estimated in HSE (men: 80% current drinkers in CPRD, 83% in HSE; women: 73% current drinkers in CPRD, 78% in HSE). Units per week were also broadly comparable between CPRD and HSE although prevalence of higher risk drinking in CPRD looked slightly lower (**Table 3**). However, for estimates for both the proportion of current drinkers and units consumed per week were slightly lower in CPRD than HSE.

Time trends

Figure 1 illustrates time trends in recording of alcohol status and level of consumption. Between 2009 and 2018, there was an increase in recording in CPRD of both alcohol status and level of alcohol consumption from 19% to over 50%.

Frequency of recording

Among patients with at least one alcohol record in the last five years, 48.9% had just one record, 21.8% had two records, 10.8% had three records, and the remaining 48.5% had four or more records. 51.4% of patients with a record had their first alcohol record on the date of registration, 84.4% within the first month, and 95.5% within the first year of registration.

For peer review only - http://bmjopen.bmj.com/site/about/guidelines.xhtml

For patients with more than one record of alcohol consumption, the median duration between records was 339 days (IQR 175-452). This was shorter for patients with heavy consumption (237 days (IQR 70-432) compared with light drinkers (median 351 (195-459 days)) or non-drinkers (median 335 (179-438 days)).

For peer review only

For peer review only - http://bmjopen.bmj.com/site/about/guidelines.xhtml

Sensitivity analyses

1. Internal validity where we required that AUDIT score and units per week were recorded within 30 days of another code (rather than on the same date) resulted in more records being validated, but the results were identical to the main analysis (**Appendix Table 2**).

2. Individuals with diabetes mellitus, hypertension and liver disease diagnosed in the five years prior to the study date had higher alcohol recording than shown in the main analysis (where these variables included all patients ever diagnosed) (Diabetes 84.7% with recorded alcohol use in sensitivity analysis compared to 83.0% in main analysis with diabetes ever recorded; hypertension 75.8% with recorded alcohol use in sensitivity analysis, compared to 72.6% in main analysis; and liver disease 77.1% with recorded alcohol use in sensitivity analysis, compared to 69.9% in main analysis).

3. Similarly, depression and anxiety diagnosed in the past year had higher levels of alcohol recording than when diagnosed in the previous five years (depression: 62.4% vs 61.0% in main analysis; anxiety: 62.0% vs 60.9% in main analysis).

For peer review only

For peer review only - http://bmjopen.bmj.com/site/about/guidelines.xhtml

Discussion

Summary

In 2018, information about alcohol consumption was available for roughly half of all adult patients registered with practices contributing to the CPRD. Large differences in recording were seen across patient characteristics, in particular increasing with age, deprivation, and higher in patients with liver disease, hypertension, diabetes mellitus, depression and anxiety. Patients who were missing data on other risk factors such as smoking, BMI and ethnicity were also less likely to have any recording of alcohol use. Those with missing alcohol, smoking, BMI and ethnicity data may represent a group who have minimal contact with primary care. However, even among those with smoking recorded, GPs only recorded drinking status in approximately 50%-60% of patients. This suggests that GPs record alcohol use less frequently than smoking status; so even when there are opportunities to ask about health behaviours, alcohol use in not necessarily included. Alcohol was more likely to be recorded in those with higher levels of other health risks (smokers, obese) also suggesting that GPs may be more likely to ask about alcohol in patients who they perceive as at higher risk. These patients may also visit the GP more frequently, increasing the opportunities to ask about alcohol use.

The patterns of alcohol recording seen here show that UK GPs may be failing to record alcohol consumption at the most basic level of drinking status for half of their adult population. However, patients who are at greater risk from alcohol-related illness are being appropriately targeted (i.e. those with mental health conditions and diabetes, liver disease, hypertension) and have better recording. Importantly, 20-40% of these at-risk patients still lack data on alcohol consumption and the proportion of patients screened for alcohol consumption appropriately using a valid screening tool is even lower (although one explanation for this observations might be that individuals scoring at low risk are screened but this information is not recorded). Alcohol use should be discussed as part of the

For peer review only - http://bmjopen.bmj.com/site/about/guidelines.xhtml

cardiovascular disease health check for all patients over 40,[17] and suboptimal levels of alcohol recording are consistent with the low uptake of the health check recently described.[18] Importantly, just one third of 18-24 year olds had alcohol consumption recorded, and they are particularly likely to enagage in risky drinking behaviours.

Compared to a 2013 study using CPRD data (Khadjesari et al, 2013[19]), our study showed that while recording among new registrants has plateaued since 2009, remaining at around 75%, screening with AUDIT appears to have increased from 9% among new registrants in 2009 to 21.8% in our study. This may correspond with the introduction of new NICE guidance in 2010.[5] However, screening with a recommended screening tool for all registered adults remains at only 8%.

Our internal validation showed that, when there was a record of level of alcohol consumption, other measures of intake recorded in CPRD were likely to correspond. Recording of units per week corresponded to AUDIT scores (full AUDIT and AUDIT-C), and both measures corresponded to informal coding of alcohol consumption using Read codes (e.g. codes for light/moderate/heavy drinker) although the informal codes were less useful for discrimination between heavier drinkers (the median AUDIT score in those with a heavy drinker code was 6). AUDIT scores were used as the 'gold standard' because they are considered valid in comparison with other self-reported alcohol measures,[20, 21] but all self-reported measures of alcohol use are limited as they are likely to be under-reported.[22]

The Health Survey for England found that, in 2016, 17% of men and 22% of women were teetotal. Using CPRD drinking status, 20.5% of patients were recorded as non-drinkers, so this is comparable and demonstrates external validity, albeit also self-reported. The internal and external validity demonstrated in this study give weight to any study using alcohol data from CPRD. While care must be taken to avoid introducing bias by restricting to only patients with alcohol recorded, the measures of consumption are likely to be useful in

For peer review only - http://bmjopen.bmj.com/site/about/guidelines.xhtml

ranking participants in terms of their alcohol of consumption, with the caveats required by all self-reported alcohol research.

Strengths and Limitations

This study investigated alcohol recording across nearly 1.8 million UK adults, providing the power to explore recording in different subgroups of patients, and to understand in detail how alcohol use is recorded in primary care.

To validate, we used AUDIT scores recorded on the same date as the codes of interest. If an AUDIT questionnaire is completed, subsequent recording of alcohol consumption (in Read codes or units per week) might be more accurate than at a time when no scoring is done, and validity may be lower outside of these times. Additionally, GP practices that record both measures during the same consultation may be different to practices that only record one measure, and perhaps more likely to record alcohol use measures accurately. However, our sensitivity analyses using AUDIT scores recorded within 30 days before or after the date of the alcohol-related code of interest showed that records within a month of an AUDIT score or measure of units were internally consistent. For the external validation we used data from the health survey for England 2016, whereas the time frame for the prevalence estimates of alcohol use from CPRD were from 2013-2018 however although the time frames do not match perfectly 2016 is within the middle of the time frame and this seems an appropriate comparator.

Evidence suggests alcohol use in young people is common[23]. Indeed, the importance of screening and prevention in young people has been highlighted by recent NICE guidelines promoting alcohol intervention and education in the under 25s.[24] Unfortunately, we were unable to include 16-17 year olds in our analyses due to limited alcohol records in this age group and the resulting risk to anonymity. However, the finding of limited alcohol recording in

For peer review only - http://bmjopen.bmj.com/site/about/guidelines.xhtml

16-17 year olds is, in itself, an interesting insight into primary care alcohol recording in this vulnerable population.

Although we have demonstrated that alcohol recording and screening are higher for patients with certain high-risk conditions, it does not mean that GPs have acted upon the screening result, or that they have screened at an appropriate time. For example, patients with depression or anxiety may not have been screened at the time when depression was diagnosed. We showed that the majority of screening took place within one month of registration, so whether this is repeated during consultations for high-risk conditions is not clear from these data.

Between 2009 and 2018, the proportion of CPRD patients with alcohol recorded more than tripled from around 13% to nearly 49%. It is likely that this increase in primary-care alcohol recording was contributed to by the 2010 NICE guidelines recommending screening on registration with a GP, and studies demonstrating the efficacy and cost effectiveness of alcohol screening and brief interventions for alcohol misuse.[25] Screening is most likely to happen at the time of registration, shown here and in Khadjesari et al[19], and therefore the increase in recording in our study may be linked to patient turnover. The result is that patients who have been at a practice for a long time are much less likely to have any record of their alcohol consumption; while recording has improved, there are clear and important differences between patients with and without a record for alcohol use.

Implications for research

This study helps establish best practise in using primary care data to investigate alcohol use in research. Researchers using CPRD data should be aware of the limitations of alcohol data. Though the validity of the existing data has been described here, using only patients with complete alcohol use data may introduce selection bias into studies by preferentially including older, sicker, and more deprived patients.

For peer review only - http://bmjopen.bmj.com/site/about/guidelines.xhtml

Implications for practice

This study provides insight into how well aligned routine primary care practice is with NICE guidelines for alcohol screening. Recording of alcohol in primary care is not complete, meaning that opportunities for intervention may not be being identified. While we found evidence that alcohol use recording is higher in the high-risk groups highlighted by NICE (those with hypertension, depression), and at key times of patient registration recommended by NICE (among newly registered patients), there are still large numbers of patients in whom we might expect risky drinking who are not having their alcohol use recorded, and who therefore may not be targeted for intervention. In particular, younger individuals, in whom alcohol is a major cause of morbidity, maybe being overlooked.

For peer review only

For peer review only - http://bmjopen.bmj.com/site/about/guidelines.xhtml

References

1. NHS Digital, *Health Survey for England, 2017: Multiple risk factors, Table 13.* 2018.
2. Home Office, *A minimum unit price for alcohol. Impact Assessment.* 2012.
3. GBD 2016 Alcohol Collaborators, *Alcohol use and burden for 195 countries and territories, 1990-2016: a systematic analysis for the Global Burden of Disease Study 2016.* Lancet, 2018. **392**(10152): p. 1015-1035.
4. Babor, T.F. and J.C. Higgins-Biddle, *BRIEF INTERVENTION For Hazardous and Harmful Drinking: A Manual for Use in Primary Care.* 2001.
5. NICE, *Alcohol-use disorders: prevention. NICE guidelines PH24.* 2010.
6. Public Health England. *NHS Health Check: Best practice guidance*. 2017; Available from: http://www.healthcheck.nhs.uk/document.php?o=1308.
7. Stewart, D., et al., *Alcohol consumption and all-cause mortality: an analysis of general practice database records for patients with long-term conditions.* J Epidemiol Community Health, 2017. **71**(8): p. 729-735.
8. Otete, H.E., et al., *Sex and age differences in the early identification and treatment of alcohol use: a population-based study of patients with alcoholic cirrhosis.* Addiction, 2015. **110**(12): p. 1932-40.
9. Mathur, R., et al., *Completeness and usability of ethnicity data in UK-based primary care and hospital databases.* J Public Health, 2014. **36**(4): p. 684-92.
10. Bhaskaran, K., et al., *Representativeness and optimal use of body mass index (BMI) in the UK Clinical Practice Research Datalink (CPRD).* BMJ Open, 2013. **3**(9): p. e003389.
11. Babor, T., et al., *The Alcohol Use Disorders Identification Test: Guidelines for Use in Primary Care. Available from http://apps.who.int/iris/bitstream/handle/10665/67205/WHO_MSD_MSB_01.6a.pdf?sequence=1*. 2001.
12. Saunders, J.B., et al., *Development of the Alcohol Use Disorders Identification Test (AUDIT): WHO Collaborative Project on Early Detection of Persons with Harmful Alcohol Consumption--II.* Addiction, 1993. **88**(6): p. 791-804.
13. Reinert, D.F. and J.P. Allen, *The alcohol use disorders identification test: an update of research findings.* Alcohol Clin Exp Res, 2007. **31**(2): p. 185-99.
14. Hodgson, R., et al., *The FAST Alcohol Screening Test.* Alcohol Alcohol, 2002. **37**(1): p. 61-6.
15. Hodgson, R.J., et al., *Fast screening for alcohol misuse.* Addict Behav, 2003. **28**(8): p. 1453-63.
16. NHS Digital, *Health Survey for England, 2016.* 2016.
17. Public Health England, *NHS Health Check: Best practice guidance. 2017; Available from: http://www.healthcheck.nhs.uk/document.php?o=1308*. 2017.
18. Coghill, N., et al., *NHS health checks: a cross- sectional observational study on equity of uptake and outcomes.* BMC Health Serv Res, 2018. **18**(1): p. 238.
19. Khadjesari, Z., et al., *Alcohol consumption screening of newly-registered patients in primary care: a cross-sectional analysis.* Br J Gen Pract, 2013. **63**(615): p. e706-12.
20. Allen, J.P., et al., *A review of research on the Alcohol Use Disorders Identification Test (AUDIT).* Alcohol Clin Exp Res, 1997. **21**(4): p. 613-9.
21. Reinert, D.F. and J.P. Allen, *The Alcohol Use Disorders Identification Test (AUDIT): A Review of Recent Research.* Alcoholism: Clinical and Experimental Research, 2002. **26**(2): p. 272-279.
22. Feunekes, G.I., et al., *Alcohol intake assessment: the sober facts.* Am J Epidemiol, 1999. **150**(1): p. 105-12.
23. NHS Digital, *Smoking, Drinking and Drug Use Among Young People in England - 2016*. 2017.
24. NICE, *Alcohol interventions in secondary and further education. NG135. Published 12 August 2019.* 2019.

For peer review only - http://bmjopen.bmj.com/site/about/guidelines.xhtml

25. Angus, C., et al., *What are the Implications for Policy Makers? A Systematic Review of the Cost-Effectiveness of Screening and Brief Interventions for Alcohol Misuse in Primary Care.* Front Psychiatry, 2014. **5**: p. 114.

For peer review only

Author statement

EC and KM contributed equally. EH, SC, KM, LS, JQ, EC and RD contributed to the design of the study. EH and EC extracted the data, EC, KM and EH wrote the statistical programmes, and wrote the first draft. All authors (EH, SC, KM, EC, LS, JQ, RD) contributed to further drafts and approved the final manuscript.

Conflicts of interest

None.

Funding

This research received no specific grant from any funding agency in the public, commercial or not-for-profit sectors. EH holds a NIHR postdoctoral fellowship (grant number PDF-2016-09-029).

Role of the funding source

The NIHR had no role in the design, analysis or writing up of this study.

Patient and public involvement

We did not involve patients or the public in our work.

Data access

EC and EH had full access to the CPRD database in order to create the study population.

Data sharing statement

We obtained data from the Clinical Practice Research Datalink (CPRD). CPRD is a research service that provides primary care and linked data for public health research. CPRD data governance, and our own license to use CPRD data, do not allow us to distribute or make available patient data directly to other parties. Researchers may apply for data access at www.cprd.com, and must have their study protocol approved by the Independent Scientific Advisory Committee for MHRA database research (details at www.cprd.com/isac).

For peer review only

For peer review only - http://bmjopen.bmj.com/site/about/guidelines.xhtml

Ethical approval

This study was approved by the London School of Hygiene and Tropical Medicine Ethics Committee (Approval Number 14454).

For peer review only

For peer review only - http://bmjopen.bmj.com/site/about/guidelines.xhtml

Tables

For peer review only

For peer review only - http://bmjopen.bmj.com/site/about/guidelines.xhtml

Table 1 Characteristics of adult patients registered in the CPRD, on 1st January 2018, and those with records indicating drinking status, level of consumption, units per week and AUDIT/FAST scores.

	Study population: All individuals 18+yrs registered with a CPRD practice on 1st January 2018*		Individuals in the study population with recording of alcohol use in different categories in the 5 years before 1st January 2018**															
			Has a record for any Read code relating to alcohol use		Has a record for a Read code indicating alcohol use, from which alcohol status could be derived[a]		Has a record of any drinking status recorded as Read code, or captured through structured data areas in GP software[b]		Has level of consumption recorded in Read codes[c]		Has units per week recorded		Has had AUDIT[d] or FAST screen		Has AUDIT[d] or FAST score recorded		Has any level of drinking recorded in Read, units or score[e]	
	N (col %)*		n (row %)**		n (row %)**		n (row %)**		n (row %)**		n (row %)**		n (row %)**		n (row %)**		n (row %)**	
**N**	1,768,651	(100)	918,254	(51.9)	897,535	(50.7)	862,330	(48.8)	558,747	(31.6)	431,394	(24.4)	204,182	(11.5)	150,457	(8.5)	862,642	(48.8)
**Sex**																		
Male	872,865	(49.4)	416,611	(47.7)	406,508	(46.6)	389,946	(44.7)	231,204	(26.5)	222,678	(25.5)	93,900	(10.8)	69,661	(8)	389,771	(44.7)
Female	895,786	(50.6)	501,643	(56)	491,027	(54.8)	472,384	(52.7)	327,543	(36.6)	208,716	(23.3)	110,282	(12.3)	80,796	(9)	472,871	(52.8)
**Age group**																		
18-24	168,796	(9.5)	58,379	(34.6)	54,830	(32.5)	51,584	(30.6)	36,755	(21.8)	16,537	(9.8)	15,629	(9.3)	8,564	(5.1)	51,647	(30.6)
25-34	285,015	(16.1)	131,905	(46.3)	128,051	(44.9)	119,772	(42)	76,097	(26.7)	50,686	(17.8)	34,313	(12)	25,222	(8.8)	120,830	(42.4)
35-44	300,819	(17)	136,265	(45.3)	133,289	(44.3)	125,865	(41.8)	77,277	(25.7)	60,492	(20.1)	34,273	(11.4)	26,405	(8.8)	126,761	(42.1)
45-54	333,132	(18.8)	171,021	(51.3)	167,309	(50.2)	160,742	(48.3)	96,024	(28.8)	88,593	(26.6)	37,693	(11.3)	28,709	(8.6)	160,636	(48.2)
55-64	277,217	(15.7)	160,137	(57.8)	157,180	(56.7)	152,786	(55.1)	94,656	(34.1)	86,476	(31.2)	32,478	(11.7)	24,618	(8.9)	151,987	(54.8)
65-74	223,120	(12.6)	145,105	(65)	142,776	(64)	139,320	(62.4)	92,173	(41.3)	77,896	(34.9)	29,967	(13.4)	22,483	(10.1)	139,043	(62.3)
75+	180,552	(10.2)	115,442	(63.9)	114,100	(63.2)	112,261	(62.2)	85,765	(47.5)	50,714	(28.1)	19,829	(11)	14,456	(8)	111,738	(61.9)
**Time since registration**																		
1-5 years	334,629	(18.9)	274,706	(82.1)	266,784	(79.7)	246,126	(73.6)	150,578	(45)	114,320	(34.2)	102,332	(30.6)	76,254	(22.8)	253,079	(75.6)
>=5 years	1,434,022	(81.1)	643,548	(44.9)	630,751	(44)	616,204	(43)	408,169	(28.5)	317,074	(22.1)	101,850	(7.1)	74,203	(5.2)	609,563	(42.5)
**Region**																		
North England	231,297	(13.1)	138,506	(59.9)	136,272	(58.9)	128,522	(55.6)	76,323	(33)	76,396	(33)	45,957	(19.9)	40,689	(17.6)	132,999	(57.5)
South England	607,016	(34.3)	320,297	(52.8)	308,052	(50.7)	285,002	(47)	174,695	(28.8)	143,027	(23.6)	106,862	(17.6)	77,866	(12.8)	295,640	(48.7)
Northern Ireland	114,311	(6.5)	55,838	(48.8)	53,203	(46.5)	52,981	(46.3)	34,830	(30.5)	22,297	(19.5)	13,363	(11.7)	1,104	(1)	50,621	(44.3)
Scotland	397,133	(22.5)	207,247	(52.2)	205,159	(51.7)	201,171	(50.7)	138,776	(34.9)	103,816	(26.1)	35,951	(9.1)	30,202	(7.6)	197,790	(49.8)
Wales	418,894	(23.7)	196,366	(46.9)	194,849	(46.5)	194,654	(46.5)	134,123	(32)	85,858	(20.5)	2,049	(0.5)	596	(0.1)	185,592	(44.3)
**Deprivation**																		
1 (least deprived)	190,262	(10.8)	102,692	(54)	99,633	(52.4)	90,969	(47.8)	44,305	(23.3)	57,838	(30.4)	34,487	(18.1)	27,071	(14.2)	95,812	(50.4)
2	134,191	(7.6)	74,536	(55.5)	72,437	(54)	66,664	(49.7)	35,727	(26.6)	39,943	(29.8)	25,722	(19.2)	20,614	(15.4)	69,844	(52)
3	118,777	(6.7)	67,455	(56.8)	65,713	(55.3)	61,953	(52.2)	36,121	(30.4)	33,892	(28.5)	20,030	(16.9)	15,365	(12.9)	63,199	(53.2)
4	108,991	(6.2)	62,745	(57.6)	61,689	(56.6)	58,234	(53.4)	39,674	(36.4)	26,879	(24.7)	19,040	(17.5)	15,816	(14.5)	59,403	(54.5)
5 (most deprived)	96,301	(5.4)	57,840	(60.1)	56,804	(59)	51,770	(53.8)	38,342	(39.8)	21,983	(22.8)	21,497	(22.3)	18,212	(18.9)	54,968	(57.1)
Missing	1,120,129	(63.3)	552,986	(49.4)	541,259	(48.3)	532,740	(47.6)	364,578	(32.5)	250,859	(22.4)	83,406	(7.4)	53,379	(4.8)	519,416	(46.4)

CONTINUED…

For peer review only - http://bmjopen.bmj.com/site/about/guidelines.xhtml

	Study population: All individuals 18+yrs registered with a CPRD practice on 1st January 2018* N (col %)*	Individuals in the study population with recording of alcohol use in different categories in the 5 years before 1st January 2018** Has a record for any Read code relating to alcohol use n (row %)**	Has a record for a Read code indicating alcohol use, from which alcohol status could be derived[a] n (row %)**	Has a record of any drinking status recorded as Read code, or captured through structured data areas in GP software[b] n (row %)**	Has level of consumption recorded in Read codes[c] n (row %)**	Has units per week recorded n (row %)**	Has had AUDIT[d] or FAST screen n (row %)**	Has AUDIT[d] or FAST score recorded n (row %)**	Has any level of drinking recorded in Read, units or score[e] n (row %)**
**Ethnicity**									
White	807,709 (45.7)	502,125 (62.2)	489,808 (60.6)	465,333 (57.6)	287,175 (35.6)	254,409 (31.5)	135,229 (16.7)	104,643 (13)	470,554 (58.3)
South Asian	46,593 (2.6)	28,089 (60.3)	27,111 (58.2)	25,603 (55)	21,297 (45.7)	5,874 (12.6)	9,611 (20.6)	6,835 (14.7)	26,306 (56.5)
Other	60,520 (3.4)	38,086 (62.9)	36,482 (60.3)	33,898 (56)	26,559 (43.9)	9,974 (16.5)	13,896 (23)	10,120 (16.7)	35,212 (58.2)
Missing	853,829 (48.3)	349,954 (41)	344,134 (40.3)	337,496 (39.5)	223,716 (26.2)	161,137 (18.9)	45,446 (5.3)	28,859 (3.4)	330,570 (38.7)
**Smoking status**									
Non smoker	746,074 (42.2)	383,302 (51.4)	373,622 (50.1)	356,424 (47.8)	233,122 (31.2)	163,733 (21.9)	91,386 (12.2)	66,152 (8.9)	359,039 (48.1)
Current smoker	254,850 (14.4)	147,616 (57.9)	144,275 (56.6)	139,028 (54.6)	90,505 (35.5)	68,758 (27)	31,617 (12.4)	23,328 (9.2)	137,108 (53.8)
Ex-smoker	565,583 (32)	347,945 (61.5)	342,126 (60.5)	332,036 (58.7)	213,908 (37.8)	181,109 (32)	71,773 (12.7)	53,832 (9.5)	331,127 (58.5)
Missing	202,144 (11.4)	39,391 (19.5)	37,512 (18.6)	34,842 (17.2)	21,212 (10.5)	17,794 (8.8)	9,406 (4.7)	7,145 (3.5)	35,368 (17.5)
**BMI**									
Normal (18·5–25 kg/m²)	533,130 (30.1)	298,415 (56)	290,354 (54.5)	277,070 (52)	172,796 (32.4)	139,540 (26.2)	74,169 (13.9)	54,609 (10.2)	278,618 (52.3)
Underweight (BMI <18·5 kg/m²)	32,421 (1.8)	16,915 (52.2)	16,262 (50.2)	15,481 (47.7)	10,904 (33.6)	6,055 (18.7)	4,327 (13.3)	2,938 (9.1)	15,570 (48)
Overweight/obese (BMI ≥25 kg/m²)	865,940 (49)	543,118 (62.7)	534,282 (61.7)	519,336 (60)	346,490 (40)	265,441 (30.7)	110,880 (12.8)	82,856 (9.6)	516,657 (59.7)
Missing	337,160 (19.1)	59,806 (17.7)	56,637 (16.8)	50,443 (15)	28,557 (8.5)	20,358 (6)	14,806 (4.4)	10,054 (3)	51,797 (15.4)
**Liver disease**	35,542 (2)	25,404 (71.5)	24,854 (69.9)	24,340 (68.5)	16,978 (47.8)	12,847 (36.1)	5,012 (14.1)	3,684 (10.4)	24,069 (67.7)
**Hypertension**	358,285 (20.3)	262,785 (73.3)	260,050 (72.6)	256,762 (71.7)	183,616 (51.2)	134,479 (37.5)	44,646 (12.5)	33,173 (9.3)	254,136 (70.9)
**Diabetes**	120,744 (6.8)	100,787 (83.5)	100,158 (83)	99,378 (82.3)	79,211 (65.6)	47,668 (39.5)	16,145 (13.4)	12,164 (10.1)	98,451 (81.5)
**Depression**	118,644 (6.7)	73,868 (62.3)	72,314 (61)	69,961 (59)	47,499 (40)	33,663 (28.4)	15,581 (13.1)	11,185 (9.4)	69,083 (58.2)
**Anxiety**	80,658 (4.6)	50,048 (62)	49,111 (60.9)	47,593 (59)	31,840 (39.5)	23,330 (28.9)	10,158 (12.6)	7,348 (9.1)	47,006 (58.3)

a. Alcohol status: Current drinker, ex-drinker, non-drinker from Read codes.
b. Alcohol status: Current drinker, ex-drinker, non-drinker from Read codes and captured through structured data areas in GP software.
c. Level of drinking: none, light, moderate, heavy drinker
d. Includes full AUDIT and short-form AUDIT-C screening.
e. Level of drinking derived from: Read codes, units per week or AUDIT/AUDIT-C/FAST screening score

*For the column (shaded) summarising characteristics for the whole study population (i.e. all adults registered in CPRD on 1st January 2018) – percentages are out of the whole study population (i.e. column %s).
**For subsequent columns (unshaded) – which cover individuals identified through different types of alcohol-use recording – percentages represent the number of individuals recorded with the specific category of coding as a percentage of the study population in that particular stratum; That is, row percentage with denominator from study population in that stratum (e.g. for all women in the study population [n=895,786], 56% [n=501,643] have a record of any Read code relating to alcohol use).

Table 2 Internal validation comparing measures of alcohol use in CPRD to AUDIT and AUDIT-C scores, and to units per week. N=1,768,651

For peer review only - http://bmjopen.bmj.com/site/about/guidelines.xhtml

	N	(%)	N patients with AUDIT score on the same date as record		AUDIT score		N patients with AUDIT C score on the same date as record		AUDIT-C score		N patients with units per week recorded on same date as record		Units per week	
			n	(%)	median	(IQR)	n	(%)	median	(IQR)	n	(%)	median	(IQR)
**Current drinking status***														
Non	174,970	(20.3)	2571	(1.5)	0	(0-0)	1022	(0.6)	0	(0-0)	905	(0.5)	0	(0-1)
Ex	31,526	(3.7)	292	(0.9)	0	(0-0)	39	(0.1)	0	(0-0)	1,004	(3.2)	0	(0-1)
Current	655,834	(76.1)	21563	(3.3)	3	(1-5)	4283	(0.7)	3	(2-5)	333,374	(50.8)	6	(2-14)
**Current drinking level (Read codes)**														
Non-drinker	222835	(39.9)	2880	(1.3)	0	(0-0)	1061	(0.5)	0	(0-0)	2,278	(1)	0	(0-2)
Light drinker	255,919	(45.8)	2,962	(1.2)	2	(1-3)	1,058	(0.4)	2	(1-3)	19,063	(7.4)	3	(1-8)
Moderate drinker	52,070	(9.3)	1,122	(2.2)	0	(0-4)	225	(0.4)	3	(2-4)	12,753	(24.5)	6	(2-14)
Heavy drinker	27,923	(5)	2,860	(10.2)	6	(5-9)	125	(0.4)	8	(6-9)	10,319	(36.9)	18	(9-30)
**Units per week**														
0	38,929	(9)	1,830	(4.7)	0	(0-0)	123	(0.3)	0	(0-1)				
1-14	298,441	(69.2)	10,905	(3.7)	3	(2-4)	1,982	(0.7)	3	(2-4)	n/a		n/a	
15-42	80,449	(18.6)	2,761	(3.4)	6	(4-8)	364	(0.5)	6	(4-7)				
43+	13,575	(3.1)	222	(1.6)	11	(8-12)	17	(0.1)	8	(6-10)				
**AUDIT C score category**														
0-4; Low risk	16,206	(76.8)	3,994	(24.6)	2	(0-3)	n/a		n/a		1,781	(11)	4	(2-8)
5-12; High risk	4,893	(23.2)	1,129	(23.1)	6	(5-7)					702	(14.3)	12	(6-20)
**AUDIT score category (and corresponding WHO recommended intervention[11])**														
0-7; Alcohol Education	71,401	(92.5)					4,881	(6.8)	2	(1-4)	14,156	(19.8)	4	(2-10)
8-15; Simple Advice	5,405	(7)					242	(4.5)	9	(8-10)	1,322	(24.9)	20	(14-30)
16-19; Simple Advice plus Brief Counseling and Continued Monitoring	206	(0.3)	n/a		n/a		0	(0)		(-)	45	(21.8)	35	(20-50)
20-40; Referral to Specialist for Diagnostic Evaluation and Treatment	200	(0.3)					0	(0)		(-)	22	(11.2)	55	(32-70)

*Current drinking status based on recorded status in Read codes and captured through structured data areas in GP software (not including derived status based on units consumed or AUDIT score).

For peer review only - http://bmjopen.bmj.com/site/about/guidelines.xhtml

Table 3 External validation, comparing most recent record of alcohol status in CPRD to data from the Health Survey for England 2016.

	CPRD		HSE
	**n**	**%**	**%**
**Men**			
**Non drinker***	76,544	20	17
**Current drinker**	313,402	80	83
**Units per week among current drinkers with units per week recorded:**			
≤14 (lower risk)	*122,787*	*67*	*63*
15-50 (increased risk)	*53,923*	*30*	*31*
>50 (higher risk)	*5,987*	*3*	*6*
**Women**			
**Non drinker***	129,952	28	22
**Current drinker**	342,432	73	78
**Units per week among current drinkers with units per week recorded:**			
≤14 (lower risk)	*146,177*	*88*	*78*
15-35 (increased risk)	*16,434*	*10*	*16*
>35 (higher risk)	*3,094*	*2*	*5*

*Includes patients labelled non- and ex-drinker

For peer review only

Figure captions

Figure 1. Recording of alcohol status and time trends between 2009 and 2018.

For peer review only

For peer review only - http://bmjopen.bmj.com/site/about/guidelines.xhtml

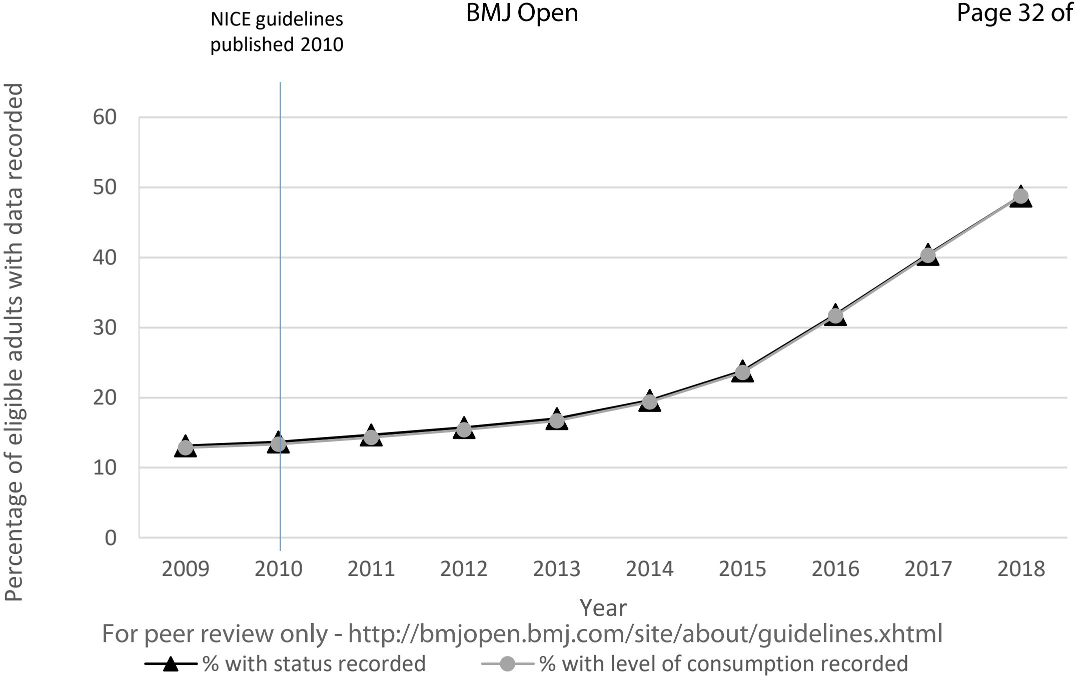

For peer review only - http://bmjopen.bmj.com/site/about/guidelines.xhtml

Appendices

Supplementary Text 1. Detailed alcohol-use definitions

i. Any code suggesting that alcohol was discussed in the consultation (**Supplementary Table 1**, Column 1).

ii. Codes indicating AUDIT or FAST screening (**Supplementary Table 1**, Columns 4 and 5)

iii. Codes quantifying alcohol use, comprised of:

   a. Drinking status (categorised as current drinker, ex drinker, non drinker; recorded in:
      - Read codes (**Supplementary Table 1**, Column 2)
      - Structured data, using data from the CPRD Additional Clinical Details file recorded under Entity Type 5, data field 1 (status) (Entity Type is the identifier that represents the structured data area in Vision where the data was entered)

   b. Level of drinking (non, light, moderate, heavy drinker; as recorded in Read codes (**Supplementary Table 1**, Column 3))

   c. Alcohol units consumed per week, using an existing (CALIBER: https://www.ucl.ac.uk/health-informatics/caliber) variable definition for 'number of alcohol unit consumption per week recorded in primary care (CPRD)',[1] using data from the CPRD Additional Clinical Details file recorded under Entity Type 5, data field 2 (units per week). We categorised units per week as: "None", "1-14, "15-42" or "43+" units per week. 14 units per week is the recommended limit for men and women according to current UK government guidelines.[2]

   d. AUDIT or FAST scores (AUDIT categorised according to WHO guidelines on risk level: 1-7 - low risk drinking; 8-15 - hazardous drinking; 16-19 - harmful drinking; 20-40 – possible dependence.[3]). Scores resulting from AUDIT/FAST screening (i.e. associated with a Read morbidity code indicating use of AUDIT/FAST), were obtained from the Additional Clinical Details file under Entity Type 172.

For peer review only - http://bmjopen.bmj.com/site/about/guidelines.xhtml

Supplementary Table 1. Read codes for alcohol data in CPRD (code lists also available for download: https://doi.org/10.17037/data.00001071).

CPRD medcode	Read code	Clinical term	1 Any alcohol-related code	2 Current drinking status	3 Codes indicating level of alcohol use	4 AUDIT screening	5 FAST screening
47555	90.95	cerebral degeneration due to alcoholism	1	0	0	0	0
27	136..00	alcohol consumption	1	0	0	0	0
12949	1361.00	Teetotaller	1	non	non	0	0
12970	1361.11	non drinker alcohol	1	non	non	0	0
4447	1361.12	non-drinker alcohol	1	non	non	0	0
12975	1362.00	trivial drinker - <1u/day	1	curr	L	0	0
385	1362.11	drinks rarely	1	curr	L	0	0
749	1362.12	drinks occasionally	1	curr	L	0	0
12972	1363.00	light drinker - 1-2u/day	1	curr	L	0	0
322	1364.00	moderate drinker - 3-6u/day	1	curr	M	0	0
1618	1365.00	heavy drinker - 7-9u/day	1	curr	H	0	0
12977	1366.00	very heavy drinker - >9u/day	1	curr	H	0	0
967	1367.00	stopped drinking alcohol	1	ex	non	0	0
12978	1368.00	alcohol consumption unknown	1	0	0	0	0
12976	1369.00	suspect alcohol abuse - denied	1	0	0	0	0
22933	136A.00	ex-trivial drinker (<1u/day)	1	0	0	0	0
102665	136a.00	increasing risk drinking	1	curr	H	0	0
26471	136B.00	ex-light drinker - (1-2u/day)	1	0	0	0	0
99877	136b.00	feels should cut down drinking	1	curr	H	0	0
19495	136C.00	ex-moderate drinker - (3-6u/d)	1	0	0	0	0
102448	136c.00	higher risk drinking	1	curr	H	0	0
19493	136D.00	ex-heavy drinker - (7-9u/day)	1	0	0	0	0
103230	136d.00	lower risk drinking	1	curr	M	0	0
12983	136E.00	ex-very heavy drinker-(>9u/d)	1	0	0	0	0
106560	136e.00	declines to state current alcohol consumption	1	0	0	0	0
12971	136F.00	spirit drinker	1	curr	0	0	0
2689	136G.00	beer drinker	1	curr	0	0	0
12968	136H.00	drinks beer and spirits	1	curr	0	0	0
12969	136I.00	drinks wine	1	curr	0	0	0
956	136J.00	social drinker	1	curr	M	0	0

For peer review only

CPRD medcode	Read code	Clinical term	1 Any alcohol-related code	2 Current drinking status	3 Codes indicating level of alcohol use	4 AUDIT screening	5 FAST screening
12982	136K.00	alcohol intake above recommended sensible limits	1	curr	H	0	0
26472	136L.00	alcohol intake within recommended sensible limits	1	curr	M	0	0
12979	136M.00	current non drinker	1	non	non	0	0
12980	136N.00	light drinker	1	curr	L	0	0
12985	136O.00	moderate drinker	1	curr	M	0	0
8999	136P.00	heavy drinker	1	curr	H	0	0
12984	136Q.00	very heavy drinker	1	curr	H	0	0
19401	136R.00	binge drinker	1	curr	H	0	0
19494	136S.00	hazardous alcohol use	1	curr	H	0	0
30695	136T.00	harmful alcohol use	1	curr	H	0	0
93415	136V.00	alcohol units per week	1	0	0	0	0
94670	136W.00	alcohol misuse	1	curr	H	0	0
97126	136X.00	alcohol units consumed on heaviest drinking day	1	0	0	0	0
101718	136Y.00	drinks in morning to get rid of hangover	1	curr	0	0	0
12981	136Z.00	alcohol consumption nos	1	0	0	0	0
18156	13Y8.00	alcoholics anonymous	1	0	0	0	0
84218	13ZY.00	disqualified from driving due to excess alcohol	1	0	0	0	0
8430	1462.00	h/o: alcoholism	1	0	0	0	0
38061	1B1c.00	alcohol induced hallucinations	1	curr	0	0	0
44783	1D19.00	pain in lymph nodes after alcohol consumption	1	0	0	0	0
24735	2577.00	o/e - breath - alcohol smell	1	curr	0	0	0
10161	2577.11	o/e - alcoholic breath	1	curr	0	0	0
69691	388j.00	cage questionnaire	1	0	0	0	0
70161	388j.00	cage questionnaire	1	0	0	0	0
93624	388u.00	fast alcohol screening test	1	0	0	0	1
96107	38D2.00	single alcohol screening questionnaire	1	0	0	0	0
94838	38D3.00	alcohol use disorders identification test	1	0	0	1	0
95744	38D4.00	alcohol use disorder identificatn test consumptn questionnre	1	0	0	1	0
97501	38D5.00	alcoh use disor id test piccinelli consumption questionnaire	1	0	0	1	0
100493	38Df.00	five-shot questionnaire on heavy drinking	1	0	0	0	0
102577	38Dz.00	severity of alcohol dependence questionnaire	1	0	0	0	0
102770	38Dz.11	sadq - severity of alcohol dependence questionnaire	1	0	0	0	0

For peer review only

CPRD medcode	Read code	Clinical term	1 Any alcohol-related code	2 Current drinking status	3 Codes indicating level of alcohol use	4 AUDIT screening	5 FAST screening
100989	4I91.11	breath alcohol level	1	0	0	0	0
17266	63C7.00	maternal alcohol abuse	1	0	0	0	0
12442	66e..00	alcohol disorder monitoring	1	curr	0	0	0
32964	66e0.00	alcohol abuse monitoring	1	0	0	0	0
11491	6792.00	health ed. - alcohol	1	0	0	0	0
35859	67A5.00	pregnancy alcohol advice	1	0	0	0	0
18711	67H0.00	lifestyle advice regarding alcohol	1	0	0	0	0
105144	67K6.00	cycle of change stage, alcohol	1	0	0	0	0
19489	6892.00	alcohol consumption screen	1	0	0	0	0
9264	68S..00	alcohol consumption screen	1	0	0	0	0
56410	7P22100	delivery of rehabilitation for alcohol addiction	1	0	0	0	0
2083	8BA8.00	alcohol detoxification	1	0	0	0	0
7692	8CAM.00	patient advised about alcohol	1	0	0	0	0
102564	8CAM000	advised to abstain from alcohol consumption	1	0	0	0	0
97309	8CAv.00	advised to contact primary care alcohol worker	1	curr	0	0	0
37264	8CE1.00	alcohol leaflet given	1	0	0	0	0
29691	8G32.00	aversion therapy - alcoholism	1	0	0	0	0
21650	8H35.00	admitted to alcohol detoxification centre	1	0	0	0	0
9849	8H7p.00	referral to community alcohol team	1	0	0	0	0
12554	8HHe.00	referral to community drug and alcohol team	1	0	0	0	0
94553	8HkG.00	referral to specialist alcohol treatment service	1	0	0	0	0
96993	8HkJ.00	referral to alcohol brief intervention service	1	0	0	0	0
94669	8IA7.00	alcohol consumption screening test declined	1	0	0	0	0
97261	8IAF.00	brief intervention for excessive alcohol consumptn declined	1	curr	0	0	0
97680	8IAJ.00	declined referral to specialist alcohol treatment service	1	curr	0	0	0
102247	8IAt.00	extended interven for excessive alcohol consumption declined	1	curr	0	0	0
103459	8IEA.00	referral to community alcohol team declined	1	curr	0	0	0
43813	9EQ..11	police:venesect-alcohol	1	0	0	0	0
102121	9EQ..12	police:venesect-alcohol	1	0	0	0	0
11740	9k1..00	alcohol misuse - enhanced services administration	1	curr	0	0	0
35330	9k11.00	alcohol consumption counselling	1	curr	0	0	0
63529	9k12.00	alcohol misuse - enhanced service completed	1	0	0	0	0

For peer review only - http://bmjopen.bmj.com/site/about/guidelines.xhtml

For peer review only

CPRD medcode	Read code	Clinical term	1 Any alcohol-related code	2 Current drinking status	3 Codes indicating level of alcohol use	4 AUDIT screening	5 FAST screening
48545	9k13.00	alcohol questionnaire completed	1	0	0	0	0
47123	9k14.00	alcohol counselling by other agencies	1	0	0	0	0
90714	9k15.00	alcohol screen - audit completed	1	0	0	1	0
94963	9k16.00	alcohol screen - fast alcohol screening test completed	1	0	0	0	1
95663	9k17.00	alcohol screen - audit c completed	1	0	0	1	0
94485	9k18.00	alcohol screen - audit pc completed	1	0	0	1	0
95944	9k19.00	alcohol assesment declined - enhanced services admin	1	0	0	0	0
96259	9k19.11	alcohol assessment declined	1	0	0	0	0
96053	9k1A.00	brief intervention for excessive alcohol consumptn completed	1	0	0	0	0
96054	9k1B.00	extended intervention for excessive alcohol consumptn complt	1	0	0	0	0
9489	9NN2.00	under care of community alcohol team	1	0	0	0	0
65754	C150500	alcohol-induced pseudo-cushing's syndrome	1	0	0	0	0
16237	E01..00	alcoholic psychoses	1	0	0	0	0
16225	E010.00	alcohol withdrawal delirium	1	curr	0	0	0
22277	E010.11	dts - delirium tremens	1	curr	0	0	0
1476	E010.12	Delirium tremens	1	curr	0	0	0
20762	E011.00	alcohol amnestic syndrome	1	0	0	0	0
4500	E011000	korsakov's alcoholic psychosis	1	0	0	0	0
11106	E011100	korsakov's alcoholic psychosis with peripheral neuritis	1	0	0	0	0
18636	E011200	wernicke-korsakov syndrome	1	0	0	0	0
41920	E011z00	alcohol amnestic syndrome nos	1	0	0	0	0
54505	E012.00	other alcoholic dementia	1	0	0	0	0
27342	E012.11	alcoholic dementia nos	1	0	0	0	0
37946	E012000	chronic alcoholic brain syndrome	1	0	0	0	0
25110	E013.00	alcohol withdrawal hallucinosis	1	curr	0	0	0
57939	E014.00	pathological alcohol intoxication	1	curr	0	0	0
20407	E014.11	drunkenness - pathological	1	curr	0	0	0
30404	E015.00	alcoholic paranoia	1	curr	0	0	0
33670	E01y.00	other alcoholic psychosis	1	0	0	0	0
2082	E01y000	alcohol withdrawal syndrome	1	curr	0	0	0
68111	E01yz00	other alcoholic psychosis nos	1	0	0	0	0
67651	E01z.00	alcoholic psychosis nos	1	0	0	0	0

For peer review only - http://bmjopen.bmj.com/site/about/guidelines.xhtml
For peer review only

CPRD medcode	Read code	Clinical term	1 Any alcohol-related code	2 Current drinking status	3 Codes indicating level of alcohol use	4 AUDIT screening	5 FAST screening
2084	E23..00	alcohol dependence syndrome	1	curr	0	0	0
2081	E23..11	alcoholism	1	curr	0	0	0
1399	E23..12	alcohol problem drinking	1	curr	H	0	0
5740	E230.00	acute alcoholic intoxication in alcoholism	1	curr	0	0	0
57714	E230.11	alcohol dependence with acute alcoholic intoxication	1	curr	0	0	0
40530	E230000	acute alcoholic intoxication, unspecified, in alcoholism	1	curr	0	0	0
56947	E230100	continuous acute alcoholic intoxication in alcoholism	1	curr	0	0	0
21624	E230200	episodic acute alcoholic intoxication in alcoholism	1	curr	0	0	0
59574	E230300	acute alcoholic intoxication in remission, in alcoholism	1	0	0	0	0
36296	E230z00	acute alcoholic intoxication in alcoholism nos	1	curr	0	0	0
31443	E231.00	chronic alcoholism	1	curr	0	0	0
37605	E231.11	Dipsomania	1	curr	0	0	0
43193	E231000	unspecified chronic alcoholism	1	curr	0	0	0
24064	E231100	continuous chronic alcoholism	1	curr	0	0	0
26106	E231200	episodic chronic alcoholism	1	curr	0	0	0
24485	E231300	chronic alcoholism in remission	1	0	0	0	0
33635	E231z00	chronic alcoholism nos	1	curr	0	0	0
6169	E23z.00	alcohol dependence syndrome nos	1	curr	0	0	0
7746	E250.00	nondependent alcohol abuse	1	curr	H	0	0
12271	E250.11	Drunkenness NOS	1	curr	0	0	0
27518	E250.12	hangover (alcohol)	1	curr	0	0	0
17777	E250.13	Inebriety NOS	1	curr	0	0	0
3782	E250.14	intoxication - alcohol	1	curr	0	0	0
669	E250000	nondependent alcohol abuse, unspecified	1	curr	H	0	0
23610	E250100	nondependent alcohol abuse, continuous	1	curr	H	0	0
12974	E250200	nondependent alcohol abuse, episodic	1	curr	H	0	0
31569	E250300	nondependent alcohol abuse in remission	1	0	0	0	0
28150	E250z00	nondependent alcohol abuse nos	1	curr	H	0	0
5611	Eu10.00	[x]mental and behavioural disorders due to use of alcohol	1	0	0	0	0
44299	Eu10000	[x]mental & behav dis due to use alcohol: acute intoxication	1	curr	0	0	0
9508	Eu10011	[x]acute alcoholic drunkenness	1	curr	0	0	0
21879	Eu10100	[x]mental and behav dis due to use of alcohol: harmful use	1	curr	0	0	0

For peer review only - http://bmjopen.bmj.com/site/about/guidelines.xhtml

CPRD medcode	Read code	Clinical term	1 Any alcohol-related code	2 Current drinking status	3 Codes indicating level of alcohol use	4 AUDIT screening	5 FAST screening
39327	Eu10200	[x]mental and behav dis due to use alcohol: dependence syndr	1	curr	0	0	0
28780	Eu10211	[x]alcohol addiction	1	curr	0	0	0
5758	Eu10212	[x]chronic alcoholism	1	curr	0	0	0
20514	Eu10300	[x]mental and behav dis due to use alcohol: withdrawal state	1	curr	0	0	0
64101	Eu10400	[X]Men & behav dis due alcohl: withdrawl state with delirium	1	curr	0	0	0
17259	Eu10411	[x]delirium tremens, alcohol induced	1	curr	0	0	0
12353	Eu10500	[x]mental & behav dis due to use alcohol: psychotic disorder	1	0	0	0	0
6467	Eu10511	[x]alcoholic hallucinosis	1	curr	0	0	0
65932	Eu10512	[x]alcoholic jealousy	1	curr	0	0	0
30162	Eu10513	[x]alcoholic paranoia	1	curr	0	0	0
17607	Eu10514	[x]alcoholic psychosis nos	1	0	0	0	0
39799	Eu10600	[x]mental and behav dis due to use alcohol: amnesic syndrome	1	0	0	0	0
11670	Eu10611	[x]korsakov's psychosis, alcohol induced	1	0	0	0	0
62000	Eu10700	[X]Men & behav dis due alcoh: resid & late-onset psychot dis	1	0	0	0	0
26323	Eu10711	[x]alcoholic dementia nos	1	0	0	0	0
37691	Eu10712	[x]chronic alcoholic brain syndrome	1	0	0	0	0
32927	Eu10800	[x]alcohol withdrawal-induced seizure	1	curr	0	0	0
45169	Eu10y00	[x]men & behav dis due to use alcohol: oth men & behav dis	1	0	0	0	0
64389	Eu10z00	[x]ment & behav dis due use alcohol: unsp ment & behav dis	1	0	0	0	0
36748	F11x011	alcoholic encephalopathy	1	0	0	0	0
33839	F144000	cerebellar ataxia due to alcoholism	1	0	0	0	0
30604	F25B.00	alcohol-induced epilepsy	1	0	0	0	0
2925	F375.00	alcoholic polyneuropathy	1	0	0	0	0
31742	F394100	alcoholic myopathy	1	0	0	0	0
4915	G555.00	alcoholic cardiomyopathy	1	0	0	0	0
8363	G852300	oesophageal varices in alcoholic cirrhosis of the liver	1	0	0	0	0
4506	J153.00	alcoholic gastritis	1	0	0	0	0
10691	J610.00	alcoholic fatty liver	1	0	0	0	0
3216	J611.00	acute alcoholic hepatitis	1	0	0	0	0
4743	J612.00	alcoholic cirrhosis of liver	1	0	0	0	0
21713	J612000	alcoholic fibrosis and sclerosis of liver	1	0	0	0	0
7885	J613.00	alcoholic liver damage unspecified	1	0	0	0	0

For peer review only - http://bmjopen.bmj.com/site/about/guidelines.xhtml
For peer review only

CPRD medcode	Read code	Clinical term	1 Any alcohol-related code	2 Current drinking status	3 Codes indicating level of alcohol use	4 AUDIT screening	5 FAST screening
17330	J613000	alcoholic hepatic failure	1	0	0	0	0
7943	J617.00	alcoholic hepatitis	1	0	0	0	0
7602	J617000	chronic alcoholic hepatitis	1	0	0	0	0
104611	J670800	alcohol-induced acute pancreatitis	1	0	0	0	0
24984	J671000	alcohol-induced chronic pancreatitis	1	0	0	0	0
66019	L254.11	suspect fetal damage from maternal alcohol	1	0	0	0	0
27670	L255300	maternal care for (suspected) damage to fetus from alcohol	1	0	0	0	0
72757	Q007100	fetus/neonate affected by placental/breast transfer alcohol	1	0	0	0	0
97916	Q007111	fetal alcohol syndrome	1	0	0	0	0
9169	R103.00	[d]alcohol blood level excessive	1	0	0	0	0
36687	SLH3.00	alcohol deterrent poisoning	1	0	0	0	0
19217	SM0..00	alcohol causing toxic effect	1	0	0	0	0
8984	SM00.00	ethyl alcohol causing toxic effect	1	0	0	0	0
48514	SM00100	denatured alcohol causing toxic effect	1	0	0	0	0
55536	SM00z00	ethyl alcohol causing toxic effect nos	1	0	0	0	0
88997	SM01.00	methyl alcohol causing toxic effect	1	0	0	0	0
11263	SM01100	wood alcohol causing toxic effect	1	0	0	0	0
106018	SM02.00	isopropyl alcohol causing toxic effect	1	0	0	0	0
102086	SM02200	rubbing alcohol causing toxic effect	1	0	0	0	0
64396	SM03000	amyl alcohol causing toxic effect	1	0	0	0	0
102321	SM03100	butyl alcohol causing toxic effect	1	0	0	0	0
101543	SM0y.00	other alcohol causing toxic effect	1	0	0	0	0
36499	SM0z.00	alcohol causing toxic effect nos	1	0	0	0	0
95410	SyuG000	[x]toxic effect of other alcohols	1	0	0	0	0
18252	T90..00	accidental poisoning by alcohol, nec	1	0	0	0	0
40541	T900.00	accidental poisoning by alcoholic beverages	1	0	0	0	0
15503	T901.00	accidental poisoning by other ethyl alcohol and its products	1	0	0	0	0
101426	T901z00	accidental poisoning by ethyl alcohol nos	1	0	0	0	0
37174	T902.00	accidental poisoning by methyl alcohol	1	0	0	0	0
60752	T903300	accidental poisoning by secondary propyl alcohol	1	0	0	0	0
53139	T90y.00	accidental poisoning by other alcohols	1	0	0	0	0
55415	T90z.00	accidental poisoning by alcohol nos	1	0	0	0	0

For peer review only - http://bmjopen.bmj.com/site/about/guidelines.xhtml
For peer review only

CPRD medcode	Read code	Clinical term	1 Any alcohol-related code	2 Current drinking status	3 Codes indicating level of alcohol use	4 AUDIT screening	5 FAST screening
21412	TJH3.00	adverse reaction to alcohol deterrents	1	0	0	0	0
31605	U1A9.00	[x]accident poisoning/exposure to alcohol	1	0	0	0	0
63306	U1A9000	[x]accident poison/exposure to alcohol at home	1	0	0	0	0
103069	U1A9200	[x]acc poison/expos alcohol school/pub admin area	1	0	0	0	0
104702	U1A9300	[x]accid pois/expos alcohol in sport/athletic area	1	0	0	0	0
63876	U1A9400	[x]accid poison/expos alcohol in street/highway	1	0	0	0	0
92908	U1A9500	[x]accid poison/expos alcohol trade/service area	1	0	0	0	0
104734	U1A9y00	[x]accid pois/expos to alcohol other spec place	1	0	0	0	0
61187	U1A9z00	[x]accid poison/expos to alcohol unspecif place	1	0	0	0	0
10463	U209.00	[x]intent self poison/exposure to alcohol	1	0	0	0	0
41638	U209000	[x]int self poison/exposure to alcohol at home	1	0	0	0	0
97085	U209y00	[x]int self poison alcohol other spec place	1	0	0	0	0
59414	U209z00	[x]intent self poison alcohol unspecif place	1	0	0	0	0
30036	U409.00	[x]poisoning/exposure, ? intent, to alcohol	1	0	0	0	0
68159	U409000	[x]poison/exposure ?intent, to alcohol at home	1	0	0	0	0
69407	U409200	[x]pois/exp ?intent alcohol school/pub admin area	1	0	0	0	0
96219	U409400	[x]pois/expos ?intent alcohol in street/highway	1	0	0	0	0
61190	U409z00	[x]pois/expos ?intent to alcohol unspecif place	1	0	0	0	0
73876	U60H300	[x]alcohol deterrents caus adverse effects in therapeut use	1	0	0	0	0
48241	U60H311	[x] adverse reaction to alcohol deterrents	1	0	0	0	0
44019	U80..00	[x]evidence of alcohl involv determin by blood alcohl level	1	0	0	0	0
12950	U800.00	[x]eviden of alcohl involv blood alcohl level <20 mg/100 ml	1	0	0	0	0
57202	U801.00	[x]eviden of alcohl involv blood alcohl level 20-39mg/100ml	1	0	0	0	0
59079	U802.00	[x]eviden of alcohl involv blood alcohl level 40-59mg/100ml	1	0	0	0	0
57242	U803.00	[x]eviden of alcohl involv blood alcohl level 60-79mg/100ml	1	0	0	0	0
53428	U804.00	[x]eviden of alcohl involv blood alcohl level 80-99mg/100ml	1	0	0	0	0
54504	U805.00	[x]eviden of alcoh involv blood alcoh level 100-119mg/100ml	1	0	0	0	0
32454	U806.00	[x]eviden of alcoh involv blood alcoh level 120-199mg/100ml	1	0	0	0	0
47907	U807.00	[x]eviden of alcoh involv blood alcoh level 200-239mg/100ml	1	0	0	0	0
39726	U808.00	[x]eviden alcoh involv blood alcoh level 240mg/100ml or more	1	0	0	0	0
103698	U80z.00	[x]evid alcoh invol detrm by pres alcoh in bld levl not spec	1	0	0	0	0
23978	U81..00	[x]evid of alcohol involv determind by level of intoxication	1	0	0	0	0

For peer review only - http://bmjopen.bmj.com/site/about/guidelines.xhtml
For peer review only

CPRD medcode	Read code	Clinical term	1 Any alcohol-related code	2 Current drinking status	3 Codes indicating level of alcohol use	4 AUDIT screening	5 FAST screening
105273	U810.00	[x]evid alcoh invol determ by levl of intox mild alcoh intox	1	0	0	0	0
73480	U811.00	[x]evid alcoh invol determ by level of intox mod alcoh intox	1	0	0	0	0
44686	U812.00	[x]evid alcoh invol determ by level of intox sev alcoh intox	1	0	0	0	0
39738	U813.00	[x]evid alcoh invl determ by levl intox very sev alcoh intox	1	0	0	0	0
41983	Z191.00	alcohol detoxification	1	0	0	0	0
46677	Z191100	alcohol withdrawal regime	1	0	0	0	0
61383	Z191200	planned reduction of alcohol consumption	1	curr	0	0	0
95181	Z191211	alcohol reduction programme	1	curr	0	0	0
64409	Z191400	self-monitoring of alcohol intake	1	0	0	0	0
30460	Z4B1.00	alcoholism counselling	1	curr	0	0	0
21829	Z786200	drinking practice	1	0	0	0	0
95650	ZC22100	advice to change drink intake	1	curr	0	0	0
97163	ZC22200	advice to change alcoholic drink intake	1	curr	0	0	0
54209	ZC2H.00	advice to change alcohol intake	1	curr	0	0	0
11140	ZG23100	advice on alcohol consumption	1	0	0	0	0
50507	ZR1E.00	alcohol dependence scale	1	0	0	0	0
40602	ZR1E.11	ads - alcohol dependence scale	1	0	0	0	0
32850	ZR1F.00	alcohol use disorders identification test	1	0	0	1	0
59873	ZR1F.11	audit - alcohol use disorders identification test	1	0	0	1	0
65980	ZR1G.00	alcohol use inventory	1	0	0	0	0
45557	ZR31.00	cage questionnaire	1	0	0	0	0
61750	ZR3f.00	comprehensive drinker profile	1	0	0	0	0
63457	ZR3f.11	cdp - comprehensive drinker profile	1	0	0	0	0
56441	ZRa1.00	michigan alcoholism screening test	1	0	0	0	0
69331	ZRa1.11	mast - michigan alcoholism screening test	1	0	0	0	0
99985	ZRa1100	brief michigan alcoholism screening test	1	0	0	0	0
41251	ZRa1111	bmast - brief michigan alcoholism screening test	1	0	0	0	0
70939	ZRaU.00	munich alcoholism test	1	0	0	0	0
22707	ZRBJ.00	drinking problem scale	1	0	0	0	0
46848	ZRBJ.11	dps - drinking problem scale	1	0	0	0	0
42305	ZRk6.00	severity of alcohol dependence questionnaire	1	0	0	0	0
66831	ZRk6.11	sadq - severity of alcohol dependence questionnaire	1	0	0	0	0

For peer review only - http://bmjopen.bmj.com/site/about/guidelines.xhtml
For peer review only

CPRD medcode	Read code	Clinical term	1 Any alcohol-related code	2 Current drinking status	3 Codes indicating level of alcohol use	4 AUDIT screening	5 FAST screening
62300	ZRk9.00	short alcohol dependence data	1	0	0	0	0
62299	ZRk9.11	sadd - short alcohol dependence data	1	0	0	0	0
10658	ZRLfD12	honos item 3 - alcohol/drug problem	1	0	0	0	0
66552	ZRR..00	inventory of drinking situations	1	0	0	0	0
59776	ZRVK.00	last six months of drinking questionnaire	1	0	0	0	0
7123	ZV11300	[v]personal history of alcoholism	1	0	0	0	0
16587	ZV11311	[v]problems related to lifestyle alcohol use	1	curr	H	0	0
7545	ZV4KC00	[v] alcohol use	1	0	0	0	0
8388	ZV57A00	[v]alcohol rehabilitation	1	0	0	0	0
8030	ZV6D600	[v]alcohol abuse counselling and surveillance	1	curr	0	0	0
61583	ZV70411	[v]medicolegal blood alcohol test	1	0	0	0	0
17149	ZV70L00	[v]blood-alcohol and blood-drug test	1	0	0	0	0
10458	ZV79100	[v]screening for alcoholism	1	0	0	0	0

For peer review only

Supplementary Table 2. Internal validity comparing alcohol consumption recorded up to 30 days before or after an AUDIT score, AUDIT-C score or measure of units per week.

	N	(%)	N patients with AUDIT score on the same date as record n	(%)	AUDIT score median	(IQR)	N patients with AUDIT C score on the same date as record n	(%)	AUDIT C score median	(IQR)	N patients with units per week recorded on same date as record n	(%)	Units per week median	(IQR)
**Current drinking status***														
Non	174,970	(20.3)	3053	(1.7)	0	(0-0)	1096	(0.6)	0	(0-0)	882	(0.5)	0	(0-1)
Ex	31,526	(3.7)	326	(1)	0	(0-0)	43	(0.1)	0	(0-0)	999	(3.2)	0	(0-1)
Current	655,834	(76.1)	24402	(3.7)	3	(1-5)	4707	(0.7)	3	(2-5)	333,402	(50.8)	6	(2-14)
**Current drinking level (Read codes)**														
Non-drinker	222835	(39.9)	3437	(1.5)	0	(0-0)	1145	(0.5)	0	(0-0)	2,715	(1.2)	0	(0-2)
Light drinker	255,919	(45.8)	3,401	(1.3)	2	(1-3)	1,131	(0.4)	2	(1-3)	19,777	(7.7)	3	(1-8)
Moderate drinker	52,070	(9.3)	1,205	(2.3)	1	(0-4)	236	(0.5)	3	(2-4)	13,024	(25)	6	(2-14)
Heavy drinker	27,923	(5)	2,906	(10.4)	6	(5-9)	127	(0.5)	8	(6-9)	10,572	(37.9)	18	(10-30)
**Units per week**														
0	38,929	(9)	1,919	(4.9)	0	(0-0)	159	(0.4)	0	(0-1)	n/a		n/a	
1-14	298,441	(69.2)	11,920	(4)	3	(2-4)	2,273	(0.8)	3	(2-4)				
15-42	80,449	(18.6)	2,983	(3.7)	6	(4-8)	412	(0.5)	5	(4-7)				
43+	13,575	(3.1)	248	(1.8)	10	(8-12)	19	(0.1)	8	(5-10)				
**AUDIT C score category**														
0-4; Low risk	16,206	(76.8)	4,006	(24.7)	2	(0-3)	n/a		n/a		2,120	(13.1)	4	(2-8)
5-12; High risk	4,893	(23.2)	1,160	(23.7)	6	(5-7)					901	(18.4)	12	(7-20)
**AUDIT score category**														
0-7; Alcohol Education	71,401	(92.5)	n/a		n/a		4,899	(6.9)	2	(1-4)	15,852	(22.2)	4	(2-10)
8-15; Simple Advice	5,405	(7)					242	(4.5)	9	(8-10)	1,605	(29.7)	20	(14-30)
16-19; Simple Advice plus Brief Counseling and Continued Monitoring	206	(0.3)					0	(0)		(-)	49	(23.8)	24	(16-48)
20-40; Referral to Specialist for Diagnostic Evaluation and Treatment	200	(0.3)					0	(0)		(-)	30	(15)	47	(20-80)

*based on recorded status in Read codes and Additional details (not including derived status based on units consumed or AUDIT score)

For peer review only

For peer review only - http://bmjopen.bmj.com/site/about/guidelines.xhtml

References

1. Denaxas, S.C., et al., *Data resource profile: cardiovascular disease research using linked bespoke studies and electronic health records (CALIBER).* Int J Epidemiol, 2012. **41**(6): p. 1625-38.

2. Department of Health, *UK Chief Medical Officers' alcohol guidelines review: Summary of the proposed new guidelines.* 2016.

3. Babor, T.F., et al. *The Alcohol Use Disorders Identification Test: Guidelines for Use in Primary Care*. 2001; Available from: http://www.talkingalcohol.com/files/pdfs/WHO_audit.pdf.

For peer review only

The RECORD statement – checklist of items, extended from the STROBE statement, that should be reported in observational studies using routinely collected health data.

	Item No.	STROBE items	Location in manuscript where items are reported	RECORD items	Location in manuscript where items are reported
**Title and abstract**					
	1	(a) Indicate the study's design with a commonly used term in the title or the abstract (b) Provide in the abstract an informative and balanced summary of what was done and what was found	Page 1	RECORD 1.1: The type of data used should be specified in the title or abstract. When possible, the name of the databases used should be included.<br><br>RECORD 1.2: If applicable, the geographic region and timeframe within which the study took place should be reported in the title or abstract.<br><br>RECORD 1.3: If linkage between databases was conducted for the study, this should be clearly stated in the title or abstract.	Page 2<br><br>Page 2<br><br>n/a
**Introduction**					
Background rationale	2	Explain the scientific background and rationale for the investigation being reported	Page 4		
Objectives	3	State specific objectives, including any prespecified hypotheses	Page 5		
**Methods**					
Study Design	4	Present key elements of study design early in the paper	Page 6		
Setting	5	Describe the setting, locations, and relevant dates, including periods of recruitment, exposure, follow-up, and data collection	Page 6		

For peer review only

Participants	6	*(a) Cohort study* - Give the eligibility criteria, and the sources and methods of selection of participants. Describe methods of follow-up<br>*Case-control study* - Give the eligibility criteria, and the sources and methods of case ascertainment and control selection. Give the rationale for the choice of cases and controls<br>*Cross-sectional study* - Give the eligibility criteria, and the sources and methods of selection of participants<br><br>*(b) Cohort study* - For matched studies, give matching criteria and number of exposed and unexposed<br>*Case-control study* - For matched studies, give matching criteria and the number of controls per case	Page 6	RECORD 6.1: The methods of study population selection (such as codes or algorithms used to identify subjects) should be listed in detail. If this is not possible, an explanation should be provided.<br><br>RECORD 6.2: Any validation studies of the codes or algorithms used to select the population should be referenced. If validation was conducted for this study and not published elsewhere, detailed methods and results should be provided.<br><br>RECORD 6.3: If the study involved linkage of databases, consider use of a flow diagram or other graphical display to demonstrate the data linkage process, including the number of individuals with linked data at each stage.	Page 6
Variables	7	Clearly define all outcomes, exposures, predictors, potential confounders, and effect modifiers. Give diagnostic criteria, if applicable.	Page 7	RECORD 7.1: A complete list of codes and algorithms used to classify exposures, outcomes, confounders, and effect modifiers should be provided. If these cannot be reported, an explanation should be provided.	Appendices and online via link
Data sources/ measurement	8	For each variable of interest, give sources of data and details of methods of assessment (measurement).<br>Describe comparability of assessment methods if there is more than one group	Page 7, online via link on page 7		

For peer review only

Bias	9	Describe any efforts to address potential sources of bias	Discussion		
Study size	10	Explain how the study size was arrived at	Appendix Figure 1		
Quantitative variables	11	Explain how quantitative variables were handled in the analyses. If applicable, describe which groupings were chosen, and why	Page 6, page 11		
Statistical methods	12	(a) Describe all statistical methods, including those used to control for confounding<br>(b) Describe any methods used to examine subgroups and interactions<br>(c) Explain how missing data were addressed<br>(d) *Cohort study* - If applicable, explain how loss to follow-up was addressed<br>*Case-control study* - If applicable, explain how matching of cases and controls was addressed<br>*Cross-sectional study* - If applicable, describe analytical methods taking account of sampling strategy<br>(e) Describe any sensitivity analyses	Pages 8-9		
Data access and cleaning methods		..		RECORD 12.1: Authors should describe the extent to which the investigators had access to the database population used to create the study population.	Page 24

For peer review only

				RECORD 12.2: Authors should provide information on the data cleaning methods used in the study.	
Linkage		..		RECORD 12.3: State whether the study included person-level, institutional-level, or other data linkage across two or more databases. The methods of linkage and methods of linkage quality evaluation should be provided.	n/a
**Results**					
Participants	13	(a) Report the numbers of individuals at each stage of the study (*e.g.*, numbers potentially eligible, examined for eligibility, confirmed eligible, included in the study, completing follow-up, and analysed)<br>(b) Give reasons for non-participation at each stage.<br>(c) Consider use of a flow diagram		RECORD 13.1: Describe in detail the selection of the persons included in the study (*i.e.,* study population selection) including filtering based on data quality, data availability and linkage. The selection of included persons can be described in the text and/or by means of the study flow diagram.	Page 6
Descriptive data	14	(a) Give characteristics of study participants (*e.g.*, demographic, clinical, social) and information on exposures and potential confounders<br>(b) Indicate the number of participants with missing data for each variable of interest<br>(c) *Cohort study* - summarise follow-up time (*e.g.*, average and total amount)	Table 1<br><br>Table 1		
Outcome data	15	*Cohort study* - Report numbers of outcome events or summary measures over time<br>*Case-control study* - Report numbers in each exposure	Table 1		

For peer review only

		category, or summary measures of exposure *Cross-sectional study* - Report numbers of outcome events or summary measures			
Main results	16	(a) Give unadjusted estimates and, if applicable, confounder-adjusted estimates and their precision (e.g., 95% confidence interval). Make clear which confounders were adjusted for and why they were included (b) Report category boundaries when continuous variables were categorized (c) If relevant, consider translating estimates of relative risk into absolute risk for a meaningful time period	n/a		
Other analyses	17	Report other analyses done—e.g., analyses of subgroups and interactions, and sensitivity analyses	Pages 13-15		
**Discussion**					
Key results	18	Summarise key results with reference to study objectives	Page 16		
Limitations	19	Discuss limitations of the study, taking into account sources of potential bias or imprecision. Discuss both direction and magnitude of any potential bias	Page 19	RECORD 19.1: Discuss the implications of using data that were not created or collected to answer the specific research question(s). Include discussion of misclassification bias, unmeasured confounding, missing data, and changing eligibility over time, as they pertain to the study being reported.	Page 19
Interpretation	20	Give a cautious overall interpretation of results considering objectives,	Pages 20		

For peer review only - http://bmjopen.bmj.com/site/about/guidelines.xhtml

		limitations, multiplicity of analyses, results from similar studies, and other relevant evidence			
Generalisability	21	Discuss the generalisability (external validity) of the study results	Page 20		
**Other Information**					
Funding	22	Give the source of funding and the role of the funders for the present study and, if applicable, for the original study on which the present article is based	Page 24		
Accessibility of protocol, raw data, and programming code		..		RECORD 22.1: Authors should provide information on how to access any supplemental information such as the study protocol, raw data, or programming code.	Page 24

*Reference: Benchimol EI, Smeeth L, Guttmann A, Harron K, Moher D, Petersen I, Sørensen HT, von Elm E, Langan SM, the RECORD Working Committee. The REporting of studies Conducted using Observational Routinely-collected health Data (RECORD) Statement. *PLoS Medicine* 2015; in press.

*Checklist is protected under Creative Commons Attribution (CC BY) license.

For peer review only
